# Transcriptional and Small RNA Responses of the White Mold Fungus *Sclerotinia sclerotiorum* to Infection by a Virulence-Attenuating Hypovirus

**DOI:** 10.3390/v10120713

**Published:** 2018-12-14

**Authors:** Shin-Yi Lee Marzano, Achal Neupane, Leslie Domier

**Affiliations:** 1 Department of Biology and Microbiology, South Dakota State University, Brookings, SD 57006, USA; achal.neupane@sdstate.edu (A.N.); 2 Department of Agronomy, Horticulture, and Plant Science, South Dakota State University, Brookings, SD 57006, USA; 3 United States Department of Agriculture, Agricultural Research Service, Department of Crop Sciences, University of Illinois, Urbana, IL 61801, USA

**Keywords:** hypovirus, small RNA, tRFs, mycovirus

## Abstract

Mycoviruses belonging to the family *Hypoviridae* cause persistent infection of many different host fungi. We previously determined that the white mold fungus, *Sclerotinia* *sclerotiorum*, infected with Sclerotinia sclerotiorum hypovirus 2-L (SsHV2-L) exhibits reduced virulence, delayed/reduced sclerotial formation, and enhanced production of aerial mycelia. To gain better insight into the cellular basis for these changes, we characterized changes in mRNA and small RNA (sRNA) accumulation in *S.* *sclerotiorum* to infection by SsHV2-L. A total of 958 mRNAs and 835 sRNA-producing loci were altered after infection by SsHV2-L, among which >100 mRNAs were predicted to encode proteins involved in the metabolism and trafficking of carbohydrates and lipids. Both *S*. *sclerotiorum* endogenous and virus-derived sRNAs were predominantly 22 nt in length suggesting one dicer-like enzyme cleaves both. Novel classes of endogenous small RNAs were predicted, including phasiRNAs and tRNA-derived small RNAs. Moreover, *S*. *sclerotiorum* phasiRNAs, which were derived from noncoding RNAs and have the potential to regulate mRNA abundance in trans, showed differential accumulation due to virus infection. tRNA fragments did not accumulate differentially after hypovirus infection. Hence, in-depth analysis showed that infection of *S*. *sclerotiorum* by a hypovirulence-inducing hypovirus produced selective, large-scale reprogramming of mRNA and sRNA production.

## 1. Introduction

Fungal viruses (mycoviruses) are highly diverse, infect pathogenic and nonpathogenic fungi, and can significantly reduce the virulence of pathogenic fungi [1]. Fungal–virus interactions involve the interplay between gene expression networks, some of which are influenced by small noncoding RNAs [2]. Central to those interactions are RNA-mediated antiviral defenses that are activated by double-stranded RNA (dsRNA) in a process termed RNA silencing [3,4,5]. Specificity of the defense is imparted by short (21–24 nt) interfering RNAs (siRNAs) produced from viral dsRNA by RNase III-type enzymes called dicer-like (DCL) proteins [6]. One strand of each siRNA is combined with Argonaute (AGO) proteins into RNA-induced silencing complexes and directs degradation of complementary viral RNA sequences [3].

While RNA silencing genes are highly conserved in animals and plants, they are much less conserved in fungi and oomycetes [7]. For example, the genomes of *Saccharomyces cerevisiae* and *Ustilago maydis* lack homologs of canonical AGO and DCL genes, while they are present in the genomes of *Saccharomyces castellii* and *Ustilago hordei* [8]. In addition, some fungi, e.g., *Candida albicans*, have evolved novel DCL proteins that differ significantly from those of higher eukaryotes [9], making it challenging to ascertain the importance of fungal endogenous small RNA processing in response to virus infection.

Endogenous small RNAs (sRNAs) play important roles in gene regulation in development and responses to biotic and abiotic stresses in animals and plants [10], but their roles are not well defined in phytopathogenic fungi. It remains elusive whether filamentous fungi have functional microRNAs (miRNAs), typically 21–24 nt in length, generated by DCL processing of short hairpin structures and regulate gene expression directly through interactions with AGO and indirectly through the production of secondary phased siRNAs (phasiRNAs) from long noncoding RNAs [11,12,13]. PhasiRNAs are a class of secondary sRNAs triggered by miRNAs; the substrate of phasiRNAs is produced by host RNA-dependent RNA polymerase (RDR). The presence of phasiRNAs is an indication of functional miRNAs.

While most plants share a common set of miRNAs [10], fungal species analyzed so far however express diverse suites of miRNAs [14,15]. A few studies have attempted to identify the roles fungal miRNAs play in regulating gene expression. Small RNA and high-throughput rapid amplification of cDNA ends (HT-RACE) to identify cleavage targets for *Fusarium oxysporum* microRNA-like RNAs (milRNAs) did not identify any milRNAs that were present in the current miRNA database, and none of the milRNAs were predicted to trigger cleavage of *F*. *oxysporum* mRNAs [16]. Analysis of differentially expressed sRNA loci and miRNA accumulation in *Aspergillus flavus* under different growth conditions suggested that miRNAs play important roles in cellular functions including mycotoxin biosynthesis and mycelial growth [17]. The predicted targets for sRNAs from the wheat stripe rust fungus (*Puccinia striiformis*) were enriched for kinases and small secreted proteins suggesting that development-related signaling pathways are regulated by sRNAs in *P*. *striiformis* [14].

Another source of abundant sRNAs is derived from tRNAs. In plants and animals, mature tRNAs have been confirmed to be sources of functional sRNAs produced in a DCL-independent manner that have been implicated in post-transcriptional and epigenetic regulation of gene expression and repression of retrotransposons [18,19,20]. In the plant-pathogenic fungus *Magnaporthe oryzae*, tRNA-derived RNA fragments (tRFs) were more abundant in appressoria—specialized-infection tissues—than in vegetative mycelia [21], suggesting that small RNAs in fungi also play active roles in the regulation of growth and development as in higher eukaryotes.

Virus-derived siRNAs (vsiRNAs) have been shown to direct the cleavage of host RNAs in plants [22,23,24,25]. Also, another new class of endogenous small RNAs were shown to be activated in *Arabidopsis* plants in response to infection by cucumber mosaic virus that were termed virus-activated siRNAs (vasiRNAs) that lead to the silencing of a broad set of host genes and establishment of an antiviral state [26]. Clearly, small RNA are not just products of RNA silencing but instead exhibit biological functions to down regulate gene expression.

Changes in fungal gene expression in response to virus infection vary greatly depending on the nature of the host–virus interaction that hinges on the activity of antiviral RNA silencing pathway. Saccharomyces cerevisiae LA virus, a member of the Totiviridae, and its M1 satellite impart a beneficial killer phenotype to *S*. *cerevisiae*, which lacks RNA silencing genes, and produces relatively small changes in gene expression [27]. In contrast, infection of plant pathogenic fungi with robust RNA silencing by mycoviruses that alter fungal virulence have much more pronounced effects on fungal gene expression. For example, infection of *Cryphonectria parasitica* with Cryphonectria hypovirus 1 (CHV1) significantly altered the expression of more than 13% of the analyzed transcripts [28]. Proteomic analysis of proteins secreted by CHV1-infected *C*. *parasitica* identified 99 proteins with differential accumulation relative to virus-free cultures [29]. Similar proteomic analysis of infection of *Fusarium graminearum* by Fusarium graminearum virus DK21, which perturbs development and attenuates the virulence of the fungus, altered the accumulation of nearly 150 proteins [30].

High-throughput (HT) sequencing of different RNA species provides an approach to functionally characterize how small RNAs interact with potential targets to determine whether milRNAs and siRNAs repress gene expression by mRNA cleavage in fungi. The combination of three types of sequencing data—small RNA-seq, HT-5′-RACE (degradome), and mRNA-seq—allows a global analysis of small RNA function. By analyzing predicted milRNA sequences together with mapped degradome reads, high confidence cleavage events can be identified. Predicted cleavage events can be supported by observations of downregulation of predicted mRNA targets in RNA-seq analysis. Confirmation of predictions is especially important in fungi because, unlike plant or animal miRNAs, fungal pre-milRNAs are do not have well-defined secondary structures and the thermodynamics of the interactions between miRNAs and targets is not well defined, which can lead to false positive cleavage site predictions that cannot be confirmed empirically. 

Because mycoviruses can reduce fungal virulence to animal and plant hosts, we aimed to examine the effect of mycovirus infection on gene expression and different classes of sRNA accumulation and gather functional evidence for the existence of miRNAs in the white mold fungus *Sclerotinia sclerotiorum*, a devastating plant pathogen. Previously, we determined that Sclerotinia sclerotiorum hypovirus 2-L (SsHV2-L) reduced/delayed development in sclerotia, enhanced production of aerial mycelia, and induced hypovirulence. Now, we report the use of high throughput sequencing to (1) show that infection by SsHV2-L changes in mRNA and sRNA accumulation in *S*. *sclerotiorum*; (2) identify new classes of fungal sRNAs; and (3) examine whether predicted sRNA cleavage events correspond to changes in gene expression. Supported by HT-RACE analysis, we show that endogenous *S*. *sclerotiorum* sRNAs were capable of directing the cleavage of coding and noncoding RNAs, the latter leading to the production of phasiRNAs, demonstrating the evidence of miRNA activity.

## 2. Materials and Methods

### 2.1. Preparation of Sclerotinia sclerotiorum Cultures and RNA Extraction

Virus-free (VF) and virus-transfected (VT) cultures of *S. sclerotiorum* strain SsDK3 were produced as described previously [31]. Two sequencing runs were performed: Trial #1 and Trial #2. For the sequencing run of 4 libraries (Trial #1), fungal cultures were grown in potato dextrose broth (PDB) at 25 °C for 10 d. For the separate sequencing run of 10 libraries (Trial #2), fungal cultures were grown on potato dextrose agar (PDA) at 21 °C for 4 d. Total RNAs were extracted from VF and VT cultures using RNeasy Mini (Qiagen, Valencia, CA, USA). Small RNAs were extracted from two biological replications each for VF and VT cultures using mirVana miRNA isolation kits (ThermoFisher Scientific, Waltham, MA, USA) following the manufacturer’s instructions.

### 2.2. Analysis of S. sclerotiorum Transcriptome

Trial #1 of four RNA-Seq libraries (two VF and two VT) was prepared using the TruSeq Stranded mRNA Library Prep Kit (Illumina, San Diego, CA, USA). The RNA-Seq libraries were barcoded and sequenced as single-end 100-nt reads in one lane on an Illumina HiSeq2500. Trial #2 of ten RNA-Seq libraries (five VF and five VT) were prepared using the same kit and barcoded/sequenced as single-end 100-nt reads on an Illumina HiSeq4000. All RNA-Seq reads were assembled de novo using Trinity [32] and used to identify novel coding regions in the *S. sclerotiorum* genome using BRAKER1 [33]. The abundance of reads aligning to predicted coding regions was estimated using RSEM [34]. Differentially expressed coding regions were identified using the DESeq2 Bioconductor package [35]. Differentially expressed coding regions were classified using PANTHER [36]. The relative abundance of transcripts from *S. sclerotiorum* coding regions was determined using reads per kilobase of coding sequence per million mapped reads (RPKM) [37].

### 2.3. Analysis of S. sclerotiorum Small RNA Populations

Four sRNA libraries (two VF and two VT) were prepared using the TruSeq Small RNA Library Prep Kit (Illumina). The four sRNA libraries were barcoded and sequenced in one lane as 50-nt single-end reads on an Illumina HiSeq2500. Loci with significantly different accumulations of sRNAs between VF and VT samples were identified using DESeq2. Candidate miRNAs were predicted with six different programs, miRDeep2 [38], MiRDeep* [39], miRDeep-P [40], miReap (http://sourceforge.net/projects/mireap/), MiRPlant [41], and ShortStack [42], using the default settings for each program. Small RNA sequences with fewer than 20 reads, and sequences that aligned to coding regions or ribosomal RNA or more than 10 locations in the *S. sclerotiorum* genome were excluded. Loci producing phased sRNAs were identified with Shortstack [43]. Plots of phased siRNAs were prepared using the equation described by Howell et al. [44]. Candidate tRNA genes in the *S. sclerotiorum* genome were predicted using the tRNAscan-SE server [45].

### 2.4. High-Throughput RNA Ligase-Mediated Rapid Amplification of cDNA Ends (HT-RACE) Analysis

Four HT-RACE (degradome) libraries (two VF and two VT) were constructed as described by Li et al. [46]. Briefly, the Illumina sRNA-seq 5′ adapter was ligated to the purified polyadenylated RNAs from two VF and two VT cultures using T4 RNA ligase. The 5′ termini of most intact fungal mRNAs are blocked by an m7GTP cap structure, while cleaved RNAs have a 5′ terminal phosphate that is available for ligation with the adapter. The 5′-adapter-ligated RNAs were purified, fragmented, treated with phosphatase, purified, and ligated to the Illumina sRNA-seq 3′ adapter. The dual-adapter ligated RNAs were reverse transcribed using the Illumina sRNA-seq RT primer and amplified with the Illumina Gx1 and GX2 sRNA-seq PCR primers. The four barcoded libraries were pooled and sequenced in one lane for 50-nt single-end reads. Potential targets for candidate miRNAs and cluster sequences were identified using the HT-RACE sequence data and the predicted *S*. *sclerotiorum* coding sequences with the CleaveLand4 pipeline [47]. Predicted miRNA targets with *P*-values of less than 0.05 and degradome categories 0 and 1 hits were selected for downstream cluster analysis.

## 3. Results

### 3.1. Changes in mRNA Accumulation Associated with Infection of S. sclerotiorum by SsHV2-L

Two sequencing runs of the transcriptome were performed. Trial #1 had two biological replications each, and Trial #2 had five biological replications each. The accession numbers for the infected and virus free treatments from the Trial #1 are SRR8306347 and SRR8306348, respectively, while SRR8305679 and SRR8305680 are the accession numbers for the infected and virus free treatments from the Trial #2, respectively.

RNA-Seq analysis of Trial #1 produced a total of 2.11 × 10^8^ 100-nt reads from the four libraries. On average, 89.9% and 87.1% of the reads from the virus-free and SsHV2-L-infected libraries aligned to the *S. sclerotiorum* genome sequence [48], respectively (Table S1a), and assembled into 23,968 contigs with an N50 of 2834 nt. RNA-Seq analysis of Trial #2 produced a total of 3.99 × 10^8^ 100-nt reads from the ten libraries with 89.0% and 83.7% of the reads from the virus free and SsHV2-L infected libraries aligned to the *S*. *sclerotiorum* genome sequence, respectively (Table S1b). The *S. sclerotiorum* genome is predicted to contain 14,053 coding regions [48], but re-annotation of the *S. sclerotiorum* genome using the RNA-Seq data identified an additional 174 putative coding regions with an average size of 680 nt in this study. Overall, in the RNAs from the infected samples, an average of 1.45% and 5.7% of the sequence reads were derived from the SsHV2-L genome from Trial #1 and Trial #2, respectively.

The RNA-Seq data analyzed from Trial #1 when the cultures are 10-d old in PDB at 25 °C, among the proteins differentially expressed primarily involved in carbohydrate metabolism, *Sclerotinia sclerotiorum* hexose transporter 1 (Sshxt1; SS1G_04841), and a predicted invertase (SS1G_07184; beta-fructofuranosidase) were upregulated 37.8-fold and 18.8-fold, respectively, in SsHV2-L-infected cultures. The expression of *S. sclerotiorum* hexose transporter 2 (SS1G_13734) was upregulated 89.3-fold by SsHV2-L infection. In Trial #1, 958 coding regions differed significantly; 471 were upregulated and 487 were downregulated (Table S2a). The same trial also found that SsHV2-L infection significantly enhanced the accumulation of mRNAs predicted to encode a DEAD-box ATP-dependent RNA helicase (SS1G_04322) and a mitochondria-localized pentatricopeptide repeat-containing protein (SS1G_08638) (Table S2a). Similar to plant gene expression changes induced by plant virus infection [49], mycovirus SsHV2-L infection also increased transcription levels of retrotransposon-related coding sequences in the fungus. SsHV2-L enhanced expression of coding regions for ubiquitin-related proteins (SS1G_02395 and SS1G_00267), aldo/keto reductase (SS1G_00727), and a heat shock protein (SS1G_12897). SsHV2-L infection altered the accumulation of mRNAs encoding 10 cytochrome P450s and 13 methyltransferases. SsHV2-L downregulated SS1G_01530 which is a putative SPX-domain protein in a manner similar to the *S. cerevisiae* protein SPX-domain protein Syg1 that interacts with the β subunit of G-proteins to suppress the lethality of α subunit deficiency [50].

The two trials of transcriptome sequencing runs from *S. sclerotiorum* grown on different media, for different times and at different temperatures generated very different lists of differentially expressed genes in response to SsHV2-L infection. Both sequencing runs showed that infection by SsHV2-L did not significantly alter the expression of *S. sclerotiorum* homologs of AGO (SS1G_00334 and SS1G_11723), DCL (SS1G_13747 and SS1G_10369), or RDR (SS1G_03377, SS1G_13161 and SS1G_09915). With the ten libraries of Trial #2 RNA-Seq analysis from 4-d old cultures grown on PDA at 21 °C, infection of *S. sclerotiorum* with SsHV2-L significantly (*p* < 0.05) altered the abundance of transcripts from 1319 coding regions; 779 were upregulated and 540 were downregulated. Panther analysis of the functional classifications of the annotated *S. sclerotiorum* coding regions found that sequences predicted to be involved in rRNA metabolic processes (*P* = 6.4 × 10^−20^) and cellular component biogenesis (*P* = 4.6 × 10^−13^) were significantly over represented in the SsHV2-L-infected cultures. rRNA-processing protein 7 homolog A-related protein (SS1G_00995) was downregulated by 2.8-fold. Ribosomal U3 small nucleolar ribonucleoprotein proteins IMP3—SS1G_05011 and SS1G_05012—were both downregulated ~2.5-fold. SS1G_03709 and SS1G_00849, which are predicted to be virulence effectors when infecting soybean [51], were downregulated by 5.6-fold upon SsHV2-L infection. Several methyltransferases were downregulated, including SS1G_12790 by 4.9-fold and SS1G_11246 and SS1G_01682 both by 4-fold (Table S2b). Inactive methyltransferase may lead to terminal modification of small RNAs that have been found to trigger phasiRNA production in plants [52].

### 3.2. Small RNA Accumulation in Healthy and SsHV2-L-infected S. sclerotiorum Cultures

Sequence analysis of the four sRNA libraries produced a total of 9.04 × 10^7^ reads of 17 to 36 nt, of which, 63.5% aligned to the *S. sclerotiorum* genome sequence (Table S3). The accession numbers are SRR8306349 and SRR8306350 for the virus-infected and virus-free treatments, respectively. On average, 16.5% of the sRNA reads aligned to the 192 *S. sclerotiorum* tRNAs predicted by tRNAscan-SE [45], 12.9% to ribosomal RNAs, 5.9% to retrotransposons, and similar to *Fusarium oxysporum* and *N. crassa*, 12.9% of the *S. sclerotiorum* sRNA reads aligned to the *S. sclerotiorum* mitochondrial genome [16,53,54]. In the RNAs from the two infected samples 9.0% of the sequence reads were derived from the SsHV2-L genome on average. 

**Figure 1:**
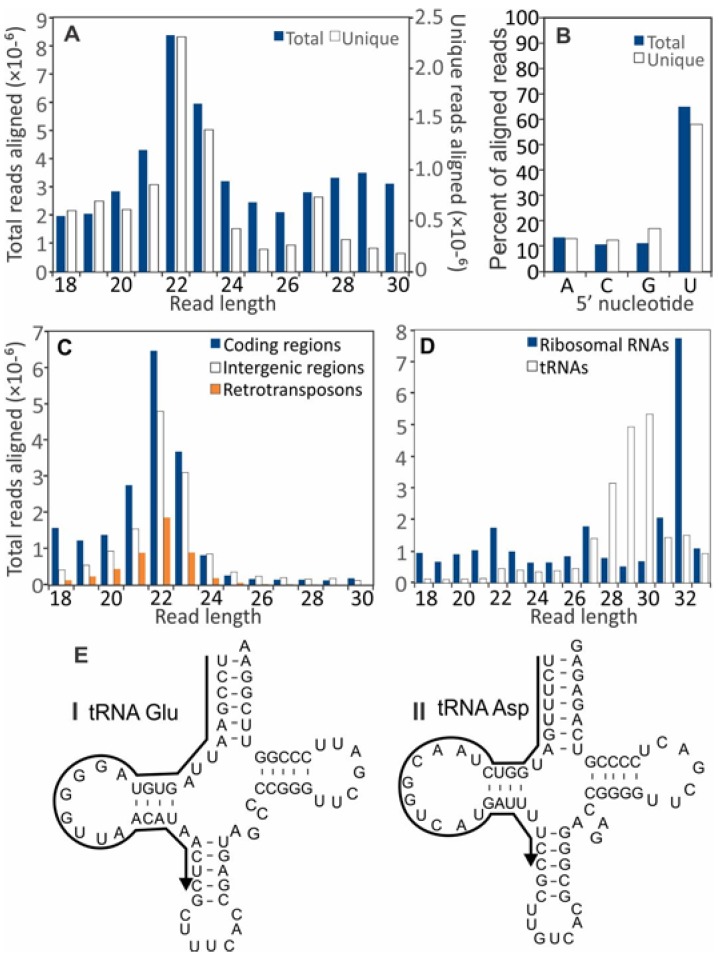
Size distributions of small RNA sequences that aligned to the *S*. *sclerotiorum* genome. (**A**) Size distribution of small RNA libraries from combined virus-free and hypovirus-infected *S*. *sclerotiorum* cultures. White and black columns represent unique and total reads of the sRNAs, respectively. (**B**) Frequency of 5′ terminal nucleotides from pooled small RNA samples. Size distribution of small RNA reads aligning to (**C**) coding regions, intergenic regions, retrotransposon sequences, and (**D**) ribosomal RNA and tRNA sequences. (**E**) Mature tRNA structures predicted by tRNAscan-SE with sequences of the two most abundant small RNA sequences that resembled stress-induced tRNA halves. I: 4.5 × 106 reads (5.0% of total reads); tRNA Glu-derived tRF5-Glu(GAA) on *S*. *sclerotiorum* chromosomes 4, 7, and 16 (two copies); and *B*. *cinerea* chromosomes 2, 8, 14, and 16 (two copies). II: 1.6 × 106 reads (1.9%); tRNA Asp on *S*. *sclerotiorum* chromosomes 1, 5, 11, 12, and 14; and *B*. *cinerea* chromosomes 9, 10, and 13 (two copies).

In all four samples, 22-nt sRNAs were the most abundant with a strong preference for uracil at the 5′ position (Figure 1B). Among the reads that aligned to unique positions in the *S. sclerotiorum* genome, there was a second peak at 27-nt (Figure 1A). Most of the 27-nt sRNAs aligned to *S. sclerotiorum* ribosomal RNA genes (Figure 1D). Few of the 27-nt sRNAs aligned to coding regions or sequences with homology to retrotransposons (Figure 1C). The density with which reads aligned to the genome sequence was highly variable. Twenty of the 25 most abundant nonribosomal RNA and noncoding sequences were of 27- to 32-nt in length. Most aligned to three to six loci in the *S. sclerotiorum* genome and many were conserved in the genome of *B. cinerea*. For example, the most abundant sequence (5′-UCCGAAUUAGUGUAGGGGUUAACAUAACUC-3′) with over 4.5 × 10^6^ reads (5.0% of total reads) aligned to one locus each on *S. sclerotiorum* chromosomes 4 and 7, and two loci on chromosome 16 that corresponded to the locations of predicted glutamine tRNAs (Figure 1E). The sequence, designated as tRF5-Glu(GAA), also aligned to predicted glutamine tRNAs on *B. cinerea* chromosomes 2, 8, and 14 and two loci on chromosome 16 (data not shown). Hence the highly abundant sRNAs were likely derived from mature tRNAs and probably represented tRNA halves that are produced by endonucleolytic cleavage of mature tRNAs, sometimes in response to biotic and abiotic stresses [18]. However, the abundance of sRNAs derived from tRNAs was on average 1.4-fold lower in infected-SsHV2-L samples than virus-free controls. 

**Figure 2:**
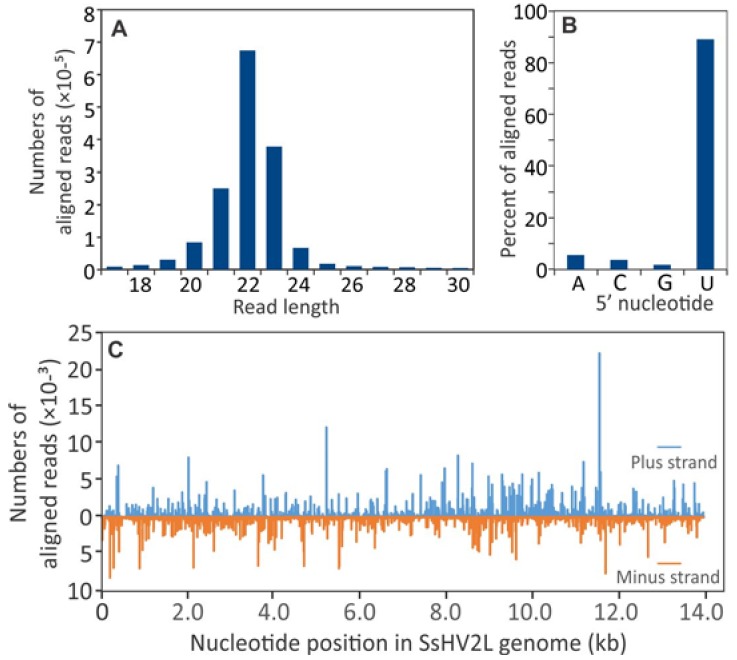
Size distribution of small RNA sequences that aligned to the Sclerotinia sclerotiorum hypovirus 2 L (SsHV2L) genome sequence. (**A**) Size distribution and (**B**) frequency of 5′ terminal nucleotides of small RNAs that aligned to the SsHV2L genome. (**C**) Distribution of small RNA reads that aligned to the SsHV2L genome. Bars above zero indicate alignment to the positive strand, and bars below zero indicate alignment to the negative strand.

Similar to sRNA reads derived from the *S. sclerotiorum* genome, the predominant size of sRNAs derived from the SsHV2-L genome were 22 nt in length (Figure 2A) and nearly 90% of the aligned reads contained a 5′-terminal U residue (Figure 2B). As has been reported for other virus infections, the sRNA reads were derived nonuniformly from both RNA strands (Figure 2C).

### 3.3. Identification of S. sclerotiorum Loci Producing microRNA-Like RNAs

**Figure 3:**
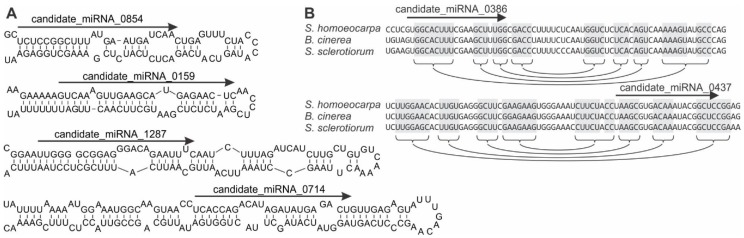
Examples of sRNA producing loci in the *S*. *sclerotiorum* genome (**A**) capable of folding into structures similar to pre-microRNA and (**B**) conserved in the genomes of other members of the family Sclerotiniaceae. Arrows indicate the positions of mature microRNA-like sequences. Connected shaded boxes indicate regions of conserved base pairing in predicted stem-and-loop structures.

Cluster analysis in ShortStack identified 8617 intergenic loci that produced at least 100 sRNA reads. Nearly 10% (835) of the intergenic loci producing sRNAs responded significantly to SsHV2-L infection. Because of the variability in RNA silencing-related genes in fungi, candidate miRNAs were predicted from the sRNA data using six different programs. The analysis predicted a total of 459 candidate miRNAs in noncoding and nonribosomal regions of the *S. sclerotiorum* genome with at least 20 reads in the combined data set. Notably, the numbers of candidate miRNAs predicted differed for each of the ten programs used because of the evolution position of fungi between plants and animals. For example, miRDeep2 [38], MiRDeep* [39], miRDeep-P [40], miReap (http://sourceforge.net/projects/mireap/), MiRPlant [41], and ShortStack [42], predicted 46, 173, 175, 27, 139, and 0 candidate miRNA, respectively (Table S4). Just 18% of the candidate miRNAs were predicted by more than one of the programs, among which the predicted structures of candidate miRNAs 0159, 0714, and 1287 contained asymmetrical bulges (Figure 3A).

Because animals, plants, and fungi produce lineage-specific and species-specific miRNAs [10,55,56], the conservation of the candidate miRNA loci of *S*. *sclerotiorum* was analyzed in the family *Sclerotiniaceae*. Among the candidate miRNA loci, just 18.6% were conserved in at least one other member of the family *Sclerotiniaceae*. For example, candidate miRNAs 0386 and 0437 were conserved in the genomes of *Botrytis cinerea* and *Sclerotinia homoeocarpa* (Figure 3B).

### 3.4. Potential Targets for S. sclerotiorum sRNAs

Because in silico prediction of miRNA targets alone can produce a large number of false positives in fungi [57], HT-RACE or degradome was used to identify mRNA with uncapped 5′ termini that could represent cleavage events. The abundance of HT-RACE reads were mapped on *S. sclerotiorum* coding sequences relative to the positions of peak cluster sequences and candidate miRNAs sequences with CleaveLand [47] to detect possible sRNA-mediated slicing sites. CleaveLand separated potential cleavage sites into five categories depending on the number of HT-RACE reads that aligned relative to the sRNA. In category 0 cleavage sites, the sRNA aligned over the most abundantly detected 5′ terminus in the coding sequence. Eighteen candidate miRNAs and 22 sequences from cluster analysis had degradome *P* values of less than 0.05 and category 0 or category 1 slicing sites (Table S5). As has been reported in other systems [58], one of the milRNAs, which was also identified by the cluster analysis, was predicted to direct cleavage of transcripts of SS1G_00334, which is predicted to encode an Argonaute 2 homolog. Other predicted targets included an ammonium transporter (SS1G_04502), an extracellular membrane protein (SS1G_12056), a pachytene checkpoint protein (SS1G_12812) and a retrovirus-related polymerase (SS1G_12103) (Table S5).

### 3.5. Detection of Phased siRNAs from S. sclerotiorum Noncoding RNAs

**Figure 4:**
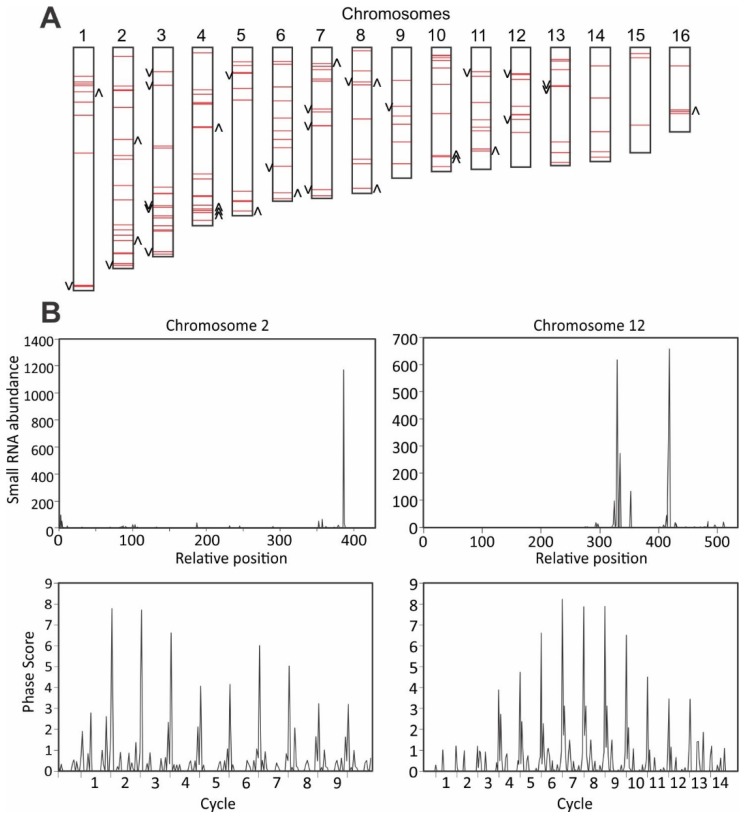
Genome-wide identification of loci producing phased small RNAs in *S*. *sclerotiorum*. (**A**) Distribution of loci producing phased small RNAs (red horizontal bars) on *S*. *sclerotiorum* chromosomes. Loci marked with asterisks showed significantly different accumulation of small RNAs between mock-inoculated and SsHV2L-infected samples. Asterisks on the left of the bars indicate reduced small RNA accumulation; bars to the right indicate increased small RNA accumulation. (**B**) Small RNA abundances and phasing score distributions across two loci producing phased small RNAs in the *S*. *sclerotiorum* genome.

Most (78.8%) of the *S. sclerotiorum* sRNAs were derived from intergenic regions, and de novo assembly of the *S. sclerotiorum* transcriptome data identified 8761 noncoding transcripts. Analysis of the distribution of phasing of sRNA reads in the *S. sclerotiorum* genome identified 164 loci producing 21-nt or 22-nt phasiRNAs with phasing scores greater than 25 (Figure 4A). Thirty-six loci showed significantly different (*p* < 0.05) accumulations of sRNAs between mock-inoculated and SsHV2-L-infected samples (Figure 4A). Seventeen showed reduced accumulation and 19 showed enhanced accumulation of sRNA sequences. Plots of phasing scores of sliding 10-cycle windows showed characteristic repeating patterns for phased loci (Figure 4B). The detection of large numbers of loci producing phasiRNAs suggests that *S. sclerotiorum* produces sRNAs that function similar to miRNAs in higher eukaryotes.

## 4. Discussion

In this study, we analyzed changes in mRNA and sRNA accumulation in *S. sclerotiorum* in response to persistent hypovirus infection and found that infection by SsHV2-L altered the accumulation of nearly 10% of coding mRNAs and sRNAs. The ability to experimentally inoculate *S. sclerotiorum* with SsHV2-L using in vitro transcripts allowed us to fine map virus-induced changes in mRNA and sRNA accumulation during hypovirus infection. Related studies examined changes in gene expression induced by hypovirus infection in *C. parasitica* and *F. graminearum*. In *C. parasitica*, CHV1 infection altered the expression of mRNAs predicted to encode proteins involved in carbon metabolism, stress responses, and regulation of transcription [28], which was supported by metabolomics analyses [59]. Even though FgHV1 does not induce hypovirulence, FgHV1 infection of *F. graminearum* also significantly altered the accumulation of mRNAs predicted to encode proteins involved in sugar and carbohydrate metabolism [60]. Metabolism of mannitol and other sugars are important in compatible fungal–host interactions [61]. Hence, persistent SsHV2-L infections may be metabolically similar to latent viral infections where carbon is directed away from the TCA cycle for glycolysis to reduce apoptosis [62].

Some of the transcripts differentially expressed in this study provided insights on evolutionarily conserved basal defense systems. Infection of *S. sclerotiorum* by SsHV2-L induced expression of mRNAs predicted to encode stress-responsive proteins including a putative mitochondria-localized pentatricopeptide repeat-containing protein and a DEAD-box RNA helicase. In *Arabidopsis*, the stress-induced mitochondria-localized pentatricopeptide repeat protein, PGN, is involved in defense against necrotrophic fungi and tolerance to abiotic stresses [63]. In addition to their deeply conserved roles in stress responses [64], host DEAD-box RNA helicases enhance and sometimes are required for the replication of animal and plant RNA viruses [65,66]. Hence, it is possible that *S. sclerotiorum* DEAD-box RNA helicases could enhance SsHV2-L accumulation.

Infection of *S*. *sclerotiorum* by SsHV2-L did not alter the accumulation of mRNAs encoding RNA silencing-related proteins. In contrast, infection of *C*. *parasitica* by CHV1 upregulated *DCL2* mRNA accumulation up to 20-fold [67]. One of the four *C*. *parasitica* AGO genes, *AGO2*, which is required for antiviral defense, was also upregulated by CHV1 infection [68,69]. Similarly, one DCL, one AGO, and two RDR genes were upregulated in *Rosellinia necatrix* by infection with Rosellinia necatrix mycoreovirus 3 or Rosellinia necatrix megabirnavirus 1 [70]. Similar to infection of *S. sclerotiorum* by SsHV2-L, infection of *R. necatrix* by Rosellinia necatrix partitivirus 1, Rosellinia necatrix quadrivirus 1, and Rosellinia necatrix victorivirus 1 [70] did not significantly alter the expression of AGO, DCL, or RDR homologs [70].

In animals and plants, the presence of conserved core sets of miRNAs helped define the thermodynamic properties for the biogenesis and activity of miRNAs in those systems [10]. However, unlike animals and plants, several recent studies have found little conservation of miRNA genes across fungal taxa [71,72,73,74]. Also, the enzymes involved in RNA silencing are much less conserved in fungi than in higher eukaryotes [75]. For example, neither of the two putative *S*. *sclerotiorum* DCLs (SS1G_13747 and SS1G_10369) contained canonical PAZ domains, which could mean that parameters appropriate for identification of pre-miRNAs in *S*. *sclerotiorum* may differ markedly from those used for plants. As previously reported [76], algorithms selected for identification of *S*. *sclerotiorum* miRNAs and miRNA targets significantly impacted the sequences identified. For example, Zhou et al. [71] described 42 milRNAs in *S*. *sclerotiorum* and confirmed that a subset of the milRNAs was differentially expressed during sclerotial development. However, none of the milRNAs reported by Zhou et al. [71] were identified in the present study. This may be due to differences in a priori assumptions of milRNA predictions; 58.5% of the milRNA sequences reported by Zhou et al. [71] were derived from exons, but coding sequences were excluded from our analyses.

Plants and animals possess ancient and recent, i.e., species-specific, miRNAs [77]. Ancient miRNAs have conserved sequences and targets, while species-specific miRNAs have targets that are more diverse. A small subset of the candidate *S*. *sclerotiorum* miRNAs was conserved in closely related fungal species as reported for oomycetes in the genus *Phytophthora* [56]. The most broadly conserved small RNA sequences were derived from predicted *S*. *sclerotiorum* tRNAs. Small RNAs derived from tRNAs have been shown to regulate gene expression in humans, oomycetes, and plants, and could be important for RNA silencing and gene regulation in *S*. *sclerotiorum* [20,78,79,80]. The detection of phasiRNAs suggests that *S. sclerotiorum* miRNAs are capable of cleaving long noncoding RNAs that act as templates for the production of phasiRNAs to regulate other genes in trans [13].

Infection of *S. sclerotiorum* by SsHV2-L was associated with enhanced production of siRNAs from 437 loci that may be analogous to *Arabidopsis* vasiRNAs that have been associated with widespread silencing of host genes and the establishment of a broad-spectrum antiviral state [26]. In *S. sclerotiorum*, 19 of the loci that showed enhanced sRNA accumulation in SsHV2-L-infected samples produced phasiRNAs. In plants, the production of phasiRNAs is initiated by miRNA cleavage of an RNA template, which functions as a substrate for siRNA production at discrete 21 or 22-nt intervals through the combined action of DCL4 and RDR6 [81]. In *Arabidopsis*, phasiRNAs act as negative regulators of nucleotide-binding, leucine-rich repeat plant defense and other genes that can be triggered by a miRNA [82]. While in *Drosophila*, phasiRNAs are expressed primarily in germline cells and suppress retrotransposons and may serve similar functions in *S. sclerotiorum* [81].

Recent studies revealed that fungi are capable of exporting sRNA that can play an important role in pathogenesis [83] and the host can export small RNA to silence fungal genes involved in pathogenesis [84]. The wheat-infecting fungus *P. striiformis* is predicted to use sRNAs to target resistance gene homologs in wheat [14], which is consistent with findings in *B*. *cinerea* [85] that sRNAs serve as fungal virulence factors. Some *S*. *sclerotiorum* 22-nt sRNAs from the present study were complementary to mRNAs from *B. napus*, *G. max*, and *H. annuus* mRNAs, including mRNAs predicted to encode ferritin-1. In plants, sRNAs of 22-nt can trigger production of secondary siRNAs and rapid silencing of the targeted mRNA [86]. It remains a question whether a subset of the 22-nt RNAs produced by *S. sclerotiorum* are exported to plant hosts and trigger production of secondary siRNA to downregulate host defense genes, such as ferritin-1 in plants.

We previously demonstrated that SsHV2L infection significantly reduced virulence, changed mycelial growth patterns in 3-day old cultures, and delayed and reduced sclerotia production [31]. In a previous study, we observed that most of the SsHV2L-derived vsiRNAs originated from antisense RNA [87]. However in this study, SsHV2L-derived vsiRNAs were produced from both sense and antisense RNAs, which could be related to the different growth conditions used for the two studies. In summary, we predicted novel classes of sRNAs, showed that infection by a mycovirus induced the expression of sRNAs from both coding and noncoding RNAs, and showed that *S*. *sclerotiorum* produced large amounts of tRNA-derived siRNAs and phasiRNAs, the latter of which may be capable of regulating gene expression in trans. The roles of these new classes of fungal sRNAs in gene regulation remain to be determined. Future experiments to dissect fungal RNA silencing pathways by introducing mycoviruses may allow us to assign functions to each putative *S. sclerotiorum* RNA silencing gene. A better understanding of small RNA processing will allow us to use the obtained knowledge to compromise fungal antiviral defenses to better control fungal pathogens. 

## Supplementary Materials

The following are available online at http://www.mdpi.com/1999-4915/10/12/713/s1. Table S1: RNA-Seq analysis of virus-free and hypovirus-transfected *Sclerotinia sclerotiorum*. Table S2: Coding regions differentially expressed between virus-free and hypovirus-infected cultures of *Sclerotinia sclerotiorum*. Table S3: Number of aligning small RNA sequence reads from virus-free and hypovirus-transfected *Sclerotinia sclerotiorum*. Table S4: Candidate microRNA sequence predicted for *Sclerotinia sclerotiorum* small RNA sequence data by give miRNA prediction programs. Table S5: Targets predicted for *Sclerotinia sclerotiorum* small RNAs by degradome sequencing. All the data have been deposited under the NCBI BioProject PRJNA379694.

## References

[1] Ghabrial S.A., Castón J.R., Jiang D., Nibert M.L., Suzuki N. (2015). 50-plus years of fungal viruses. Virology.

[2] Nuss D.L. (2011). Mycoviruses, RNA silencing, and viral RNA recombination. Adv. Virus Res..

[3] Baulcombe D. (2005). RNA silencing. Trends Biochem. Sci..

[4] Waterhouse P.M., Wang M.B., Lough T. (2001). Gene silencing as an adaptive defence against viruses. Nature.

[5] Li Y., Lu J.F., Han Y.H., Fan X.X., Ding S.W. (2013). RNA interference functions as an antiviral immunity mechanism in mammals. Science.

[6] Baulcombe D. (2004). RNA silencing in plants. Nature.

[7] Nakayashiki H., Kadotani N., Mayama S. (2006). Evolution and diversification of RNA silencing proteins in fungi. J. Mol. Evol..

[8] Laurie J.D., Ali S., Linning R., Mannhaupt G., Wong P., Guldener U., Munsterkotter M., Moore R., Kahmann R., Bakkeren G. (2012). Genome comparison of barley and maize smut fungi reveals targeted loss of RNA silencing components and species-specific presence of transposable elements. Plant Cell.

[9] Bernstein D.A., Vyas V.K., Weinberg D.E., Drinnenberg I.A., Bartel D.P., Fink G.R. (2012). *Candida albicans* Dicer (CaDcr1) is required for efficient ribosomal and spliceosomal RNA maturation. Proc. Natl. Acad. Sci. USA.

[10] Axtell M.J., Westholm J.O., Lai E.C. (2011). Vive la différence: Biogenesis and evolution of microRNAs in plants and animals. Genome Biol..

[11] Song X.W., Li P.C., Zhai J.X., Zhou M., Ma L.J., Liu B., Jeong D.H., Nakano M., Cao S.Y., Liu C.Y. (2012). Roles of DCL4 and DCL3b in rice phased small RNA biogenesis. Plant J..

[12] Arikit S., Zhai J.X., Meyers B.C. (2013). Biogenesis and function of rice small RNAs from non-coding RNA precursors. Curr. Opin. Plant Biol..

[13] Allen E., Xie Z.X., Gustafson A.M., Carrington J.C. (2005). MicroRNA-directed phasing during trans-acting siRNA biogenesis in plants. Cell.

[14] Mueth N.A., Ramachandran S.R., Hulbert S.H. (2015). Small RNAs from the wheat stripe rust fungus (*Puccinia striiformis* f.sp. *tritici*). BMC Genom..

[15] Lin R.M., He L.Y., He J.Y., Qin P.G., Wang Y.R., Deng Q.M., Yang X.T., Li S.C., Wang S.Q., Wang W.M. (2016). Comprehensive analysis of microRNA-Seq and target mRNAs of rice sheath blight pathogen provides new insights into pathogenic regulatory mechanisms. DNA Res..

[16] Chen R., Jiang N., Jiang Q.Y., Sun X.J., Wang Y., Zhang H., Hu Z. (2014). Exploring microRNA-like small RNAs in the filamentous fungus *Fusarium oxysporum*. PLoS ONE.

[17] Bai Y.H., Lan F.X., Yang W.Q., Zhang F., Yang K.L., Li Z.G., Gao P.L., Wang S.H. (2015). SRNA profiling in *Aspergillus flavus* reveals differentially expressed miRNA-like RNAs response to water activity and temperature. Fungal Genet. Biol..

[18] Thompson D.M., Parker R. (2009). Stressing out over tRNA cleavage. Cell.

[19] Chen Q., Yan M.H., Cao Z.H., Li X., Zhang Y.F., Shi J.C., Feng G.H., Peng H.Y., Zhang X.D., Zhang Y. (2016). Sperm tsRNAs contribute to intergenerational inheritance of an acquired metabolic disorder. Science.

[20] Martinez G., Choudury S.G., Slotkin R.K. (2017). tRNA-derived small RNAs target transposable element transcripts. Nucleic Acids Res..

[21] Nunes C.C., Gowda M., Sailsbery J., Xue M., Chen F., Brown D.E., Oh Y., Mitchell T.K., Dean R.A. (2011). Diverse and tissue-enriched small RNAs in the plant pathogenic fungus, Magnaporthe oryzae. BMC Genom..

[22] Wang M.B., Bian X.Y., Wu L.M., Liu L.X., Smith N.A., Isenegger D., Wu R.M., Masuta C., Vance V.B., Watson J.M. (2004). On the role of RNA silencing in the pathogenicity and evolution of viroids and viral satellites. Proc. Natl. Acad. Sci. USA.

[23] Qi X., Bao F.S., Xie Z. (2009). Small RNA deep sequencing reveals role for *arabidopsis thaliana* RNA-dependent RNA polymerases in viral siRNA biogenesis. PLoS ONE.

[24] Shimura H., Pantaleo V., Ishihara T., Myojo N., Inaba J., Sueda K., Burgyan J., Masuta C. (2011). A viral satellite RNA induces yellow symptoms on tobacco by targeting a gene involved in chlorophyll biosynthesis using the RNA silencing machinery. PLoS Pathog..

[25] Smith N.A., Eamens A.L., Wang M.B. (2011). Viral small interfering RNAs target host genes to mediate disease symptoms in plants. PLoS Pathog..

[26] Cao M.J., Du P., Wang X.B., Yu Y.Q., Qiu Y.H., Li W.X., Gal-On A., Zhou C.Y., Li Y., Ding S.W. (2014). Virus infection triggers widespread silencing of host genes by a distinct class of endogenous siRNAs in *Arabidopsis*. Proc. Natl. Acad. Sci. USA.

[27] McBride R.C., Boucher N., Park D.S., Turner P.E., Townsend J.P. (2013). Yeast response to la virus indicates coadapted global gene expression during mycoviral infection. FEMS Yeast Res..

[28] Allen T.D., Dawe A.L., Nuss D.L. (2003). Use of cdna microarrays to monitor transcriptional responses of the chestnut blight fungus *cryphonectria parasitica* to infection by virulence-attenuating hypoviruses. Eukaryot. Cell.

[29] Wang J.Z., Shi L.M., He X.P., Lu L.D., Li X.P., Chen B.S. (2016). Comparative secretome analysis reveals perturbation of host secretion pathways by a hypovirus. Sci. Rep..

[30] Kwon S.J., Cho S.Y., Lee K.M., Yu J., Son M., Kim K.H. (2009). Proteomic analysis of fungal host factors differentially expressed by *Fusarium graminearum* infected with fusarium graminearum virus-DK21. Virus Res..

[31] Marzano S.L., Hobbs H.A., Nelson B.D., Hartman G.L., Eastburn D.E., McCoppin N.K., Domier L.L. (2015). Transfection of *Sclerotinia sclerotiorum* with *in vitro* transcripts of a naturally occurring interspecific recombinant of Sclerotinia sclerotiorum hypovirus 2 significantly reduces virulence of the fungus. J. Virol..

[32] Grabherr M.G., Haas B.J., Yassour M., Levin J.Z., Thompson D.A., Amit I., Adiconis X., Fan L., Raychowdhury R., Zeng Q. (2011). Full-length transcriptome assembly from RNA-Seq data without a reference genome. Nat. Biotechnol..

[33] Hoff K.J., Lange S., Lomsadze A., Borodovsky M., Stanke M. (2016). Braker1: Unsupervised RNA-Seq-based genome annotation with GeneMark-ET and AUGUSTUS. Bioinformatics.

[34] Li B., Dewey C.N. (2011). Rsem: Accurate transcript quantification from RNA-Seq data with or without a reference genome. BMC Bioinform..

[35] Love M.I., Huber W., Anders S. (2014). Moderated estimation of fold change and dispersion for RNA-Seq data with DESeq2. Genome Biol..

[36] Mi H.Y., Muruganujan A., Casagrande J.T., Thomas P.D. (2013). Large-scale gene function analysis with the PANTHER classification system. Nat. Protoc..

[37] Mortazavi A., Williams B.A., Mccue K., Schaeffer L., Wold B. (2008). Mapping and quantifying mammalian transcriptomes by RNA-Seq. Nat. Methods.

[38] Mackowiak S.D. (2011). Identification of novel and known miRNAs in deep-sequencing data with miRDeep2. Curr. Protoc. Bioinform..

[39] An J.Y., Lai J., Lehman M.L., Nelson C.C. (2013). miRDeep*: An integrated application tool for miRNA identification from RNA sequencing data. Nucleic Acids Res..

[40] Jones-Rhoades M.W. (2010). Prediction of plant miRNA genes. Plant MicroRNAs: Methods and Protocols.

[41] An J.Y., Lai J., Sajjanhar A., Lehman M.L., Nelson C.C. (2014). miRPlant: An integrated tool for identification of plant miRNA from RNA sequencing data. BMC Bioinform..

[42] Axtell M.J. (2013). Shortstack: Comprehensive annotation and quantification of small RNA genes. RNA.

[43] Johnson N.R., Yeoh J.M., Coruh C., Axtell M.J. (2016). Improved placement of multi-mapping small RNAs. G3.

[44] Howell M.D., Fahlgren N., Chapman E.J., Cumbie J.S., Sullivan C.M., Givan S.A., Kasschau K.D., Carrington J.C. (2007). Genome-wide analysis of the RNA-DEPENDENT RNA POLYMERASE6/DICER-LIKE4 pathway in *Arabidopsis* reveals dependency on miRNA- and tasiRNA-directed targeting. Plant Cell.

[45] Lowe T.M., Chan P.P. (2016). tRNAscan-SE on-line: Integrating search and context for analysis of transfer RNA genes. Nucleic Acids Res..

[46] Li F., Baker B. (2012). Preparation of cDNA Library for dRNA-Seq. Bio-protocol.

[47] Addo-Quaye C., Miller W., Axtell M.J. (2009). Cleaveland: A pipeline for using degradome data to find cleaved small RNA targets. Bioinformatics.

[48] Amselem J., Cuomo C.A., van Kan J.A.L., Viaud M., Benito E.P., Couloux A., Coutinho P.M., de Vries R.P., Dyer P.S., Fillinger S. (2011). Genomic analysis of the necrotrophic fungal pathogens *Sclerotinia sclerotiorum* and *Botrytis cinerea*. PLoS Genet..

[49] Whitham S.A., Yang C.L., Goodin M.M. (2006). Global impact: Elucidating plant responses to viral infection. Mol. Plant-Microbe Interact..

[50] Spain B.H., Koo D., Ramakrishnan M., Dzudzor B., Colicelli J. (1995). Truncated forms of a novel yeast protein suppress the lethality of a G-protein α subunit deficiency by interacting with the β subunit. J. Biol. Chem..

[51] Wei W. (2017). Transcriptomic Characterization of Soybean–Sclerotinia Sclerotiorum Interaction at Early Infection Stages. Ph.D. Thesis.

[52] Fei Q., Yu Y., Liu L., Zhang Y., Baldrich P., Dai Q., Chen X., Meyers B.C. (2018). Biogenesis of a 22-nt microRNA in Phaseoleae species by precursor-programmed uridylation. Proc. Natl. Acad. Sci. USA.

[53] Hammond T.M., Spollen W.G., Decker L.M., Blake S.M., Springer G.K., Shiu P.K.T. (2013). Identification of small RNAs associated with meiotic silencing by unpaired DNA. Genetics.

[54] Xu Z., Huang G., Song N., Wang J., Cao L., Jiang H., Ding T. (2016). Complete mitochondrial genome sequence of the phytopathogenic fungi *Sclerotinia sclerotiorum* JX-21. Mitochondrial DNA Part B.

[55] Zhu R.S., Li X., Chen Q.S. (2011). Discovering numerical laws of plant microRNA by evolution. Biochem. Biophys. Res. Commun..

[56] Fahlgren N., Bollmann S.R., Kasschau K.D., Cuperus J.T., Press C.M., Sullivan C.M., Chapman E.J., Hoyer J.S., Gilbert K.B., Grunwald N.J. (2013). *Phytophthora* have distinct endogenous small RNA populations that include short interfering and microRNAs. PLoS ONE.

[57] Pinzon N., Li B., Martinez L., Sergeeva A., Presumey J., Apparailly F., Seitz H. (2017). microRNA target prediction programs predict many false positives. Genome Res..

[58] Vaucheret H., Vazquez F., Crete P., Bartel D.P. (2004). The action of argonaute1 in the miRNA pathway and its regulation by the miRNA pathway are crucial for plant development. Gene Dev..

[59] Dawe A.L., Van Voorhies W.A., Lau T.A., Ulanov A.V., Li Z. (2009). Major impacts on the primary metabolism of the plant pathogen *Cryphonectria parasitica* by the virulence-attenuating virus CHV1-EP713. Microbiology.

[60] Wang S.C., Zhang J.Z., Li P.F., Qiu D.W., Guo L.H. (2016). Transcriptome-based discovery of *Fusarium graminearum* stress responses to FgHV1 infection. Int. J. Mol. Sci..

[61] Meena M., Prasad V., Zehra A., Gupta V.K., Upadhyay R.S. (2015). Mannitol metabolism during pathogenic fungal-host interactions under stressed conditions. Front. Microbiol..

[62] Delgado T., Carroll P.A., Punjabi A.S., Margineantu D., Hockenbery D.M., Lagunoff M. (2010). Induction of the Warburg effect by Kaposi’s sarcoma herpesvirus is required for the maintenance of latently infected endothelial cells. Proc. Natl. Acad. Sci. USA.

[63] Laluk K., AbuQamar S., Mengiste T. (2011). The Arabidopsis mitochondria-localized pentatricopeptide repeat protein PGN functions in defense against necrotrophic fungi and abiotic stress tolerance. Plant Physiol..

[64] Zhu M.K., Chen G.P., Dong T.T., Wang L.L., Zhang J.L., Zhao Z.P., Hu Z.L. (2015). *SLDEAD31*, a putative DEAD-box RNA helicase gene, regulates salt and drought tolerance and stress-related genes in tomato. PLoS ONE.

[65] Li C., Ge L.L., Li P.P., Wang Y., Sun M.X., Huang L., Ishag H., Di D.D., Shen Z.Q., Fan W.X. (2013). The DEAD-box RNA helicase DDX5 acts as a positive regulator of Japanese encephalitis virus replication by binding to viral 3′ UTR. Antivir. Res..

[66] Kovalev N., Pogany J., Nagy P.D. (2012). A co-opted dead-box RNA helicase enhances tombusvirus plus-strand synthesis. PLoS Pathog..

[67] Chiba S., Suzuki N. (2015). Highly activated RNA silencing via strong induction of dicer by one virus can interfere with the replication of an unrelated virus. Proc. Natl. Acad. Sci. USA.

[68] Zhang X.M., Segers G.C., Sun Q.H., Deng F.Y., Nuss D.L. (2008). Characterization of hypovirus-derived small RNAs generated in the chestnut blight fungus by an inducible DCL-2-dependent pathway. J. Virol..

[69] Sun Q., Choi G.H., Nuss D.L. (2009). A single argonaute gene is required for induction of RNA silencing antiviral defense and promotes viral RNA recombination. Proc. Natl. Acad. Sci. USA.

[70] Yaegashi H., Shimizu T., Ito T., Kanematsu S. (2016). Differential inductions of RNA silencing among encapsidated double-stranded RNA mycoviruses in the white root rot fungus *Rosellinia necatrix*. J. Virol..

[71] Zhou J.H., Fu Y.P., Xie J.T., Li B., Jiang D.H., Li G.Q., Cheng J.S. (2012). Identification of microRNA-like RNAs in a plant pathogenic fungus *Sclerotinia sclerotiorum* by high-throughput sequencing. Mol. Genet. Genom..

[72] Lau S.K.P., Chow W.N., Wong A.Y.P., Yeung J.M.Y., Bao J., Zhang N., Lok S., Woo P.C.Y., Yuen K.Y. (2013). Identification of microRNA-like RNAs in mycelial and yeast phases of the thermal dimorphic fungus *Penicillium marneffei*. PLoS Negl. Trop. Dis..

[73] Kang K., Zhong J.S., Jiang L., Liu G., Gou C.Y., Wu Q., Wang Y., Luo J., Gou D.M. (2013). Identification of microRNA-like RNAs in the filamentous fungus *Trichoderma reesei* by Solexa sequencing. PLoS ONE.

[74] Dahlmann T.A., Kuck U. (2015). Dicer-dependent biogenesis of small RNAs and evidence for microRNA-like RNAs in the penicillin producing fungus *Penicillium chrysogenum*. PLoS ONE.

[75] Chang S.S., Zhang Z.Y., Liu Y. (2012). RNA interference pathways in fungi: Mechanisms and functions. Annu. Rev. Microbiol..

[76] Williamson V., Kim A., Xie B., McMichael G.O., Gao Y., Vladimirov V. (2013). Detecting miRNAs in deep-sequencing data: A software performance comparison and evaluation. Brief. Bioinform..

[77] Waterhouse P.M., Hellens R.P. (2015). Coding in non-coding RNAs. Nature.

[78] Wang Q.H., Li T.T., Xu K., Zhang W., Wang X.L., Quan J.L., Jin W.B., Zhang M.X., Fan G.J., Wang M.B. (2016). The tRNA-derived small RNAs regulate gene expression through triggering sequence-specific degradation of target transcripts in the oomycete pathogen *Phytophthora sojae*. Front. Plant Sci..

[79] Haussecker D., Huang Y., Lau A., Parameswaran P., Fire A.Z., Kay M.A. (2010). Human tRNA-derived small RNAs in the global regulation of RNA silencing. RNA.

[80] Keam S.P., Hutvagner G. (2015). tRNA-derived fragments (tRFs): Emerging new roles for an ancient RNA in the regulation of gene expression. Life.

[81] Komiya R. (2017). Biogenesis of diverse plant phasiRNAs involves an miRNA-trigger and Dicer-processing. J. Plant Res..

[82] Fei Q.L., Xia R., Meyers B.C. (2013). Phased, secondary, small interfering RNAs in posttranscriptional regulatory networks. Plant Cell.

[83] Da Silva R.P., Puccia R., Rodrigues M.L., Oliveira D.L., Joffe L.S., Cesar G.V., Nimrichter L., Goldenberg S., Alves L.R. (2015). Extracellular vesicle-mediated export of fungal RNA. Sci. Rep..

[84] Cai Q., Qiao L., Wang M., He B., Lin F.-M., Palmquist J., Huang H.-D., Jin H. (2018). Plants send small RNAs in extracellular vesicles to fungal pathogen to silence virulence genes. Science.

[85] Weiberg A., Wang M., Lin F.M., Zhao H.W., Zhang Z.H., Kaloshian I., Huang H.D., Jin H.L. (2013). Fungal small RNAs suppress plant immunity by hijacking host RNA interference pathways. Science.

[86] Chen H.M., Chen L.T., Patel K., Li Y.H., Baulcombe D.C., Wu S.H. (2010). 22-nucleotide RNAs trigger secondary siRNA biogenesis in plants. Proc. Natl. Acad. Sci. USA.

[87] Mochama P., Jadhav P., Neupane A., Marzano S.-Y.L. (2018). Mycoviruses as Triggers and Targets of RNA Silencing in White Mold Fungus Sclerotinia sclerotiorum. Viruses.

