# Supplementary material for: Transcriptional and Small RNA Responses of the White Mold Fungus Sclerotinia sclerotiorum to Infection by a Virulence-Attenuating Hypovirus
Source: Viruses. 2018 Dec 14;10(12):713. doi: 10.3390/v10120713 (PMC6315951; doi:10.3390/v10120713)
Supplement: Supplementary file 1 [file viruses-10-00713-s001.pdf]

Table S1a. Trial #1 RNA-Seq analysis of virus-free and hypovirus-transfected *Sclerotinia sclerotiorum*

	Library									
	Virus-free_1		Virus-free_2		Virus-infected_1		Virus-infected_2		Total	
	Count	%	Count	%	Count	%	Count	%	Count	%
Raw reads	48,854,170	100.0%	56,568,277	100.0%	48,169,458	100.0%	57,396,928	100.0%	210,988,833	100.0%
Filtered reads	48,810,008	99.9%	56,518,010	99.9%	48,118,009	99.9%	57,341,604	99.9%	210,787,631	99.9%
S. sclerotiorum genome										
Total aligned	44,085,026	90.3%	50,724,764	89.8%	41,374,916	86.0%	50,580,960	88.2%	186,765,666	88.6%
Unaligned	4,724,982	9.7%	5,793,246	10.3%	6,743,093	14.0%	6,760,644	11.8%	24,021,965	11.5%
Intergenic regions (non-rRNA)	13,918,285	28.5%	16,317,039	28.9%	9,354,071	19.4%	15,230,421	26.6%	54,819,816	25.8%
Ribosomal RNA	9,150,409	18.7%	6,442,905	11.4%	3,329,107	6.8%	5,976,737	10.4%	24,899,158	11.8%
Retrotransposons	202,264	0.4%	268,505	0.5%	251,134	0.5%	499,377	0.9%	1,221,280	0.6%
Coding sequences										
Aligned	28,057,390	57.5%	36,310,362	64.2%	29,370,507	61.0%	37,649,843	65.7%	131,388,102	62.1%
plus strand	27,285,917	97.3%	35,481,217	97.7%	28,409,529	96.7%	36,766,211	97.7%	127,942,874	97.3%
minus strand	771,473	2.7%	829,145	2.3%	960,978	3.3%	883,632	2.3%	3,445,228	2.7%
Unaligned	20,752,618	42.5%	20,207,648	35.8%	18,747,502	39.0%	19,691,761	34.3%	79,399,529	37.9%
Sclerotinia sclerotiorum hypovirus 2L (SsHV2L)										
Aligned	62*	-	77	-	1,165,087	2.4%	216,286	0.4%	1,381,450	1.4%
plus strand	-	-	-	-	1,150,841	98.8%	210,533	97.3%	1,361,374	98.1%
minus strand	-	-	-	-	14,246	1.2%	5,753	2.7%	19,999	1.9%

*SsHV2L reads in non-inoculated samples likely result from errors in barcode sequences.

Table S1b. Trial #2 RNA-Seq analysis of virus-free and hypovirus-transfected *Sclerotinia sclerotiorum*

	Library											
	Virus-free_1		Virus-free_2		Virus-free_3		Virus-free_4		Virus-free_5		Total	
	Count	%	Count	%	Count	%	Count	%	Count	%	Count	%
Raw reads	30,093,145	100.0%	40,579,363	100.0%	38,997,051	100.0%	44,941,314	100.0%	33,080,107	100.0%	187,690,980	100.0%
Filtered reads	30,042,994	99.8%	40,505,260	99.8%	38,927,531	99.8%	44,846,033	99.8%	33,015,439	99.8%	187,337,257	99.8%
S. sclerotiorum genome												
Total aligned	26,871,479	89.4%	35,782,013	88.3%	34,813,856	89.4%	39,621,053	88.3%	29,615,431	89.7%	166,703,832	89.0%
Unaligned	3,171,515	10.6%	4,723,247	11.7%	4,113,675	10.6%	5,224,980	11.7%	3,400,008	10.3%	20,633,425	11.0%
Intergenic regions (non-rRNA)	4,998,186	16.6%	7,686,048	19.0%	6,434,536	16.5%	7,752,858	17.3%	5,060,561	15.3%	31,932,189	17.0%
Ribosomal RNA	3,683,044	12.3%	2,805,329	6.9%	4,539,626	11.7%	3,322,822	7.4%	6,155,590	18.6%	20,506,411	10.9%
Retrotransposons	114,957	0.4%	126,391	0.3%	136,418	0.4%	130,049	0.3%	88,464	0.3%	596,279	0.3%
Coding sequences												
Aligned	17,278,688	57.5%	23,840,323	58.9%	22,638,558	58.2%	27,223,916	60.7%	17,511,758	53.0%	108,493,243	57.9%
plus strand	17,166,363	99.3%	23,625,424	99.1%	22,486,446	99.3%	27,025,184	99.3%	17,385,842	99.3%	107,689,259	99.3%
minus strand	112,325	0.7%	214,899	0.9%	152,112	0.7%	198,732	0.7%	125,916	0.7%	803,984	0.7%
Unaligned	12,764,306	42.5%	16,664,937	41.1%	16,288,973	41.8%	17,622,117	39.3%	15,503,681	47.0%	78,844,014	42.1%
Sclerotinia sclerotiorum hypovirus 2L (SsHV2L)												
Aligned	36*	0.0%	511	0.0%	380	0.0%	195	0.0%	23	0.0%	1,145	0.0%
plus strand	35	0.0%	499	0.0%	373	0.0%	192	0.0%	23	0.0%	1,122	0.0%
minus strand	1	0.0%	12	0.0%	7	0.0%	3	0.0%	0	0.0%	23	0.0%

Table S1b (continue). Trial #2 RNA-Seq analysis of virus-free and hypovirus-transfected *Sclerotinia sclerotiorum*

	Virus-infected_1		Virus-infected_2		Virus-infected_3		Virus-infected_4		Virus-infected_5		Total	
	Count	%	Count	%	Count	%	Count	%	Count	%	Count	%
Raw reads	43,688,888	100.0%	39,373,313	100.0%	43,420,192	100.0%	39,112,539	100.0%	45,777,312	100.0%	211,372,244	100.0%
Filtered reads	43,609,413	99.8%	39,295,735	99.8%	43,337,442	99.8%	39,047,576	99.8%	45,689,530	99.8%	210,979,696	99.8%
S. sclerotiorum genome												
Total aligned	35,507,122	81.4%	32,573,106	82.9%	37,001,902	85.4%	34,039,626	87.2%	37,539,733	82.2%	176,661,489	83.7%
Unaligned	8,102,291	18.6%	6,722,629	17.1%	6,335,540	14.6%	5,007,950	12.8%	8,149,797	17.8%	34,318,207	16.3%
Intergenic regions (non-rRNA)	7,045,401	16.2%	7,590,429	19.3%	8,674,711	20.0%	7,894,153	20.2%	7,714,740	16.9%	38,919,434	18.4%
Ribosomal RNA	2,686,202	6.2%	892,650	2.3%	606,690	1.4%	885,034	2.3%	4,733,875	10.4%	9,804,451	4.6%
Retrotransposons	157,132	0.4%	163,611	0.4%	175,054	0.4%	114,497	0.3%	156,372	0.3%	766,666	0.4%
Coding sequences												
Aligned	24,495,352	56.2%	22,530,142	57.3%	26,019,200	60.0%	23,647,091	60.6%	23,602,390	51.7%	120,294,175	57.0%
plus strand	24,288,375	99.2%	22,329,958	99.1%	25,782,683	99.1%	23,437,236	99.1%	23,412,311	99.2%	119,250,563	99.1%
minus strand	206,977	0.8%	200,184	0.9%	236,517	0.9%	209,855	0.9%	190,079	0.8%	1,043,612	0.9%
Unaligned	19,114,061	43.8%	16,765,593	42.7%	17,318,242	40.0%	15,400,485	39.4%	22,087,140	48.3%	90,685,521	43.0%
Sclerotinia sclerotiorum hypovirus 2L (SsHV2L)												
Aligned	3,546,501	8.1%	2,587,540	6.6%	1,650,572	3.8%	782,150	2.0%	3,537,497	7.7%	12,104,260	5.7%
plus strand	3,422,612	96.5%	2,499,769	96.6%	1,610,062	97.5%	769,161	98.3%	3,425,008	96.8%	11,726,612	96.9%
minus strand	123,889	3.5%	87,771	3.4%	40,510	2.5%	12,989	1.7%	112,489	3.2%	377,648	3.1%

*SsHV2L reads in non-inoculated samples likely result from errors in barcode sequences.

Table S2a. Trial #1 coding regions differentially expressed between virus-free and hypovirus-infected cultures of *Sclerotinia sclerotiorum*

		RNASeq		
**Locus**	**Annotation**	**Log Fold Change**	**adjusted *P* value**	**Up/Down**
SS1G_11521	Hypothetical protein	7.96	1.29E-70	Up
SS1G_08066	Hypothetical protein	7.94	2.20E-79	Up
SS1G_08042	Protein kinase	7.55	2.22E-29	Up
SS1G_14214	Hypothetical protein	7.20	1.43E-59	Up
SS1G_11520	Hypothetical protein	7.04	2.36E-46	Up
SS1G_11519	Heat shock protein 70	6.58	1.92E-59	Up
SS1G_03997	Splicing factor	6.55	5.32E-34	Up
SS1G_13734	Carbohydrate transporter	6.48	3.03E-46	Up
SS1G_08001	Hypothetical protein	6.40	9.69E-21	Up
SS1G_08063	Succinyltransferase	6.19	5.26E-54	Up
SS1G_04312	Endochitinase	6.09	1.28E-18	Up
SS1G_14293	Glucose oxidase	5.96	2.31E-35	Up
SS1G_04857	Endopolygalacturonase	5.86	1.33E-43	Up
SS1G_08041	DUF3723 domain-containing protein	5.76	1.76E-15	Up
SS1G_12509	LysM domain-containing protein	5.63	1.39E-28	Up
SS1G_09723	Hypothetical protein	5.40	1.44E-18	Up
SS1G_05933	Hypothetical protein	5.33	4.76E-15	Up
SS1G_12142	Sugar transport protein	5.29	4.25E-28	Up
SS1G_04841	Sshxt1 sugar transport protein	5.25	3.72E-21	Up
SS1G_04353	Hypothetical protein	5.17	1.18E-31	Up
SS1G_10617	Glucoamylase	5.10	7.86E-44	Up
SS1G_08062	Hypothetical protein	5.02	7.44E-30	Up
SS1G_04305	Dehydrogenase	5.02	3.01E-21	Up
SS1G_01009	Polygalacturonase	4.99	1.24E-14	Up
SS1G_12425	non-LTR retrotransposon	4.98	6.06E-13	Up
SS1G_00755	Hypothetical protein	4.92	3.91E-17	Up
SS1G_11912	Hypothetical protein	4.83	2.57E-24	Up
SS1G_01235	Hypothetical protein	4.71	1.08E-13	Up
SS1G_05860	Hypothetical protein	4.71	1.59E-12	Up
SS1G_12510	Endochitinase	4.70	1.20E-17	Up
SS1G_04331	Hypothetical protein	4.66	1.10E-17	Up
SS1G_00750	Hypothetical protein	4.55	3.49E-14	Up
SS1G_09722	Serine protease	4.51	8.18E-34	Up
SS1G_09518	Hypothetical protein	4.49	4.56E-34	Up
SS1G_02383	ABC transporter G family member	4.42	9.44E-27	Up
SS1G_07224	Hypothetical protein	4.41	3.42E-12	Up
SS1G_13687	Hypothetical protein	4.27	8.63E-13	Up
SS1G_13860	Endoglucanase	4.25	1.21E-22	Up
SS1G_07184	Putative invertase	4.23	8.63E-28	Up
SS1G_02382	ABC transporter G family member	4.16	2.36E-17	Up
SS1G_14244	Probable efflux pump protein	4.15	1.02E-14	Up

SS1G_04465	Dehydrogenase/reductase	4.14	8.49E-09	Up
SS1G_08040	Serine/threonine-protein kinase	4.10	9.74E-06	Up
SS1G_02454	Aflatoxin B1 aldehyde reductase member	4.08	8.22E-13	Up
SS1G_08051	Hypothetical protein	4.07	6.60E-23	Up
SS1G_13356	Hypothetical protein	4.05	1.83E-20	Up
SS1G_06707	Hypothetical protein	4.02	1.05E-06	Up
SS1G_04292	Putative alpha-ketoglutarate-dependent sulfonate dioxygenase	3.98	9.67E-10	Up
SS1G_04030	Lysophospholipase 2	3.97	6.37E-12	Up
SS1G_03092	Sugar transport protein	3.96	8.65E-18	Up
SS1G_04339	Protein FDD123	3.95	4.85E-08	Up
SS1G_01990	Hypothetical protein	3.85	3.98E-08	Up
SS1G_13359	Methyltransferase trt5	3.84	4.06E-14	Up
SS1G_11753	Aflatoxin B1 aldehyde reductase	3.79	2.33E-07	Up
SS1G_00148	Versiconal hemiacetal acetate reductase	3.79	2.05E-10	Up
SS1G_05550	Hypothetical protein	3.78	1.74E-08	Up
SS1G_04279	Hypothetical protein	3.75	5.44E-08	Up
SS1G_08545	Hypothetical protein	3.75	1.43E-09	Up
SS1G_09392	Glucoamylase	3.74	4.16E-17	Up
SS1G_09624	Hypotentical protein	3.73	5.59E-16	Up
SS1G_09517	Chaperone protein DnaK	3.70	2.02E-16	Up
SS1G_13362	Amidohydrolase	3.70	6.16E-08	Up
SS1G_09350	Hypothetical protein	3.67	9.98E-16	Up
SS1G_01776	Alpha-amylase	3.65	2.06E-12	Up
SS1G_12917	WSC domain-containing glycosyltransferase	3.63	1.44E-10	Up
SS1G_13872	Probable endo-1,3(4)-beta-glucanase	3.58	1.86E-06	Up
SS1G_13003	Hypothetical protein	3.54	4.28E-11	Up
SS1G_05338	Hypothetical protein	3.53	3.82E-04	Up
SS1G_13358	Hypothetical protein	3.53	2.68E-16	Up
SS1G_08000	Hypothetical protein	3.50	4.43E-04	Up
SS1G_00727	Aldo-keto reductase	3.50	1.51E-13	Up
SS1G_13355	Phenylalanine aminomutase	3.48	2.68E-16	Up
SS1G_13686	pH-response regulator protein	3.44	6.64E-11	Up
SS1G_11988	Glucose dehydrogenase	3.43	3.52E-15	Up
SS1G_08007	Hypothetical protein	3.43	1.61E-04	Up
SS1G_10071	Probable pectin lyase A	3.40	5.66E-06	Up
SS1G_06664	Purine-cytosine permease FCY21	3.40	1.28E-16	Up
SS1G_11914	Hypothetical protein	3.40	4.75E-08	Up
SS1G_02455	Acetylornithine aminotransferase	3.33	1.54E-15	Up
SS1G_02941	Hypothetical protein	3.33	5.26E-04	Up
SS1G_06448	Retinol dehydrogenase	3.33	1.98E-07	Up
SS1G_02194	Oxygenase	3.33	1.10E-08	Up
SS1G_00756	Hypothetical protein	3.33	5.31E-06	Up
SS1G_00757	Hypothetical protein	3.32	2.46E-11	Up
SS1G_06091	Hypothetical protein	3.32	2.68E-07	Up
SS1G_05787	Non-reducing polyketide synthase	3.32	2.52E-07	Up
SS1G_06132	Dehydrogenase	3.32	3.40E-15	Up

SS1G_05360	Hypothetical protein	3.31	5.56E-08	Up
SS1G_05430	Hypothetical protein	3.31	5.03E-09	Up
SS1G_04090	ABC transporter	3.29	2.01E-06	Up
SS1G_09449	Non-canonical non-ribosomal peptide synthetase	3.29	2.68E-09	Up
SS1G_09834	Hypothetical protein	3.29	4.34E-11	Up
SS1G_08028	Hypothetical protein	3.28	3.85E-04	Up
SS1G_09771	Dehydrogenase/reductase	3.28	7.50E-07	Up
SS1G_01790	Zip1-like transcription factor	3.27	7.94E-10	Up
SS1G_04614	Oxidoreductase	3.26	5.29E-08	Up
SS1G_06068	Hypothetical protein	3.25	1.28E-06	Up
SS1G_04088	Hydroxylase/oxygenase	3.24	1.73E-11	Up
SS1G_11524	Hypothetical protein	3.23	1.17E-15	Up
SS1G_12778	Hypothetical protein	3.23	1.32E-15	Up
SS1G_09374	Oxidoreductase	3.22	2.40E-10	Up
SS1G_11769	Hypothetical protein	3.20	2.31E-11	Up
SS1G_02676	Sexual differentiation process protein	3.19	3.24E-14	Up
SS1G_11773	Hypothetical protein	3.18	6.36E-10	Up
SS1G_11513	Hypothetical protein	3.16	1.25E-10	Up
SS1G_05507	Hypothetical protein	3.16	8.01E-11	Up
SS1G_05565	Hypothetical protein	3.15	1.63E-03	Up
SS1G_01231	Probable sulfate permease	3.15	4.12E-07	Up
SS1G_01166	Vanillin dehydrogenase	3.14	9.76E-12	Up
SS1G_14460	1,3-beta-glucanosyltransferase	3.12	1.99E-10	Up
SS1G_13851	Hypothetical protein	3.11	2.17E-07	Up
SS1G_04372	Uncharacterized transporter	3.10	1.47E-11	Up
SS1G_00872	Hypothetical protein	3.10	1.15E-13	Up
SS1G_05337	Hypothetical protein	3.08	2.52E-10	Up
SS1G_08746	Hypothetical protein	3.07	1.97E-06	Up
SS1G_13233	Hypothetical protein	3.07	2.82E-13	Up
SS1G_12116	Didemethylasterriquinone D synthetase	3.06	1.04E-06	Up
SS1G_06921	Hypothetical protein	3.06	1.56E-06	Up
SS1G_12907	Cutinase	3.05	2.14E-07	Up
SS1G_04089	Hypothetical protein	3.05	2.74E-08	Up
SS1G_08103	Fumarate reductase flavoprotein subunit	3.04	4.00E-06	Up
SS1G_10222	Hypothetical protein	3.02	6.13E-12	Up
SS1G_13360	Hypothetical protein	3.01	2.43E-08	Up
SS1G_08643	Hypothetical protein	3.00	3.93E-07	Up
SS1G_02834	Hypothetical protein	2.99	6.59E-06	Up
SS1G_01939	Hypothetical protein	2.99	3.56E-03	Up
SS1G_01167	Transporter	2.98	4.01E-13	Up
SS1G_00868	Hypothetical protein	2.95	4.67E-05	Up
SS1G_08043	Hypothetical protein	2.95	9.92E-10	Up
SS1G_08148	Eukaryotic translation initiation factor 1b	2.94	1.60E-03	Up
SS1G_04322	DEAD-box ATP-dependent RNA helicase	2.94	2.49E-08	Up
SS1G_06365	Dihydrogeodin oxidase	2.94	5.89E-12	Up
SS1G_13850	Cyclochlorotine biosynthesis protein	2.93	1.65E-08	Up

SS1G_00775	Hypothetical protein	2.92	1.06E-05	Up
SS1G_09135	NADP-dependent alcohol dehydrogenase	2.91	1.53E-07	Up
SS1G_00394	Hypothetical protein	2.90	3.01E-07	Up
SS1G_10223	Hypothetical protein	2.90	4.12E-07	Up
SS1G_00871	Transfer/carrier protein	2.90	1.38E-11	Up
SS1G_06244	Hypothetical protein	2.88	6.65E-11	Up
SS1G_08039	Hypothetical protein	2.87	2.03E-06	Up
SS1G_04247	Hypothetical protein	2.86	2.75E-07	Up
SS1G_10796	Oxalate decarboxylase	2.85	9.48E-06	Up
SS1G_06394	Guanyl-specific ribonuclease	2.83	9.33E-05	Up
SS1G_00733	Alcohol dehydrogenase	2.83	2.98E-09	Up
SS1G_09219	Hypothetical protein	2.82	4.97E-05	Up
SS1G_08628	Efflux pump/transporter	2.79	1.60E-07	Up
SS1G_04085	Expansin-like protein	2.78	6.19E-07	Up
SS1G_00147	Lyase	2.76	1.10E-08	Up
SS1G_13852	ABC transporter B family member	2.75	5.42E-09	Up
SS1G_09322	Hypothetical protein	2.75	1.32E-05	Up
SS1G_10616	Hypothetical protein	2.75	3.12E-04	Up
SS1G_05121	Hypothetical protein	2.74	6.48E-07	Up
SS1G_13982	Para-nitrobenzyl esterase	2.74	1.42E-06	Up
SS1G_04091	Hypothetical protein	2.74	2.07E-04	Up
SS1G_08984	Hypothetical protein	2.74	3.07E-08	Up
SS1G_12235	Putative enoyl-CoA hydratase/isomerase	2.73	1.49E-10	Up
SS1G_06235	Probable rhamnogalacturonase A	2.72	7.03E-07	Up
SS1G_11771	Hypothetical protein	2.71	1.27E-03	Up
SS1G_05042	Hypothetical protein	2.71	8.42E-10	Up
SS1G_03181	Aspartic protease	2.70	1.06E-07	Up
SS1G_05793	Efflux pump vrtL	2.68	2.56E-07	Up
SS1G_13361	Hypothetical protein	2.68	5.00E-06	Up
SS1G_12721	Hypothetical protein	2.68	1.87E-07	Up
SS1G_05794	Hypothetical protein	2.67	1.72E-05	Up
SS1G_01086	Hypothetical protein	2.66	3.83E-05	Up
SS1G_01802	Amino acid transporter	2.66	5.03E-08	Up
SS1G_00758	Hypothetical protein	2.66	4.52E-04	Up
SS1G_01789	Hypothetical protein	2.65	2.90E-03	Up
SS1G_12889	Keratin, type II cytoskeletal 2 epidermal	2.65	4.48E-04	Up
SS1G_05943	Cytochrome P450 monooxygenase	2.65	4.29E-04	Up
SS1G_02770	Aquaporin-like protein	2.63	2.15E-09	Up
SS1G_05006	Glucose transporter	2.62	8.05E-07	Up
SS1G_10495	D-arabinitol dehydrogenase	2.62	8.44E-09	Up
SS1G_03409	Sphingoid long-chain base transporter	2.60	5.00E-06	Up
SS1G_14094	Hypothetical protein	2.59	1.16E-05	Up
SS1G_12812	Pachytene checkpoint protein	2.59	3.50E-09	Up
SS1G_05027	Hypothetical protein	2.59	6.21E-04	Up
SS1G_12231	Hypothetical protein	2.58	1.29E-03	Up
SS1G_02733	Hypothetical protein	2.58	2.79E-05	Up
SS1G_13849	Hypothetical protein	2.57	2.98E-06	Up

SS1G_08143	Hypothetical protein	2.56	3.63E-04	Up
SS1G_08427	Hypothetical protein	2.56	8.04E-07	Up
SS1G_12513	LysM domain-containing protein	2.55	3.24E-06	Up
SS1G_13793	Hypothetical protein	2.54	2.32E-08	Up
SS1G_11699	Probable serine/threonine-protein kinase	2.54	1.99E-05	Up
SS1G_12372	Non-receptor serine/threonine protein kinase	2.53	3.15E-03	Up
SS1G_10944	Nuclear GTPase	2.52	2.54E-06	Up
SS1G_08142	Hypothetical protein	2.52	9.30E-10	Up
SS1G_12506	Hypothetical protein	2.51	2.06E-07	Up
SS1G_10985	Putative pyruvate decarboxylase	2.50	2.60E-05	Up
SS1G_09391	Hypothetical protein	2.50	3.66E-04	Up
SS1G_04376	Hypothetical protein	2.50	3.62E-04	Up
SS1G_11153	Hypothetical protein	2.50	1.40E-03	Up
SS1G_03843	Hypothetical protein	2.50	2.16E-04	Up
SS1G_08560	Hydrolase	2.50	4.99E-04	Up
SS1G_01199	Pyridoxal reductase	2.49	2.86E-04	Up
SS1G_09495	Probable 1-alkyl-2-acetylglycerophosphocholine esterase	2.49	4.97E-05	Up
SS1G_11990	Hypothetical protein	2.49	2.87E-04	Up
SS1G_09984	Hypothetical protein	2.49	6.16E-08	Up
SS1G_06093	Peroxidase	2.48	2.98E-07	Up
SS1G_12776	Hypothetical protein	2.48	7.52E-07	Up
SS1G_06059	Hypothetical protein	2.47	9.37E-05	Up
SS1G_13240	Hypothetical protein	2.46	2.64E-05	Up
SS1G_11281	Hypothetical protein	2.46	3.38E-06	Up
SS1G_05932	NADH-cytochrome b5 reductase	2.46	1.37E-05	Up
SS1G_13234	Efflux pump protein	2.46	6.16E-08	Up
SS1G_14371	Hypothetical protein	2.46	3.89E-05	Up
SS1G_06243	Dehyrogenase	2.44	9.37E-08	Up
SS1G_04361	Hypothetical protein	2.43	1.14E-06	Up
SS1G_12842	Efflux pump protein	2.42	2.44E-05	Up
SS1G_01607	Hypothetical protein	2.41	1.50E-03	Up
SS1G_07983	Hypothetical protein	2.41	1.74E-05	Up
SS1G_07661	Cutinase	2.40	2.14E-06	Up
SS1G_13277	GPI-anchored cupredoxin	2.40	1.54E-04	Up
SS1G_06250	Hypothetical protein	2.39	3.96E-03	Up
SS1G_09448	Hypothetical protein	2.39	1.44E-07	Up
SS1G_03091	Hypothetical protein	2.38	1.89E-07	Up
SS1G_10224	Metaxin-1	2.37	2.53E-07	Up
SS1G_04429	Hypothetical protein	2.37	1.09E-07	Up
SS1G_07894	Hypothetical protein	2.37	1.30E-03	Up
SS1G_01648	Hypothetical protein	2.37	1.20E-04	Up
SS1G_01087	Hypothetical protein	2.36	1.16E-05	Up
SS1G_04548	Hypothetical protein	2.36	2.46E-03	Up
SS1G_07231	Methyltransferase	2.36	9.02E-08	Up
SS1G_02548	Transporter	2.36	6.16E-05	Up

SS1G_10070	Hypothetical protein	2.35	4.75E-05	Up
SS1G_11499	Antibacterial response protein/glucosidase	2.34	1.96E-06	Up
SS1G_10279	Efflux pump protein	2.34	6.31E-05	Up
SS1G_13080	Hypothetical protein	2.34	1.20E-04	Up
SS1G_07227	Hypothetical protein	2.34	5.05E-08	Up
SS1G_08468	Hypothetical protein	2.33	2.33E-03	Up
SS1G_11929	Hypothetical protein	2.33	1.51E-03	Up
SS1G_07228	Polyamine transporter	2.33	2.69E-06	Up
SS1G_08387	Amino acid transporter	2.33	1.36E-06	Up
SS1G_03167	Hypothetical protein	2.33	2.49E-04	Up
SS1G_11539	Terpene cyclase	2.32	2.05E-07	Up
SS1G_02882	Alternative oxidase, mitochondrial	2.32	3.10E-06	Up
SS1G_01793	Hypothetical protein	2.31	3.31E-07	Up
SS1G_07294	Decarboxylase	2.31	2.86E-03	Up
SS1G_02356	Reducing polyketide synthase	2.30	4.57E-04	Up
SS1G_07627	Hypothetical protein	2.30	6.19E-05	Up
SS1G_05890	Hypothetical protein	2.29	5.77E-07	Up
SS1G_05789	Probable esterase	2.29	3.88E-04	Up
SS1G_04303	Hypothetical protein	2.29	4.68E-04	Up
SS1G_13735	Hypothetical protein	2.29	7.67E-04	Up
SS1G_05394	Cytochrome P450/oxygenase	2.29	1.62E-03	Up
SS1G_02434	Putative mitochondrial 2-oxoglutarate/malate carrier protein	2.28	1.49E-04	Up
SS1G_05548	Hypothetical protein	2.28	6.30E-06	Up
SS1G_01770	Hypothetical protein	2.28	3.72E-04	Up
SS1G_05834	Forkhead box DNA binding protein	2.27	1.43E-03	Up
SS1G_06060	Oxidoreductase	2.26	1.38E-04	Up
SS1G_01952	Acyl-CoA dehydrogenase	2.25	1.32E-06	Up
SS1G_04903	Calcium-binding protein	2.25	2.94E-05	Up
SS1G_13648	Hypothetical protein	2.25	2.78E-05	Up
SS1G_06168	Hypothetical protein	2.25	1.41E-03	Up
SS1G_01993	Hypothetical protein	2.24	2.51E-04	Up
SS1G_12229	Hypothetical protein	2.24	5.59E-04	Up
SS1G_12897	30 kDa heat shock protein	2.24	1.02E-06	Up
SS1G_05329	Aspartic protease	2.24	1.60E-03	Up
SS1G_11478	Ankyrin-2	2.23	3.50E-06	Up
SS1G_05889	Hypothetical protein	2.23	1.21E-07	Up
SS1G_03506	Hypothetical protein	2.22	6.69E-04	Up
SS1G_13001	Transcriptional regulatory protein moc3	2.22	2.51E-08	Up
SS1G_11846	Hypothetical protein	2.21	1.54E-05	Up
SS1G_04306	Zinc-type alcohol dehydrogenase	2.21	1.66E-04	Up
SS1G_12276	Hypothetical protein	2.21	2.23E-07	Up
SS1G_01387	Hypothetical protein	2.20	7.89E-05	Up
SS1G_05576	Glycosyltransferase	2.19	1.09E-03	Up
SS1G_08893	Hypothetical protein	2.18	9.91E-08	Up
SS1G_04351	Hypothetical protein	2.16	2.86E-04	Up
SS1G_11522	Hypothetical protein	2.16	5.07E-07	Up

SS1G_08229	Probable rhamnogalacturonase A	2.16	5.00E-04	Up
SS1G_10309	Hypothetical protein	2.15	2.97E-04	Up
SS1G_08351	Xylanolytic transcriptional activator xlnR homolog	2.15	5.89E-05	Up
SS1G_07293	Hypothetical protein	2.14	5.55E-04	Up
SS1G_05832	Probable exopolygalacturonase X	2.14	9.06E-05	Up
SS1G_10193	NmrA-like family domain-containing protein	2.13	2.49E-04	Up
SS1G_01084	Hypothetical protein	2.11	4.31E-03	Up
SS1G_11498	Choline-sulfatase	2.10	1.41E-05	Up
SS1G_11770	Hypothetical protein	2.09	4.41E-04	Up
SS1G_09724	Acyltransferase	2.09	1.26E-04	Up
SS1G_04371	Hypothetical protein	2.09	6.54E-05	Up
SS1G_03783	Hypothetical protein	2.08	7.54E-04	Up
SS1G_12775	Serine protease	2.08	2.38E-06	Up
SS1G_09730	Nitroalkane oxidase	2.08	1.73E-06	Up
SS1G_04145	Hypothetical protein	2.07	3.93E-04	Up
SS1G_02500	Splicing regulatory glutamine/lysine-rich protein 1	2.07	6.42E-06	Up
SS1G_03116	Hypothetical protein	2.07	3.99E-04	Up
SS1G_11958	Hypothetical protein	2.06	1.42E-04	Up
SS1G_02412	Hypothetical protein	2.06	3.29E-05	Up
SS1G_10898	Hypothetical protein	2.06	2.68E-05	Up
SS1G_13809	Glucoamylase	2.05	1.14E-05	Up
SS1G_00501	Probable xyloglucan-specific endo-beta-1,4-glucanase	2.05	2.57E-03	Up
SS1G_14240	Hypothetical protein	2.04	4.75E-05	Up
SS1G_07847	Beta-glucosidase	2.04	9.68E-05	Up
SS1G_11853	Carboxylesterase patB	2.04	1.14E-05	Up
SS1G_05791	Hypothetical protein	2.03	9.09E-04	Up
SS1G_09537	Serine protease	2.02	1.30E-04	Up
SS1G_08348	Zinc-type alcohol dehydrogenase	2.02	3.99E-04	Up
SS1G_02793	Transcriptional activator protein	2.01	1.08E-03	Up
SS1G_14006	Endo-1,4-beta-xylanase	2.01	2.92E-03	Up
SS1G_09446	Acyltransferase	2.01	1.39E-03	Up
SS1G_01229	Probable glucan endo-1,3-beta-glucosidase ARB 02077	2.01	1.14E-05	Up
SS1G_07863	Cellobiose dehydrogenase	1.98	3.92E-05	Up
SS1G_07152	Hypothetical protein	1.98	1.14E-04	Up
SS1G_10428	Hypothetical protein	1.98	5.79E-04	Up
SS1G_08837	Endoglucanase	1.98	3.09E-03	Up
SS1G_05368	Beta-glucosidase	1.98	1.45E-05	Up
SS1G_04080	Methyltransferase	1.98	4.31E-04	Up
SS1G_04343	Hypothetical protein	1.97	2.32E-04	Up
SS1G_03220	Carbonyl reductase family member	1.97	4.81E-03	Up
SS1G_03166	Hypothetical protein	1.96	2.49E-04	Up
SS1G_05758	Hypothetical protein	1.96	9.23E-06	Up
SS1G_10092	Endo-1,4-beta-xylanase	1.95	2.70E-05	Up

SS1G_14291	Microperfuranone synthase	1.94	3.83E-05	Up
SS1G_05048	Hypothetical protein	1.94	4.69E-03	Up
SS1G_02384	Hypothetical protein	1.94	1.56E-06	Up
SS1G_00751	Hypothetical protein	1.93	1.29E-04	Up
SS1G_05798	Hypothetical protein	1.93	1.53E-03	Up
SS1G_01953	Acetylesterase	1.93	4.57E-04	Up
SS1G_02703	Hypothetical protein	1.92	1.37E-03	Up
SS1G_10566	MFS-type transporter	1.91	1.41E-05	Up
SS1G_02880	Hypothetical protein	1.91	1.09E-03	Up
SS1G_05679	6-hydroxy-D-nicotine oxidase	1.91	2.09E-05	Up
SS1G_04273	High-affinity gluconate transporte	1.90	2.06E-05	Up
SS1G_09928	Hypothetical protein	1.90	5.06E-04	Up
SS1G_03880	Hypothetical protein	1.90	4.75E-04	Up
SS1G_13386	Cutinase	1.90	7.66E-04	Up
SS1G_02161	Oxygenase	1.90	1.57E-05	Up
SS1G_04095	Rhamnogalacturonan acetylesterase	1.89	2.38E-04	Up
SS1G_09732	Purine permease	1.89	6.57E-06	Up
SS1G_04694	Altered inheritance of mitochondria protein	1.89	1.79E-04	Up
SS1G_08006	Telomerase-binding protein	1.88	3.85E-04	Up
SS1G_05681	Norsolorinic acid synthase	1.88	6.90E-06	Up
SS1G_08638	Pentatricopeptide repeat-containing protein, mitochondrial	1.88	2.92E-03	Up
SS1G_00241	Hypothetical protein	1.87	1.23E-05	Up
SS1G_08026	Hypothetical protein	1.87	1.02E-03	Up
SS1G_05906	Regulator of biquitin conjugation	1.86	1.29E-03	Up
SS1G_02717	ATP-dependent DNA helicase subunit	1.86	9.04E-06	Up
SS1G_05792	Carboxypeptidase Y inhibitor	1.86	7.51E-05	Up
SS1G_11395	Hypothetical protein	1.85	2.38E-04	Up
SS1G_04757	Iron import ATP-binding/permease protein	1.85	1.13E-04	Up
SS1G_12511	Hypothetical protein	1.85	1.14E-04	Up
SS1G_00767	Hypothetical protein	1.84	4.31E-03	Up
SS1G_00151	Cytochrome P450 monooxygenase	1.84	2.25E-03	Up
SS1G_06035	Dehydrogenase	1.84	1.02E-04	Up
SS1G_01523	Lactose permease	1.84	4.75E-05	Up
SS1G_08558	Serine protease	1.83	1.18E-03	Up
SS1G_06281	Ankyrin repeat domain-containing protein	1.83	2.92E-03	Up
SS1G_08204	Hypothetical protein	1.82	1.01E-03	Up
SS1G_01177	Hypothetical protein	1.82	3.62E-03	Up
SS1G_05487	Hydrolase	1.82	1.71E-03	Up
SS1G_13917	Hypothetical protein	1.82	7.04E-04	Up
SS1G_04994	Hypothetical protein	1.82	4.18E-03	Up
SS1G_00557	Hypothetical protein	1.82	8.54E-04	Up
SS1G_02831	Hypothetical protein	1.81	2.59E-03	Up
SS1G_06601	Ammonium transporter	1.81	1.98E-04	Up
SS1G_08045	Hypothetical protein	1.81	2.32E-04	Up
SS1G_02838	Hypothetical protein	1.79	4.67E-03	Up
SS1G_07854	Acetyltransferase	1.78	7.26E-04	Up

SS1G_14049	Endoribonuclease	1.78	3.06E-03	Up
SS1G_06339	Hypothetical protein	1.78	5.72E-05	Up
SS1G_11394	Isoflavone reductase homolog	1.78	3.50E-03	Up
SS1G_01406	Serine protease	1.78	4.10E-05	Up
SS1G_14440	Disulfide-bond oxidoreductase	1.77	2.95E-04	Up
SS1G_07720	Chaperone	1.77	4.62E-05	Up
SS1G_07758	Hypothetical protein	1.77	2.50E-05	Up
SS1G_01871	Putative thiosulfate sulfurtransferase, mitochondrial	1.77	6.61E-04	Up
SS1G_11915	Hypothetical protein	1.77	9.56E-04	Up
SS1G_06308	Hypothetical protein	1.77	5.38E-04	Up
SS1G_11068	tRNA amidotransferase subunit	1.77	5.03E-04	Up
SS1G_04249	Isomerase, peroxisomal	1.76	1.90E-04	Up
SS1G_12062	Methylsalicylic acid decarboxylase	1.75	1.56E-03	Up
SS1G_02429	Sugar transporter	1.75	1.44E-04	Up
SS1G_08624	Hypothetical protein	1.74	1.69E-04	Up
SS1G_02363	Cytochrome P450 oxygenase 3A8	1.74	1.64E-04	Up
SS1G_04515	Phosphatase	1.74	2.89E-04	Up
SS1G_11982	Hypothetical protein	1.73	2.55E-03	Up
SS1G_12660	Hypothetical protein	1.73	2.22E-03	Up
SS1G_07499	Diadenosine 5',5'''-P1,P4-tetraphosphate phosphorylase	1.72	5.62E-04	Up
SS1G_10115	non-LTR retrotransposon	1.72	1.69E-04	Up
SS1G_09381	Endochitinase	1.72	1.74E-03	Up
SS1G_10943	Regulatory protein cys-3	1.72	2.08E-04	Up
SS1G_04912	Hypothetical protein	1.72	1.66E-03	Up
SS1G_05680	Monooxygenase	1.72	1.24E-04	Up
SS1G_14427	Hypothetical protein	1.71	7.20E-04	Up
SS1G_14289	Acetylesterase	1.70	1.08E-03	Up
SS1G_13919	Hypothetical protein	1.70	1.57E-03	Up
SS1G_05407	Glucokinase	1.70	2.86E-04	Up
SS1G_14133	Hypothetical protein	1.70	3.53E-05	Up
SS1G_02586	Phenylalanine aminomutase	1.69	4.56E-03	Up
SS1G_13155	Endochitinase 2	1.69	1.92E-03	Up
SS1G_12507	Actin family cytoskeletal protein	1.69	1.07E-03	Up
SS1G_01217	Non-canonical non-ribosomal peptide synthetase	1.69	1.94E-03	Up
SS1G_08642	Oxygenase	1.69	2.99E-04	Up
SS1G_07667	Clock-controlled protein 6	1.68	9.08E-04	Up
SS1G_13451	Hypothetical protein	1.68	9.93E-05	Up
SS1G_08383	Probable 6-phosphogluconolactonase	1.68	1.59E-03	Up
SS1G_04248	Putative lysine N-acyltransferase	1.68	3.63E-04	Up
SS1G_05900	Hypothetical protein	1.68	3.27E-04	Up
SS1G_10088	Trichodiene oxygenase	1.67	2.20E-03	Up
SS1G_10155	Transporter	1.67	2.08E-03	Up
SS1G_02395	HECT-type ubiquitin ligase-interacting protein	1.67	4.48E-04	Up

SS1G_03737	TY3 family retrotransposon	1.67	5.03E-04	Up
SS1G_08144	Dehydrogenase	1.66	2.19E-03	Up
SS1G_11525	Hypothetical protein	1.66	4.73E-04	Up
SS1G_14439	Phospholipase	1.66	4.63E-04	Up
SS1G_04272	Hypothetical protein	1.65	3.56E-03	Up
SS1G_02425	Hypothetical protein	1.65	1.60E-03	Up
SS1G_08531	Hypothetical protein	1.64	2.87E-03	Up
SS1G_10427	TY3 family retrotransposon	1.64	2.20E-04	Up
SS1G_09978	Peptidase	1.64	1.92E-04	Up
SS1G_12427	Methyltransferase	1.64	3.69E-04	Up
SS1G_01156	TY3 family retrotransposon	1.64	8.87E-04	Up
SS1G_03989	O-methyltransferase	1.64	1.52E-03	Up
SS1G_13522	Diacetyl reductase	1.63	2.07E-03	Up
SS1G_02456	Hypothetical protein	1.62	1.39E-03	Up
SS1G_06869	Retrotransposon	1.62	1.64E-04	Up
SS1G_08910	Hypothetical protein	1.61	3.68E-03	Up
SS1G_02385	Hypothetical protein	1.61	1.30E-03	Up
SS1G_06247	Hypothetical protein	1.61	2.33E-04	Up
SS1G_12770	Ketodihydrosphingosine reductase	1.61	2.53E-04	Up
SS1G_03174	Hypothetical protein	1.60	8.52E-04	Up
SS1G_14238	Dimethyl-sulfide monooxygenase	1.60	4.72E-04	Up
SS1G_06873	Homogentisate 1,2-dioxygenase	1.58	3.73E-03	Up
SS1G_05678	Methyltransferase	1.58	2.07E-04	Up
SS1G_05434	Hypothetical protein	1.58	1.74E-03	Up
SS1G_12679	Gluconate 5-dehydrogenase	1.58	5.48E-04	Up
SS1G_01718	Transfer/carrier protein	1.57	4.05E-03	Up
SS1G_11568	Hypothetical protein	1.57	1.77E-03	Up
SS1G_13764	Hypothetical protein	1.57	1.56E-03	Up
SS1G_00795	Glutamate decarboxylase	1.57	6.61E-04	Up
SS1G_08257	Glutamine amidotransferase	1.56	7.19E-04	Up
SS1G_04336	Efflux pump protein	1.56	4.14E-03	Up
SS1G_14404	Choline transport protein	1.56	3.68E-03	Up
SS1G_10067	D-galactonate dehydratase	1.56	4.49E-03	Up
SS1G_02399	Rhamnogalacturonase	1.55	1.21E-03	Up
SS1G_05839	Hypothetical protein	1.55	2.98E-03	Up
SS1G_03962	Argininosuccinate lyase	1.54	2.78E-03	Up
SS1G_09965	Sphingomyelin phosphodiesterase	1.54	6.31E-04	Up
SS1G_10614	U4/U6.U5 tri-snRNP-associated protein 1	1.54	5.88E-04	Up
SS1G_05674	FAD-linked oxidoreductase	1.54	2.05E-03	Up
SS1G_04888	Methyltransferase-like protein	1.53	8.87E-04	Up
SS1G_08425	Sugar transporter	1.52	3.73E-03	Up
SS1G_05356	Hypothetical protein	1.52	1.00E-03	Up
SS1G_06254	Hypothetical protein	1.52	3.56E-03	Up
SS1G_00736	Dehydrogenase	1.52	5.81E-04	Up
SS1G_13112	ATP-binding cassette (ABC) transporter	1.51	2.51E-03	Up
SS1G_06665	Methyltransferase	1.51	2.98E-03	Up
SS1G_07050	Ankyrin-like protein	1.50	1.51E-03	Up

SS1G_11055	Membrane protein	1.50	3.04E-03	Up
SS1G_10251	Glutamate-cysteine ligase regulatory subunit	1.49	4.77E-03	Up
SS1G_12287	Hypothetical protein	1.49	2.68E-03	Up
SS1G_08745	Hypothetical protein	1.48	1.71E-03	Up
SS1G_00014	Hypothetical protein	1.47	7.82E-04	Up
SS1G_05675	Hypothetical protein	1.47	1.93E-03	Up
SS1G_01915	Chromatin/chromatin-binding protein	1.47	1.96E-03	Up
SS1G_05313	Efflux pump protien	1.46	1.86E-03	Up
SS1G_07511	Hypothetical protein	1.46	4.67E-03	Up
SS1G_11223	Hypothetical protein	1.45	3.18E-03	Up
SS1G_11603	ATP-dependent 6-phosphofructokinase 1	1.44	2.61E-03	Up
SS1G_02776	Catechol 1,2-dioxygenase	1.43	4.43E-03	Up
SS1G_03293	Hypothetical protein	1.40	2.46E-03	Up
SS1G_02049	Cytochrome P450 monooxygenase	1.39	4.11E-03	Up
SS1G_03286	Pectinesterase	1.39	4.69E-03	Up
SS1G_02870	aspartic protease	1.39	2.94E-03	Up
SS1G_04945	Exoglucanase 1	1.37	2.78E-03	Up
SS1G_04099	Hydroxypropyl-CoM dehydrogenase	1.34	4.47E-03	Up
SS1G_00744	Hypothetical protein	1.32	3.50E-03	Up
SS1G_05676	Dehydrogenase	1.29	4.43E-03	Up
SS1G_05980	Cytochrome P450 52A5	1.28	4.94E-03	Up
SS1G_13505	Hypothetical protein	10.10	2.64E-118	Down
SS1G_10484	Hypothetical protein	9.59	5.10E-78	Down
SS1G_08790	Hypothetical protein	9.19	3.17E-97	Down
SS1G_02828	Hypothetical protein	9.03	2.86E-72	Down
SS1G_08163	Hypothetical protein	8.97	4.29E-89	Down
SS1G_13844	Hypothetical protein	8.51	5.82E-62	Down
SS1G_05939	Hypothetical protein	8.49	8.79E-74	Down
SS1G_09248	Cryparin	8.34	1.88E-88	Down
SS1G_10485	Zinc finger transcription factor	8.10	2.38E-66	Down
SS1G_13274	Hypothetical protein	7.05	1.09E-47	Down
SS1G_02695	Hypothetical protein	6.28	3.60E-28	Down
SS1G_13965	Hypothetical protein	6.27	7.46E-25	Down
SS1G_03616	Dehydrogenase	6.22	2.74E-45	Down
SS1G_11468	Hypothetical protein	6.20	1.14E-41	Down
SS1G_09270	Hypothetical protein	5.45	1.37E-28	Down
SS1G_10466	Pdp3-interacting factor 1	4.95	1.97E-28	Down
SS1G_05118	Beta-1,3-glucan-binding protein	4.78	9.00E-20	Down
SS1G_02692	Hypothetical protein	4.76	8.99E-24	Down
SS1G_00854	Oxidoreductase	4.71	2.37E-16	Down
SS1G_03430	Hypothetical protein	4.64	1.21E-25	Down
SS1G_12357	Hypothetical protein	4.61	1.02E-27	Down
SS1G_06527	Hypothetical protein	4.56	1.23E-14	Down
SS1G_05112	Laccase	4.55	2.74E-22	Down
SS1G_02163	Versiconal hemiacetal acetate esterase	4.51	7.05E-25	Down
SS1G_05223	Methyltransferase	4.49	8.93E-19	Down
SS1G_07262	Hypothetical protein	4.48	2.19E-17	Down

SS1G_01478	Sphingolipid C9-methyltransferase	4.47	1.35E-31	Down
SS1G_13696	Hypothetical protein	4.45	3.87E-18	Down
SS1G_05517	Hypothetical protein	4.40	2.63E-11	Down
SS1G_11618	Hypothetical protein	4.38	2.16E-17	Down
SS1G_01572	Beta-galactosidase	4.33	4.15E-25	Down
SS1G_04171	Hypothetical protein	4.29	4.33E-20	Down
SS1G_02727	Repressible high-affinity phosphate permease	4.28	4.22E-22	Down
SS1G_05775	Glucan 1,3-beta-glucosidase	4.27	3.61E-24	Down
SS1G_05972	Hypothetical protein	4.19	3.22E-13	Down
SS1G_09905	Hypothetical protein	4.14	5.20E-11	Down
SS1G_10311	Hypothetical protein	4.11	1.61E-22	Down
SS1G_13633	Serine protease	4.11	1.13E-09	Down
SS1G_11104	Hypothetical protein	4.10	1.19E-24	Down
SS1G_05518	Hypothetical protein	4.09	6.13E-11	Down
SS1G_01426	Hypothetical protein	4.09	1.91E-12	Down
SS1G_10892	Hypothetical protein	4.09	1.02E-11	Down
SS1G_12361	Hypothetical protein	4.08	1.14E-15	Down
SS1G_02879	Hypothetical protein	4.08	3.71E-19	Down
SS1G_02694	Glycosyltransferase	4.07	7.91E-19	Down
SS1G_08110	Hypothetical protein	4.04	1.15E-14	Down
SS1G_01859	Hypothetical protein	4.04	5.09E-20	Down
SS1G_06526	Hypothetical protein	4.04	3.08E-12	Down
SS1G_05870	Hypothetical protein	4.00	3.43E-09	Down
SS1G_08397	Hypothetical protein	3.99	3.89E-23	Down
SS1G_07752	Hypothetical protein	3.96	6.24E-13	Down
SS1G_04563	Decarboxylase	3.95	9.76E-15	Down
SS1G_13009	Hypothetical protein	3.95	1.17E-07	Down
SS1G_02250	Hypothetical protein	3.93	3.09E-11	Down
SS1G_14398	Hypothetical protein	3.90	2.06E-24	Down
SS1G_08398	Sugar transporter	3.87	6.11E-20	Down
SS1G_07042	Hypothetical protein	3.85	2.97E-07	Down
SS1G_10608	Hypothetical protein	3.84	9.10E-17	Down
SS1G_09106	Hypothetical protein	3.84	1.03E-07	Down
SS1G_08680	Hypothetical protein	3.83	5.53E-09	Down
SS1G_01364	Hypothetical protein	3.81	5.26E-08	Down
SS1G_10483	Hypothetical protein	3.80	7.67E-05	Down
SS1G_13758	Hypothetical protein	3.79	4.35E-08	Down
SS1G_01265	Nonribosomal peptide synthetase	3.78	1.38E-15	Down
SS1G_13843	Hypothetical protein	3.76	1.33E-05	Down
SS1G_13636	Cyclochlorotine biosynthesis protein	3.73	2.63E-09	Down
SS1G_12200	Dehydrogenase	3.72	2.78E-13	Down
SS1G_11120	Hypothetical protein	3.70	1.17E-07	Down
SS1G_09562	Hypothetical protein	3.69	1.55E-04	Down
SS1G_09110	Glucan endo-1,3-beta-glucosidase	3.62	4.25E-13	Down
SS1G_10936	Extracellular membrane protein	3.62	1.68E-16	Down
SS1G_12806	Hypothetical protein	3.62	1.00E-06	Down

SS1G_14108	Calcium ion transporter	3.61	3.10E-19	Down
SS1G_13635	Hypothetical protein	3.60	9.32E-08	Down
SS1G_08080	Hypothetical protein	3.60	3.08E-15	Down
SS1G_01759	Sugar transporter	3.56	3.61E-09	Down
SS1G_00263	Hypothetical protein	3.55	1.05E-06	Down
SS1G_07359	Hypothetical protein	3.55	1.44E-13	Down
SS1G_02693	Hypothetical protein	3.53	1.09E-15	Down
SS1G_04590	Hypothetical protein	3.52	6.18E-11	Down
SS1G_12181	Hypothetical protein	3.52	2.88E-18	Down
SS1G_13619	Hypothetical protein	3.51	6.37E-12	Down
SS1G_03084	Hypothetical protein	3.46	2.71E-09	Down
SS1G_00506	Hypothetical protein	3.46	5.85E-07	Down
SS1G_14307	Extracellular membrane protein	3.45	3.71E-19	Down
SS1G_06053	Hypothetical protein	3.44	7.28E-13	Down
SS1G_13637	Hypothetical protein	3.41	5.34E-07	Down
SS1G_13024	Efflux pump protein	3.41	6.25E-17	Down
SS1G_14000	Hypothetical protein	3.40	1.67E-06	Down
SS1G_11673	Hypothetical protein	3.39	3.42E-12	Down
SS1G_03615	Hypothetical protein	3.38	3.45E-14	Down
SS1G_01657	Linoleate diol synthase	3.38	3.71E-19	Down
SS1G_07263	Hypothetical protein	3.37	8.70E-07	Down
SS1G_12394	Hypothetical protein	3.36	5.09E-08	Down
SS1G_03266	Hypothetical protein	3.36	5.43E-04	Down
SS1G_08681	Homeobox protein	3.34	5.62E-15	Down
SS1G_08644	Lipase	3.34	9.84E-15	Down
SS1G_13613	Hypothetical protein	3.31	1.10E-09	Down
SS1G_11189	Metallocarboxypeptidase	3.31	1.08E-05	Down
SS1G_10505	Hypothetical protein	3.29	1.10E-15	Down
SS1G_13348	Hypothetical protein	3.26	2.41E-16	Down
SS1G_04362	Hypothetical protein	3.24	1.30E-12	Down
SS1G_13618	Hypothetical protein	3.24	3.74E-07	Down
SS1G_14015	Hypothetical protein	3.24	2.54E-14	Down
SS1G_14401	Hypothetical protein	3.24	1.31E-13	Down
SS1G_10099	Hypothetical protein	3.23	4.27E-13	Down
SS1G_01003	Hypothetical protein	3.21	9.57E-05	Down
SS1G_06826	Hypothetical protein	3.21	7.59E-08	Down
SS1G_00105	Hypothetical protein	3.18	3.45E-14	Down
SS1G_12456	Hypothetical protein	3.16	4.02E-15	Down
SS1G_11992	Rhamnogalacturonan acetylesterase	3.16	1.19E-06	Down
SS1G_09246	Hypothetical protein	3.16	1.74E-06	Down
SS1G_04213	GPI-anchored CFEM domain protein A	3.16	2.98E-09	Down
SS1G_04136	Hypothetical protein	3.16	1.88E-06	Down
SS1G_12633	Hypothetical protein	3.15	2.72E-12	Down
SS1G_04137	Hypothetical protein	3.15	8.97E-14	Down
SS1G_03614	Hypothetical protein	3.14	8.26E-14	Down
SS1G_02616	Hypothetical protein	3.12	5.65E-13	Down
SS1G_11994	Hypothetical protein	3.11	2.57E-15	Down

SS1G_06825	Hypothetical protein	3.11	4.75E-05	Down
SS1G_02615	Hypothetical protein	3.10	8.35E-07	Down
SS1G_13906	Efflux pump protein	3.10	4.33E-11	Down
SS1G_04898	Probable peptidoglycan-N-acetylglucosamine deacetylase	3.10	3.65E-14	Down
SS1G_06542	Non-receptor serine/threonine-protein kinase SKS1	3.09	1.98E-13	Down
SS1G_07900	Signal transduction histidine kinase	3.09	1.03E-14	Down
SS1G_02022	Secreted glycosidase	3.09	5.06E-12	Down
SS1G_10082	Hypothetical protein	3.09	5.03E-10	Down
SS1G_02251	Hypothetical protein	3.08	4.55E-09	Down
SS1G_00084	Hypothetical protein	3.07	1.59E-03	Down
SS1G_12808	Hypothetical protein	3.07	2.14E-13	Down
SS1G_02729	Hypothetical protein	3.06	6.98E-05	Down
SS1G_11210	Hypothetical protein	3.01	3.37E-07	Down
SS1G_02718	Hypothetical protein	3.00	6.60E-04	Down
SS1G_06769	Hypothetical protein	2.99	1.20E-12	Down
SS1G_02826	Haloacetate dehalogenase	2.99	1.22E-14	Down
SS1G_00095	Hypothetical protein	2.98	4.19E-07	Down
SS1G_01754	Hypothetical protein	2.95	6.96E-09	Down
SS1G_02001	Hypothetical protein	2.94	4.97E-05	Down
SS1G_13922	Tripeptidyl-peptidase	2.94	4.77E-08	Down
SS1G_10098	Hypothetical protein	2.94	8.26E-06	Down
SS1G_11073	Hypothetical protein	2.94	8.30E-05	Down
SS1G_09768	Hypothetical protein	2.93	1.11E-07	Down
SS1G_11364	Hypothetical protein	2.92	6.41E-05	Down
SS1G_01976	2-methylcitrate dehydratase	2.90	3.20E-08	Down
SS1G_10948	Hypothetical protein	2.90	9.95E-08	Down
SS1G_12708	Cytochrome P450 monooxygenase	2.89	4.33E-14	Down
SS1G_00216	Hypothetical protein	2.89	1.75E-04	Down
SS1G_05079	Transferase	2.88	5.03E-08	Down
SS1G_11100	Trehalose-6-phosphate hydrolase	2.87	1.30E-10	Down
SS1G_13013	Hypothetical protein	2.86	2.96E-13	Down
SS1G_03388	Hypothetical protein	2.86	7.17E-05	Down
SS1G_06670	Hypothetical protein	2.84	3.72E-09	Down
SS1G_05999	Hypothetical protein	2.84	4.31E-11	Down
SS1G_05204	Hypothetical protein	2.83	9.52E-05	Down
SS1G_06862	Leukocyte receptor cluster member 8 homolog	2.83	1.07E-06	Down
SS1G_03683	Hypothetical protein	2.81	6.44E-09	Down
SS1G_10456	Hypothetical protein	2.81	2.24E-05	Down
SS1G_10096	Protein SnodProt1	2.81	2.07E-10	Down
SS1G_04208	Sugar transporter	2.79	2.39E-09	Down
SS1G_00104	Hypothetical protein	2.78	8.35E-08	Down
SS1G_12385	1-phosphatidylinositol phosphodiesterase	2.77	6.33E-08	Down
SS1G_00036	Glucose-repressible gene protein	2.76	2.51E-09	Down
SS1G_08930	Hypothetical protein	2.74	8.20E-07	Down

SS1G_06493	Transferase	2.74	2.15E-09	Down
SS1G_02613	Hypothetical protein	2.73	1.66E-03	Down
SS1G_09265	Hypothetical protein	2.72	2.75E-07	Down
SS1G_07816	Hypothetical protein	2.72	2.98E-06	Down
SS1G_11356	Hypothetical protein	2.70	5.75E-05	Down
SS1G_00484	Cell-wall-anchored protein	2.70	1.63E-04	Down
SS1G_13697	Hypothetical protein	2.70	7.67E-04	Down
SS1G_14320	Hypothetical protein	2.70	9.34E-11	Down
SS1G_14032	Alkali-sensitive linkage protein	2.69	1.07E-09	Down
SS1G_09101	Hypothetical protein	2.69	5.14E-10	Down
SS1G_00849	Hypothetical protein	2.69	1.21E-04	Down
SS1G_07261	Hypothetical protein	2.68	1.67E-04	Down
SS1G_06948	Metalloprotease(	2.68	3.51E-10	Down
SS1G_01655	Hypothetical protein	2.68	4.27E-06	Down
SS1G_06535	Amino acid transporter	2.67	2.91E-09	Down
SS1G_06534	Aerine protease	2.66	3.49E-10	Down
SS1G_01654	Hypothetical protein	2.66	2.59E-05	Down
SS1G_01045	Pheromone-regulated membrane protein	2.66	2.96E-10	Down
SS1G_11674	Hypothetical protein	2.63	1.20E-06	Down
SS1G_09782	Nuclease P1	2.62	3.49E-06	Down
SS1G_05299	Hypothetical protein	2.62	1.17E-03	Down
SS1G_12608	Hypothetical protein	2.61	1.12E-07	Down
SS1G_12488	Myb-like DNA-binding protein	2.61	1.11E-09	Down
SS1G_01530	G-protein coupled receptor	2.60	1.36E-08	Down
SS1G_00601	Hypothetical protein	2.60	1.11E-05	Down
SS1G_10609	Hypothetical protein	2.59	4.97E-05	Down
SS1G_01428	Hypothetical protein	2.58	1.94E-03	Down
SS1G_01414	Hypothetical protein	2.57	4.88E-03	Down
SS1G_04564	Hypothetical protein	2.57	3.78E-04	Down
SS1G_06541	Hypothetical protein	2.57	5.24E-05	Down
SS1G_03913	Hypothetical protein	2.56	1.26E-09	Down
SS1G_05306	Hypothetical protein	2.55	6.16E-08	Down
SS1G_02633	Hypothetical protein	2.55	1.26E-08	Down
SS1G_08682	Hypothetical protein	2.55	2.76E-03	Down
SS1G_09838	Agglutinin	2.55	4.35E-04	Down
SS1G_09264	Hypothetical protein	2.54	3.71E-06	Down
SS1G_03784	Amino acid transporter	2.54	8.71E-07	Down
SS1G_07699	Hypothetical protein	2.53	2.12E-08	Down
SS1G_05103	Hypothetical protein	2.52	2.83E-09	Down
SS1G_01658	Hypothetical protein	2.52	3.50E-05	Down
SS1G_14396	Alcohol dehydrogenase	2.52	5.05E-06	Down
SS1G_06770	Hypothetical protein	2.52	7.43E-04	Down
SS1G_00357	Probable GPI-anchored cupredoxin	2.51	3.12E-07	Down
SS1G_06940	Ribonucleoprotein	2.51	2.48E-05	Down
SS1G_10610	Hypothetical protein	2.51	5.80E-06	Down
SS1G_06133	Dehydrogenase	2.50	4.57E-07	Down
SS1G_03299	Hypothetical protein	2.50	2.11E-09	Down

SS1G_04107	Hypothetical protein	2.49	1.46E-08	Down
SS1G_04540	NADH-cytochrome reductase	2.49	9.73E-07	Down
SS1G_01411	Hypothetical protein	2.49	8.51E-05	Down
SS1G_11579	Alpha-mannosidase	2.49	2.63E-05	Down
SS1G_13923	Cytochrome P450 monooxygenase	2.49	5.09E-08	Down
SS1G_13051	Cellobiose dehydrogenase	2.48	6.48E-07	Down
SS1G_09646	Hypothetical protein	2.46	8.31E-07	Down
SS1G_13683	Fatty acid desaturase	2.45	3.91E-08	Down
SS1G_11009	Hypothetical protein	2.45	1.13E-06	Down
SS1G_14495	Hypothetical protein	2.45	4.25E-06	Down
SS1G_01613	Hypothetical protein	2.45	3.11E-05	Down
SS1G_05457	Hypothetical protein	2.44	5.48E-09	Down
SS1G_12109	Hypothetical protein	2.44	3.25E-04	Down
SS1G_06307	Hypothetical protein	2.42	1.60E-07	Down
SS1G_01293	Hypothetical protein	2.42	1.43E-07	Down
SS1G_12337	Vacuolar calcium ion transporter	2.42	7.58E-10	Down
SS1G_10746	Hypothetical protein	2.40	7.08E-09	Down
SS1G_10947	Hypothetical protein	2.40	4.72E-08	Down
SS1G_14316	Proton myo-inositol cotransporter	2.40	9.77E-10	Down
SS1G_01656	Transporter	2.40	4.35E-08	Down
SS1G_02974	Zinc finger protein	2.39	3.84E-06	Down
SS1G_01974	Hypothetical protein	2.39	7.29E-06	Down
SS1G_01292	Hypothetical protein	2.38	1.45E-05	Down
SS1G_06402	Sugar transporter	2.38	8.41E-07	Down
SS1G_05102	Hypothetical protein	2.37	6.64E-07	Down
SS1G_09250	Xyloglucan-specific endo-beta-1,4-glucanase	2.37	2.09E-06	Down
SS1G_05141	Hypothetical protein	2.36	1.02E-06	Down
SS1G_11106	Ribosomal RNA small subunit methyltransferase	2.35	1.98E-08	Down
SS1G_01536	Hypothetical protein	2.35	1.69E-04	Down
SS1G_07968	Hypothetical protein	2.35	4.41E-05	Down
SS1G_06084	Hypothetical protein	2.34	7.30E-04	Down
SS1G_06666	Hypothetical protein	2.34	3.08E-05	Down
SS1G_09216	Beta-glucosidase	2.33	4.59E-08	Down
SS1G_01585	Hypothetical protein	2.32	4.31E-06	Down
SS1G_07965	Aminoacyl-tRNA synthetasec	2.32	4.44E-09	Down
SS1G_03733	Hypothetical protein	2.32	2.97E-06	Down
SS1G_01958	Hypothetical protein	2.32	1.94E-06	Down
SS1G_05061	Hypothetical protein	2.31	2.50E-07	Down
SS1G_13264	Putative amidase	2.31	1.23E-03	Down
SS1G_14018	Oxidoreductase	2.31	1.49E-06	Down
SS1G_03164	N-acetyltransferase	2.30	3.14E-07	Down
SS1G_03161	Hypothetical protein	2.30	7.42E-09	Down
SS1G_09644	Hypothetical protein	2.30	5.81E-07	Down
SS1G_04731	Hypothetical protein	2.29	8.64E-07	Down
SS1G_00217	Hypothetical protein	2.29	3.74E-06	Down
SS1G_12634	Hypothetical protein	2.27	2.88E-07	Down

SS1G_11781	Amino acid transporter	2.27	3.09E-07	Down
SS1G_13631	Hypothetical protein	2.26	1.49E-06	Down
SS1G_09953	Hypothetical protein	2.26	3.27E-04	Down
SS1G_03162	Hypothetical protein	2.26	2.86E-04	Down
SS1G_06297	Hypothetical protein	2.25	2.02E-05	Down
SS1G_03258	Oligopeptide transporter	2.25	1.41E-06	Down
SS1G_11617	Hypothetical protein	2.25	5.68E-05	Down
SS1G_09638	Cytochrome P450 monooxygenase	2.24	3.65E-06	Down
SS1G_13511	Hypothetical protein	2.24	1.57E-04	Down
SS1G_00703	Glutathione S-transferase-like protein	2.24	3.50E-04	Down
SS1G_10503	Hypothetical protein	2.23	3.02E-05	Down
SS1G_04167	Hypothetical protein	2.23	1.21E-06	Down
SS1G_13017	Hypothetical protein	2.23	2.96E-04	Down
SS1G_08902	Hypothetical protein	2.23	3.57E-03	Down
SS1G_10559	Hypothetical protein	2.22	4.94E-08	Down
SS1G_05871	Hypothetical protein	2.22	8.27E-04	Down
SS1G_13014	Hypothetical protein	2.21	1.15E-03	Down
SS1G_10768	Hypothetical protein	2.20	1.20E-06	Down
SS1G_00702	O-methyltransferase	2.18	4.22E-03	Down
SS1G_03912	Hypothetical protein	2.17	6.92E-05	Down
SS1G_09187	Sugar transporter	2.17	5.48E-04	Down
SS1G_01081	Catalase	2.16	3.23E-07	Down
SS1G_10760	Hypothetical protein	2.16	3.00E-05	Down
SS1G_02119	FAD-linked oxidoreductase	2.16	7.63E-07	Down
SS1G_13908	Acyltransferase	2.15	7.24E-04	Down
SS1G_01482	Hypothetical protein	2.15	4.14E-05	Down
SS1G_01558	Beta-glucosidase	2.15	1.42E-06	Down
SS1G_13385	Hypothetical protein	2.14	4.22E-04	Down
SS1G_07663	Extracellular glycosidase	2.13	6.78E-07	Down
SS1G_06088	Zinc finger protein	2.13	1.23E-03	Down
SS1G_00945	Hypothetical protein	2.12	6.97E-05	Down
SS1G_10094	Hypothetical protein	2.12	6.68E-05	Down
SS1G_09647	Hypothetical protein	2.12	5.59E-04	Down
SS1G_09806	Hypothetical protein	2.12	2.46E-03	Down
SS1G_03229	Hypothetical protein	2.11	1.40E-05	Down
SS1G_00644	Methyltransferase	2.10	9.63E-05	Down
SS1G_03465	Hypothetical protein	2.08	1.13E-06	Down
SS1G_04931	L-asparaginase	2.08	8.38E-05	Down
SS1G_11431	Hypothetical protein	2.08	9.63E-07	Down
SS1G_09368	Cation transporter	2.08	1.24E-06	Down
SS1G_14127	Gamma-glutamyltransferase	2.08	3.53E-05	Down
SS1G_02083	Hypothetical protein	2.08	5.93E-04	Down
SS1G_11154	Hypothetical protein	2.08	2.67E-04	Down
SS1G_02149	Hypothetical protein	2.08	3.56E-04	Down
SS1G_09244	Hypothetical protein	2.07	1.63E-06	Down
SS1G_02317	Hypothetical protein	2.07	5.00E-06	Down
SS1G_10836	Clock-controlled protein	2.07	2.43E-05	Down

SS1G_08907	Glycoside hydrolase	2.07	3.93E-05	Down
SS1G_01109	Zinc finger protein	2.06	4.19E-04	Down
SS1G_10767	Hypothetical protein	2.05	4.59E-04	Down
SS1G_07749	Endo-1,4-beta-xylanase	2.05	4.54E-03	Down
SS1G_02763	Hypothetical protein	2.05	5.80E-04	Down
SS1G_06349	Uridine nucleosidase	2.04	5.75E-05	Down
SS1G_05463	Hypothetical protein	2.04	6.97E-06	Down
SS1G_09477	Hypothetical protein	2.04	3.39E-06	Down
SS1G_10882	Alpha-amylase	2.03	1.86E-04	Down
SS1G_12782	Hypothetical protein	2.03	4.31E-06	Down
SS1G_05140	Esterase	2.03	1.31E-03	Down
SS1G_04186	Glycosyltransferase	2.03	1.06E-05	Down
SS1G_12635	Hypothetical protein	2.03	6.08E-05	Down
SS1G_10677	Trihydroxynaphthalene reductase	2.02	2.94E-05	Down
SS1G_03774	Hypothetical protein	2.02	8.97E-05	Down
SS1G_07027	Hypothetical protein	2.02	3.17E-03	Down
SS1G_00359	Hypothetical protein	2.02	2.69E-04	Down
SS1G_04969	Hypothetical protein	2.01	4.12E-05	Down
SS1G_11119	Hypothetical protein	2.01	4.48E-04	Down
SS1G_12762	Hypothetical protein	2.01	2.05E-03	Down
SS1G_08548	Hypothetical protein	2.01	1.16E-05	Down
SS1G_11487	Hypothetical protein	2.01	1.05E-04	Down
SS1G_06703	Hypothetical protein	2.00	1.66E-03	Down
SS1G_03319	Lipase	2.00	9.08E-05	Down
SS1G_05064	Hypothetical protein	2.00	2.09E-04	Down
SS1G_07404	Hypothetical protein	2.00	8.33E-06	Down
SS1G_05812	Dehydrogenase	1.99	1.13E-04	Down
SS1G_01212	Hypothetical protein	1.99	1.73E-06	Down
SS1G_05503	Hypothetical protein	1.99	3.78E-05	Down
SS1G_06044	Hypothetical protein	1.99	1.07E-06	Down
SS1G_10436	Hypothetical protein	1.98	8.04E-07	Down
SS1G_08761	Anthranilate synthase component	1.98	6.78E-05	Down
SS1G_11181	Hypothetical protein	1.97	2.94E-03	Down
SS1G_03593	Monooxygenase	1.97	3.73E-04	Down
SS1G_08994	Hypothetical protein	1.97	2.81E-04	Down
SS1G_09541	Ankyrin repeat domain-containing protein	1.97	2.28E-03	Down
SS1G_03163	Hypothetical protein	1.97	1.98E-03	Down
SS1G_08877	Hypothetical protein	1.97	3.62E-05	Down
SS1G_00410	Hypothetical protein	1.96	6.81E-06	Down
SS1G_01291	Hypothetical protein	1.96	6.16E-05	Down
SS1G_09821	Polysaccharide monooxygenase	1.96	2.36E-03	Down
SS1G_07303	Carboxypeptidase	1.95	8.16E-06	Down
SS1G_13909	Cytochrome P450	1.95	2.28E-03	Down
SS1G_00411	Hypothetical protein	1.94	4.88E-03	Down
SS1G_13315	Dehydrogenase	1.94	1.74E-03	Down
SS1G_14433	Lyase	1.94	1.43E-05	Down
SS1G_06949	Hypothetical protein	1.94	1.32E-06	Down

SS1G_02859	Hypothetical protein	1.92	2.98E-03	Down
SS1G_07351	Hypothetical protein	1.92	1.04E-05	Down
SS1G_02140	Heterokaryon incompatibility protein S	1.91	4.99E-04	Down
SS1G_09105	Hypothetical protein	1.91	4.40E-05	Down
SS1G_06213	Hypothetical protein	1.91	7.93E-05	Down
SS1G_09765	Zinc finger protein	1.90	2.21E-05	Down
SS1G_08995	Hypothetical protein	1.90	5.11E-04	Down
SS1G_13008	Nucleic acid binding protien	1.90	1.48E-04	Down
SS1G_12180	Hypothetical protein	1.89	3.58E-06	Down
SS1G_05424	Hydroxyquinol 1,2-dioxygenase	1.89	4.03E-06	Down
SS1G_01984	Alcohol oxidase	1.89	2.79E-04	Down
SS1G_12291	Hypothetical protein	1.89	5.00E-06	Down
SS1G_02164	Aryl-alcohol dehydrogenase	1.88	4.40E-05	Down
SS1G_09954	Hypothetical protein	1.88	2.74E-03	Down
SS1G_06946	Hypothetical protein	1.87	7.84E-05	Down
SS1G_01346	Hypothetical protein	1.87	6.76E-04	Down
SS1G_11885	Hypothetical protein	1.87	8.75E-05	Down
SS1G_12499	Carboxypeptidase	1.86	8.72E-06	Down
SS1G_01036	Hypothetical protein	1.86	2.02E-04	Down
SS1G_06146	Hypothetical protein	1.85	3.16E-04	Down
SS1G_00855	Hypothetical protein	1.85	1.50E-03	Down
SS1G_01490	Hypothetical protein	1.84	3.41E-03	Down
SS1G_13594	ATPase family AAA domain-containing protein	1.84	1.08E-04	Down
SS1G_11265	Hypothetical protein	1.83	1.35E-05	Down
SS1G_03016	Monooxygenase	1.82	7.67E-04	Down
SS1G_01528	Lanosterol synthase	1.82	3.37E-05	Down
SS1G_07180	Tryprostatin hydroxylase	1.82	2.48E-05	Down
SS1G_09363	Feruloyl esterase	1.80	1.13E-04	Down
SS1G_07881	Transcription cofactor	1.79	1.86E-03	Down
SS1G_07964	Oxidoreductase	1.79	1.04E-05	Down
SS1G_13326	Hypothetical protein	1.79	6.87E-05	Down
SS1G_11922	Endo-arabinosidase	1.79	2.33E-03	Down
SS1G_12281	Hypothetical protein	1.78	3.02E-04	Down
SS1G_12468	Hypothetical protein	1.78	1.21E-03	Down
SS1G_13732	Endochitinase	1.77	5.53E-04	Down
SS1G_10306	Hypothetical protein	1.77	3.10E-05	Down
SS1G_12209	Receptor-like protein	1.77	1.49E-04	Down
SS1G_09232	Hypothetical protein	1.76	5.21E-05	Down
SS1G_08416	Hypothetical protein	1.75	4.41E-04	Down
SS1G_04366	Hypothetical protein	1.75	7.63E-04	Down
SS1G_08418	Phosphate metabolism protein	1.75	4.57E-04	Down
SS1G_07234	Cell wall integrity and stress response component	1.75	9.73E-05	Down
SS1G_10244	AP-1-like transcription activator	1.75	1.29E-04	Down
SS1G_08794	Sugar transporter	1.74	1.31E-04	Down
SS1G_09475	Carboxypeptidase	1.74	4.42E-04	Down

SS1G_03015	GTPase	1.74	1.20E-04	Down
SS1G_13133	Hypothetical protein	1.74	8.90E-04	Down
SS1G_03467	Methyltransferase	1.74	5.29E-04	Down
SS1G_03571	Hypothetical protein	1.74	5.98E-05	Down
SS1G_02242	Hypothetical protein	1.73	9.56E-04	Down
SS1G_12763	Cyanovirin-N homolog	1.73	6.01E-04	Down
SS1G_10604	Dentin sialophosphoprotein	1.73	4.32E-03	Down
SS1G_12486	Hypothetical protein	1.72	4.69E-03	Down
SS1G_12615	Hypothetical protein	1.72	1.54E-04	Down
SS1G_10607	Hypothetical protein	1.72	1.62E-03	Down
SS1G_07655	Serine protease	1.72	1.09E-03	Down
SS1G_01627	Laccase	1.71	2.86E-03	Down
SS1G_11629	UPF0613-like protei	1.71	1.36E-03	Down
SS1G_06917	Hypothetical protein	1.71	1.21E-03	Down
SS1G_14306	Homocitrate synthase, mitochondrial	1.71	3.78E-04	Down
SS1G_07166	Stress response protein	1.70	3.46E-03	Down
SS1G_06536	Amino-acid transporter	1.70	1.47E-03	Down
SS1G_10457	Hypothetical protein	1.70	1.07E-03	Down
SS1G_05304	Aromatic peroxygenase	1.68	2.86E-03	Down
SS1G_11320	Altered inheritance of mitochondria protein	1.68	3.45E-04	Down
SS1G_06174	Hypothetical protein	1.68	3.92E-04	Down
SS1G_13516	Hypothetical protein	1.67	8.17E-05	Down
SS1G_09225	Serine protease	1.67	2.14E-04	Down
SS1G_01614	Opsin-1	1.65	4.67E-03	Down
SS1G_04295	Hypothetical protein	1.64	2.88E-04	Down
SS1G_01480	Dehydrogenase	1.64	2.28E-03	Down
SS1G_08677	Cytochrome P450 monooxygenase	1.63	3.43E-04	Down
SS1G_01651	Transporter	1.63	8.72E-04	Down
SS1G_14525	Transporter	1.63	4.01E-04	Down
SS1G_05308	Hypothetical protein	1.63	1.59E-03	Down
SS1G_08861	Hypothetical protein	1.63	2.78E-04	Down
SS1G_12938	Cell wall protein	1.63	2.25E-03	Down
SS1G_07278	Hypothetical protein	1.62	7.22E-04	Down
SS1G_10705	Psi-producing oxygenase	1.62	3.81E-03	Down
SS1G_03438	Fatty acid desaturase	1.62	1.81E-03	Down
SS1G_10502	Rap guanine nucleotide exchange factor	1.61	6.10E-04	Down
SS1G_10707	Cell adhesion molecule	1.61	3.46E-03	Down
SS1G_04138	Mutanase	1.61	5.97E-04	Down
SS1G_02845	Superoxide dismutase	1.60	1.66E-03	Down
SS1G_09929	Acyl-carrier protein	1.59	3.31E-03	Down
SS1G_03459	Hypothetical protein	1.59	2.03E-03	Down
SS1G_05918	Hypothetical protein	1.59	1.11E-03	Down
SS1G_09114	Chaperone protein DnaJ	1.58	3.08E-03	Down
SS1G_06904	Monooxygenase	1.58	2.86E-03	Down
SS1G_04907	mRNA export factor	1.58	9.47E-04	Down
SS1G_11224	High affinity cysteine transporter	1.58	1.23E-03	Down
SS1G_13838	RNA binding protein	1.57	4.58E-03	Down

SS1G_05388	Hypothetical protein	1.57	4.01E-04	Down
SS1G_12810	Hypothetical protein	1.57	3.04E-03	Down
SS1G_11596	Hypothetical protein	1.56	9.42E-04	Down
SS1G_10263	Non-motor actin binding protein	1.56	1.95E-03	Down
SS1G_01425	Hypothetical protein	1.55	1.38E-03	Down
SS1G_12822	Hypothetical protein	1.53	4.81E-04	Down
SS1G_01937	Hypothetical protein	1.53	2.75E-03	Down
SS1G_02947	Hypothetical protein	1.52	3.87E-03	Down
SS1G_02646	Hypothetical protein	1.52	2.57E-03	Down
SS1G_10320	Hypothetical protein	1.52	1.15E-03	Down
SS1G_04984	Hypothetical protein	1.52	1.10E-03	Down
SS1G_11212	Polysaccharide monooxygenase	1.52	2.51E-03	Down
SS1G_14001	Fatty acid oxidation complex subunit	1.52	2.19E-03	Down
SS1G_13638	Efflux pump	1.50	2.22E-03	Down
SS1G_13812	Oxidoreductase	1.49	3.50E-03	Down
SS1G_07962	Hypothetical protein	1.49	1.01E-03	Down
SS1G_03014	Hypothetical protein	1.49	4.14E-03	Down
SS1G_06143	Hypothetical protein	1.49	1.36E-03	Down
SS1G_06041	Transcription elongation factor	1.49	4.77E-03	Down
SS1G_09655	Hypothetical protein	1.48	2.17E-03	Down
SS1G_08132	Transmembrane protein	1.48	6.33E-04	Down
SS1G_03642	Oxidoreductase	1.47	3.30E-03	Down
SS1G_01840	Hypothetical protein	1.47	4.05E-03	Down
SS1G_03292	Hypothetical protein	1.47	1.82E-03	Down
SS1G_00810	TLC domain-containing protein C17A2.02c	1.46	3.06E-03	Down
SS1G_00824	Clumping factor B	1.46	4.52E-03	Down
SS1G_07892	Hypothetical protein	1.46	2.23E-03	Down
SS1G_13559	Anion/proton exchange transporter	1.44	2.70E-03	Down
SS1G_01811	Dehydrogenase	1.44	2.76E-03	Down
SS1G_10305	Thioredoxin-like protein	1.44	4.81E-03	Down
SS1G_05489	Hypothetical protein	1.43	3.97E-03	Down
SS1G_00267	Ubiquitin-protein ligase	1.43	1.30E-03	Down
SS1G_12536	Hypothetical protein	1.43	1.34E-03	Down
SS1G_07901	Metalloprotease(PC00121)	1.41	3.42E-03	Down
SS1G_09944	Long-chain-fatty-acid--CoA ligase 1	1.41	1.18E-03	Down
SS1G_05062	Hypothetical protein	1.40	3.62E-03	Down
SS1G_12801	Coumarate--CoA ligase	1.40	1.97E-03	Down
SS1G_07015	Hypothetical protein	1.39	3.56E-03	Down
SS1G_07698	ATP-dependent DNA helicase	1.39	3.18E-03	Down
SS1G_12535	Hypothetical protein	1.37	2.08E-03	Down
SS1G_13131	Hypothetical protein	1.37	2.18E-03	Down
SS1G_14421	Phospholipase	1.35	4.69E-03	Down
SS1G_06441	Hypothetical protein	1.34	4.66E-03	Down
SS1G_05586	Hypothetical protein	1.31	4.73E-03	Down
SS1G_06222	Fumarate reductase	1.29	3.46E-03	Down

Table S2b. Trial #2 coding regions differentially expressed between 5 virus-free and 5 hypovirus-infected cultures

Locus	Annotation	Log 2 fold change	adj-P-value	Up/Down
SS1G_09248	Cryparin	4.419	6.67E-21	Up
SS1G_13965	Hypothetical protein	4.248	1.55E-11	Up
SS1G_14450	Oxygenase	4.045	2.30E-12	Up
SS1G_09241	Hypothetical protein	3.952	1.33E-09	Up
SS1G_11753	Aflatoxin B1 aldehyde reductase	3.924	1.89E-14	Up
SS1G_10129	Hypothetical protein	3.712	1.27E-07	Up
SS1G_09242	Hypothetical protein	3.605	1.09E-07	Up
SS1G_12301	Hypothetical protein	3.534	1.41E-10	Up
SS1G_05491	Oxygenase	3.502	1.92E-09	Up
SS1G_14451	Hypothetical protein	3.401	4.71E-09	Up
SS1G_09374	Oxidoreductase	3.389	1.57E-13	Up
SS1G_14452	Hypothetical protein	3.353	1.51E-06	Up
SS1G_14003	Hypothetical protein	3.338	1.49E-06	Up
SS1G_09238	Hydroxylase	3.330	1.00E-09	Up
SS1G_09236	Hypothetical protein	3.272	2.98E-10	Up
SS1G_01199	Pyridoxal reductase	3.241	5.06E-13	Up
SS1G_13633	Serine protease	3.219	7.03E-12	Up
SS1G_05223	Methyltransferase	3.169	1.00E-09	Up
SS1G_09135	NADP-dependent alcohol dehydrogenase	3.168	1.09E-11	Up
SS1G_13635	Hypothetical protein	3.118	5.94E-12	Up
SS1G_12712	Hypothetical protein	3.077	5.22E-08	Up
SS1G_09234	Oxygenase	3.071	2.11E-10	Up
SS1G_06132	Dehydrogenase	3.068	3.80E-08	Up
SS1G_04465	Dehydrogenase/reductase	3.029	3.20E-13	Up
SS1G_12713	Hypothetical protein	2.973	2.88E-05	Up
SS1G_02828	Hypothetical protein	2.950	3.12E-18	Up
SS1G_11498	Choline-sulfatase	2.902	1.41E-14	Up
SS1G_13637	Hypothetical protein	2.895	1.02E-12	Up
SS1G_09244	Hypothetical protein	2.895	5.19E-09	Up
SS1G_00884	Dehydrogenase	2.850	1.51E-14	Up
SS1G_09239	Hypothetical protein	2.831	1.20E-07	Up
SS1G_03247	Transporter	2.817	1.80E-07	Up
SS1G_09243	Hypothetical protein	2.792	9.86E-08	Up
SS1G_07222	Short-chain dehydrogenase	2.786	6.55E-07	Up
SS1G_11540	Hypothetical protein	2.747	3.71E-04	Up
SS1G_12300	Oxygenase	2.654	1.33E-05	Up
SS1G_09235	Hydroxylase	2.592	1.51E-11	Up
SS1G_11539	Terpene cyclase	2.555	4.60E-08	Up
SS1G_04464	Hypothetical protein	2.549	2.96E-15	Up
SS1G_09237	Polyketide synthase 5-related protein	2.549	9.78E-06	Up
SS1G_13642	Transporter	2.536	3.07E-13	Up
SS1G_04468	Alpha-1,2-mannosidase	2.521	1.75E-19	Up
SS1G_13641	Polyketide synthase 5-related protein	2.502	3.60E-07	Up

SS1G_13636	Cyclochlorotine biosynthesis protein	2.475	1.27E-08	Up
SS1G_01489	Hypothetical protein	2.458	1.00E-08	Up
SS1G_11932	Dehydrogenase	2.457	1.60E-10	Up
SS1G_10128	Hypothetical protein	2.437	1.00E-03	Up
SS1G_02194	Oxygenase	2.435	1.10E-09	Up
SS1G_07655	Serine protease	2.420	1.76E-09	Up
SS1G_06056	Methyltransferase	2.418	5.71E-04	Up
SS1G_09858	Hypothetical protein	2.415	2.72E-06	Up
SS1G_00044	Hypothetical protein	2.381	2.13E-13	Up
SS1G_13877	Protein AIM2	2.367	1.98E-08	Up
SS1G_13844	Hypothetical protein	2.336	3.92E-04	Up
SS1G_04945	Glucanase	2.326	3.32E-09	Up
SS1G_07854	Acetyltransferase	2.320	3.63E-10	Up
SS1G_13264	Putative amidase	2.320	2.67E-03	Up
SS1G_016208	Hypothetical protein	2.308	2.58E-03	Up
SS1G_09270	Hypothetical protein	2.280	2.47E-11	Up
SS1G_02454	Aflatoxin B1 aldehyde reductase member	2.268	4.38E-07	Up
SS1G_03644	Hypothetical protein	2.262	3.52E-04	Up
SS1G_00148	Versiconal hemiacetal acetate reductase	2.261	7.54E-15	Up
SS1G_07836	Hypothetical protein	2.256	2.77E-04	Up
SS1G_12210	Serine protease	2.236	9.34E-06	Up
SS1G_09225	Serine protease	2.219	4.87E-07	Up
SS1G_06401	Glucosidase	2.192	2.33E-06	Up
SS1G_06037	Glucan 1,3-beta-glucosidase	2.154	1.00E-09	Up
SS1G_13683	Fatty acid desaturase	2.139	2.01E-05	Up
SS1G_06448	Retinol dehydrogenase	2.134	1.40E-05	Up
SS1G_12907	Cutinase	2.119	3.42E-05	Up
SS1G_13554	Acetyltransferase	2.119	2.57E-09	Up
SS1G_05974	Amino acid transporter	2.118	1.08E-04	Up
SS1G_06370	Hypothetical protein	2.110	2.27E-06	Up
SS1G_08384	Hypothetical protein	2.108	4.87E-07	Up
SS1G_12062	Methylsalicylic acid decarboxylase	2.108	6.19E-05	Up
SS1G_015697	Hypothetical protein	2.107	7.38E-03	Up
SS1G_05912	Hypothetical protein	2.107	2.41E-05	Up
SS1G_12116	Didemethylasterriquinone D synthetase	2.079	1.28E-03	Up
SS1G_13639	Reductase	2.062	5.52E-06	Up
SS1G_02607	Aromatic-l-amino-acid decarboxylase	2.043	1.81E-15	Up
SS1G_02161	Oxygenase	2.016	6.26E-05	Up
SS1G_12142	Sugar transport protein	2.001	9.58E-06	Up
SS1G_14293	Glucose oxidase	1.998	6.71E-05	Up
SS1G_13386	Cutinase	1.998	4.12E-05	Up
SS1G_05794	Hypothetical protein	1.975	2.80E-11	Up
SS1G_14166	Hypothetical protein	1.975	1.17E-05	Up
SS1G_01572	Beta-galactosidase	1.957	1.45E-03	Up
SS1G_11629	Upf0613-like protei	1.943	9.46E-06	Up
SS1G_06068	Hypothetical protein	1.934	9.46E-06	Up
SS1G_12708	Cytochrome P450 monooxygenase	1.924	2.76E-04	Up

SS1G_12198	Dehydrogenase	1.923	6.42E-04	Up
SS1G_04819	Serine protease	1.906	5.53E-07	Up
SS1G_00858	Hypothetical protein	1.904	1.99E-06	Up
SS1G_01107	Hypothetical protein	1.901	2.69E-15	Up
SS1G_05784	Serine protease	1.899	2.00E-09	Up
SS1G_12870	Glutathione-dependent formaldehyde-activat	1.893	2.06E-06	Up
SS1G_00458	Hypothetical protein	1.890	1.99E-06	Up
SS1G_12817	Sulfhydryl oxidase	1.889	6.64E-09	Up
SS1G_05493	Carboxylic ester hydrolase	1.888	2.39E-03	Up
SS1G_02756	Hypothetical protein	1.886	1.10E-05	Up
SS1G_11269	Lyase	1.867	1.50E-06	Up
SS1G_02738	Dehydrogenase	1.862	1.98E-04	Up
SS1G_02695	Hypothetical protein	1.856	2.60E-03	Up
SS1G_01389	Hypothetical protein	1.854	3.02E-05	Up
SS1G_10263	Non-motor actin binding protein	1.837	1.54E-06	Up
SS1G_06394	Guanyl-specific ribonuclease	1.837	4.79E-12	Up
SS1G_03361	Serine protease	1.829	1.99E-05	Up
SS1G_13362	Amidohydrolase	1.828	6.20E-06	Up
SS1G_03404	Dehydrogenase	1.816	3.73E-08	Up
SS1G_01491	Reductase	1.813	7.43E-05	Up
SS1G_02042	ATP-binding cassette (ABC) transporter	1.813	2.80E-04	Up
SS1G_11212	Polysaccharide monooxygenase	1.803	4.50E-05	Up
SS1G_02336	Dehydrogenase	1.798	3.58E-10	Up
SS1G_13862	Hypothetical protein	1.794	3.89E-03	Up
SS1G_13582	Hypothetical protein	1.792	9.73E-04	Up
SS1G_02115	Hypothetical protein	1.792	7.60E-05	Up
SS1G_11418	Ligase	1.784	1.12E-05	Up
SS1G_04343	Hypothetical protein	1.775	5.65E-04	Up
SS1G_10561	Hypothetical protein	1.774	3.16E-06	Up
SS1G_03346	Hypothetical protein	1.770	1.54E-04	Up
SS1G_03181	Aspartic protease	1.769	1.53E-05	Up
SS1G_06143	Hypothetical protein	1.761	1.31E-06	Up
SS1G_03465	Hypothetical protein	1.758	1.98E-06	Up
SS1G_00601	Hypothetical protein	1.755	2.81E-04	Up
SS1G_10559	Hypothetical protein	1.742	2.10E-06	Up
SS1G_03393	Ethanolamine utilization protein	1.741	1.26E-05	Up
SS1G_02946	Hypothetical protein	1.737	2.34E-09	Up
SS1G_12824	Hypothetical protein	1.734	6.42E-11	Up
SS1G_00147	Lyase	1.724	2.66E-11	Up
SS1G_03402	Hydrolase	1.723	9.36E-05	Up
SS1G_01387	Hypothetical protein	1.723	2.36E-09	Up
SS1G_09771	Dehydrogenase/reductase	1.720	6.08E-07	Up
SS1G_12075	Oxidase	1.717	3.58E-04	Up
SS1G_00337	Hypothetical protein	1.712	1.20E-05	Up
SS1G_09105	Hypothetical protein	1.709	4.76E-04	Up
SS1G_02030	Hypothetical protein	1.703	1.08E-10	Up
SS1G_07294	Decarboxylase	1.695	1.46E-13	Up

SS1G_05788	Hypothetical protein	1.694	5.82E-05	Up
SS1G_11329	Hypothetical protein	1.694	5.35E-05	Up
SS1G_05515	Hypothetical protein	1.664	6.91E-04	Up
SS1G_11729	Dehydrogenase	1.656	2.80E-04	Up
SS1G_08695	Endochitinase	1.656	7.99E-06	Up
SS1G_12782	Hypothetical protein	1.641	2.11E-03	Up
SS1G_12821	Proteasome endopeptidase complex	1.640	7.95E-12	Up
SS1G_09163	Hypothetical protein	1.637	2.31E-06	Up
SS1G_08889	Hypothetical protein	1.634	2.01E-04	Up
SS1G_14244	Probable efflux pump protein	1.628	2.92E-03	Up
SS1G_06040	Lipase	1.624	5.59E-06	Up
SS1G_06079	L-serine dehydratase	1.618	6.58E-06	Up
SS1G_12413	Carboxypeptidase	1.614	1.58E-05	Up
SS1G_04552	Hypothetical protein	1.606	6.84E-03	Up
SS1G_12040	Hypothetical protein	1.603	1.98E-03	Up
SS1G_02046	Hypothetical protein	1.603	3.24E-03	Up
SS1G_12816	Amino-acid aminotransferase	1.596	4.73E-03	Up
SS1G_06035	Dehydrogenase	1.593	2.53E-08	Up
SS1G_08671	Hypothetical protein	1.584	9.49E-08	Up
SS1G_09134	Hypothetical protein	1.584	7.92E-03	Up
SS1G_13346	Oligopeptide transporter 1	1.583	6.19E-03	Up
SS1G_07479	Acetyltransferase	1.579	1.01E-04	Up
SS1G_05574	Acetyltransferase	1.578	6.81E-09	Up
SS1G_09365	Hypothetical protein	1.568	1.68E-08	Up
SS1G_09240	Acyltransferase	1.568	3.31E-05	Up
SS1G_00332	Pectinesterase	1.564	4.18E-04	Up
SS1G_01627	Iron transport multicopper oxidase	1.564	5.96E-05	Up
SS1G_13923	Cytochrome P450 monooxygenase	1.557	6.83E-05	Up
SS1G_03518	Serine protease	1.554	1.86E-05	Up
SS1G_07630	4-hydroxyphenylpyruvate dioxygenase	1.552	4.06E-04	Up
SS1G_05556	Transporter	1.535	1.46E-05	Up
SS1G_03553	Hypothetical protein	1.532	1.38E-03	Up
SS1G_01558	Beta-glucosidase	1.530	1.24E-04	Up
SS1G_01099	Dehydrogenase	1.530	3.73E-04	Up
SS1G_02218	2-oxoisovalerate dehydrogenase subunit alph	1.521	7.86E-04	Up
SS1G_03067	Hypothetical protein	1.512	2.47E-04	Up
SS1G_08643	Hypothetical protein	1.511	1.54E-06	Up
SS1G_01478	Sphingolipid C9-methyltransferase	1.507	6.97E-08	Up
SS1G_02068	Hypothetical protein	1.507	6.20E-03	Up
SS1G_10919	Methyltransferase	1.504	2.19E-05	Up
SS1G_13860	Endoglucanase	1.498	1.29E-04	Up
SS1G_09823	Hypothetical protein	1.493	3.63E-04	Up
SS1G_02693	Hypothetical protein	1.491	7.84E-03	Up
SS1G_09993	Hypothetical protein	1.486	1.57E-05	Up
SS1G_07015	Hypothetical protein	1.486	3.02E-08	Up
SS1G_10746	Hypothetical protein	1.480	1.25E-03	Up
SS1G_03226	Ubiquitin-protein ligase	1.479	2.87E-07	Up

SS1G_12818	Protease	1.479	3.37E-13	Up
SS1G_03245	Hypothetical protein	1.478	7.33E-04	Up
SS1G_07180	Tryprostatin hydroxylase	1.471	4.46E-03	Up
SS1G_10167	Endo-polygalacturonase	1.467	1.74E-03	Up
SS1G_11414	Dehydrogenase	1.464	1.36E-03	Up
SS1G_12084	Hypothetical protein	1.463	6.29E-05	Up
SS1G_05787	Non-reducing polyketide synthase	1.461	5.46E-04	Up
SS1G_12526	Cation transporter	1.453	2.15E-04	Up
SS1G_05195	Serine protease	1.446	3.32E-03	Up
SS1G_01984	Alcohol oxidase	1.440	2.90E-03	Up
SS1G_08672	Hypothetical protein	1.439	4.98E-08	Up
SS1G_08095	Hypothetical protein	1.437	9.94E-07	Up
SS1G_13355	Phenylalanine aminomutase	1.436	3.45E-06	Up
SS1G_10295	Glutathione S-transferase 2	1.435	4.29E-09	Up
SS1G_11988	Glucose dehydrogenase	1.425	4.08E-03	Up
SS1G_03309	Hypothetical protein	1.424	5.12E-12	Up
SS1G_04362	Hypothetical protein	1.421	4.92E-03	Up
SS1G_08441	Biotin synthase, mitochondrial	1.421	1.11E-09	Up
SS1G_11670	Hypothetical protein	1.414	9.69E-03	Up
SS1G_01802	Amino acid transporter	1.413	3.96E-03	Up
SS1G_08563	Reductase	1.413	4.65E-03	Up
SS1G_09997	Hypothetical protein	1.403	4.63E-03	Up
SS1G_10691	Hypothetical protein	1.401	4.22E-03	Up
SS1G_05381	Hypothetical protein	1.401	1.25E-03	Up
SS1G_13812	Oxidoreductase	1.400	9.69E-03	Up
SS1G_06477	Hypothetical protein	1.399	7.55E-07	Up
SS1G_03843	Hypothetical protein	1.399	1.66E-06	Up
SS1G_00121	Hypothetical protein	1.397	3.44E-03	Up
SS1G_11362	Methyltransferase	1.396	6.91E-04	Up
SS1G_08706	Hypothetical protein	1.395	1.90E-05	Up
SS1G_09768	Hypothetical protein	1.395	4.13E-20	Up
SS1G_01346	Hypothetical protein	1.389	1.77E-03	Up
SS1G_12359	Groes-like zinc-binding alcohol dehydrogenas	1.389	1.58E-04	Up
SS1G_10035	Transaminase	1.388	2.26E-04	Up
SS1G_09978	Peptidase	1.387	8.84E-03	Up
SS1G_05932	NADH-cytochrome b5 reductase 2	1.387	1.03E-15	Up
SS1G_09446	Acyltransferase	1.385	8.68E-03	Up
SS1G_04310	Phosphoadenosine phosphosulfate reductase	1.373	4.65E-03	Up
SS1G_04614	Oxidoreductase	1.371	1.86E-05	Up
SS1G_06688	Dihydrolipoamide acetyltransferase	1.363	2.92E-03	Up
SS1G_08257	Glutamine amidotransferase	1.357	1.56E-03	Up
SS1G_04671	Hypothetical protein	1.354	1.38E-03	Up
SS1G_03034	Centromere protein V	1.351	7.82E-04	Up
SS1G_09020	Glucanase	1.350	6.65E-03	Up
SS1G_015885	Hypothetical protein	1.346	1.93E-03	Up
SS1G_08163	Hypothetical protein	1.340	9.70E-03	Up
SS1G_13922	Tripeptidyl-peptidase	1.339	7.92E-04	Up

SS1G_11102	Hypothetical protein	1.338	2.84E-03	Up
SS1G_04137	Hypothetical protein	1.333	6.44E-04	Up
SS1G_11367	Transcriptional activator	1.328	3.59E-03	Up
SS1G_04888	Methyltransferase-like protein	1.322	3.87E-06	Up
SS1G_00735	Hypothetical protein	1.319	5.96E-04	Up
SS1G_14167	Hypothetical protein	1.316	5.41E-05	Up
SS1G_14168	Hydroxylase	1.315	5.12E-05	Up
SS1G_00481	Hypothetical protein	1.314	4.16E-04	Up
SS1G_11066	Hypothetical protein	1.304	1.30E-08	Up
SS1G_01871	Putative thiosulfate sulfurtransferase	1.297	4.94E-06	Up
SS1G_01479	Glutathione S-transferase	1.294	4.06E-04	Up
SS1G_04413	Transmembrane protein	1.294	2.52E-08	Up
SS1G_04817	Hypothetical protein	1.292	6.90E-07	Up
SS1G_00237	Acetyltransferase	1.292	4.83E-04	Up
SS1G_12873	Hypothetical protein	1.289	1.13E-10	Up
SS1G_08368	Damaged DNA-binding protein	1.285	1.07E-03	Up
SS1G_05578	Copper transporter	1.281	2.40E-04	Up
SS1G_05273	Hydrolase	-1.280	7.19E-04	Down
SS1G_07179	Hypothetical protein	-1.283	7.52E-03	Down
SS1G_12930	Hypothetical protein	-1.283	7.31E-03	Down
SS1G_13850	Cyclochlorotine biosynthesis protein	-1.291	2.55E-05	Down
SS1G_03658	60s ribosomal export protein	-1.291	6.20E-06	Down
SS1G_06472	Hypothetical protein	-1.293	1.22E-03	Down
SS1G_07977	Hypothetical protein	-1.293	1.74E-03	Down
SS1G_06409	Hypothetical protein	-1.294	9.15E-06	Down
SS1G_12767	Ribosome biogenesis regulatory protein	-1.297	7.20E-04	Down
SS1G_06596	Nucleic acid binding	-1.298	8.75E-04	Down
SS1G_05888	Hypothetical protein	-1.300	5.61E-03	Down
SS1G_13545	NADH dehydrogenase [ubiquinone] 1 alpha	-1.301	3.38E-03	Down
SS1G_00635	Hypothetical protein	-1.301	2.33E-06	Down
SS1G_14098	DNA repair protein	-1.303	6.53E-03	Down
SS1G_01062	Amino-acid permease	-1.303	5.32E-04	Down
SS1G_06725	GPN-loop GTPase 1	-1.304	4.16E-04	Down
SS1G_03057	Multifunctional methyltransferase subunit	-1.305	4.04E-06	Down
SS1G_08136	Oxygenase	-1.308	4.34E-03	Down
SS1G_05011	U3 small nucleolar ribonucleoprotein protein	-1.309	2.77E-05	Down
SS1G_12483	Fibrillarin	-1.311	2.93E-04	Down
SS1G_02326	Hypothetical protein	-1.313	5.61E-03	Down
SS1G_06430	Hypothetical protein	-1.314	4.03E-04	Down
SS1G_05628	Hypothetical protein	-1.315	6.03E-04	Down
SS1G_02608	Tyrosine--tRNA ligase	-1.315	1.19E-03	Down
SS1G_01124	non-receptor serine/threonine protein kinase	-1.316	6.27E-07	Down
SS1G_04686	Phosphate transporter	-1.317	5.41E-04	Down
SS1G_00623	Oxygenase	-1.319	6.28E-04	Down
SS1G_00222	rRNA biogenesis protein rrp36	-1.325	2.99E-03	Down
SS1G_02882	Alternative oxidase, mitochondrial	-1.327	2.05E-07	Down
SS1G_04913	Basic leucine zipper transcription factor	-1.330	4.02E-04	Down

SS1G_02281	Phosphoenolpyruvate carboxykinase	-1.332	5.84E-03	Down
SS1G_01662	Hypothetical protein	-1.334	4.25E-03	Down
SS1G_06664	Purine-cytosine permease FCY21	-1.335	2.24E-03	Down
SS1G_05968	Hypothetical protein	-1.335	2.23E-03	Down
SS1G_04926	Eukaryotic translation initiation factor 3G	-1.336	1.83E-03	Down
SS1G_09723	Hypothetical protein	-1.340	9.01E-04	Down
SS1G_10276	Hypothetical protein	-1.340	4.78E-07	Down
SS1G_05606	Hypothetical protein	-1.342	1.84E-04	Down
SS1G_09783	KRR1 small subunit processome component	-1.342	4.47E-04	Down
SS1G_03561	Glutamate-rich WD repeat-containing proteir	-1.344	1.73E-03	Down
SS1G_06164	Hypothetical protein	-1.344	1.78E-04	Down
SS1G_05358	GRPE protein homolog	-1.345	1.24E-04	Down
SS1G_02712	Hypothetical protein	-1.346	8.77E-03	Down
SS1G_00058	ATP-dependent RNA helicase dbp8	-1.348	2.39E-04	Down
SS1G_04442	Hypothetical protein	-1.351	9.73E-04	Down
SS1G_00789	Nucleolar protein 16	-1.358	1.98E-04	Down
SS1G_00988	Hypothetical protein	-1.361	3.52E-04	Down
SS1G_07973	Oxygenase	-1.363	3.07E-06	Down
SS1G_08406	Hypothetical protein	-1.365	5.22E-03	Down
SS1G_08048	Hypothetical protein	-1.367	3.49E-03	Down
SS1G_13926	39S ribosomal protein L54	-1.368	9.43E-05	Down
SS1G_07146	Beta-glucosidase	-1.372	1.38E-03	Down
SS1G_07872	Ribosome biogenesis protein Nop10	-1.373	4.30E-04	Down
SS1G_12895	YTH domain-containing protein	-1.374	8.63E-07	Down
SS1G_05629	ATP-dependent RNA helicase ded1	-1.375	2.84E-04	Down
SS1G_06487	Mitochondrial carrier protein	-1.375	1.91E-04	Down
SS1G_11882	Exosome component 10	-1.376	4.88E-03	Down
SS1G_00862	Annexin	-1.378	9.00E-03	Down
SS1G_06721	Hypothetical protein	-1.379	2.94E-04	Down
SS1G_10185	Hypothetical protein	-1.381	1.22E-05	Down
SS1G_12388	Hypothetical protein	-1.382	6.37E-13	Down
SS1G_07815	Hypothetical protein	-1.383	1.80E-04	Down
SS1G_08616	Hypothetical protein	-1.385	2.87E-03	Down
SS1G_14249	Inner membrane protein yidh	-1.387	1.42E-03	Down
SS1G_01272	Arginine N-methyltransferase 2	-1.389	1.15E-03	Down
SS1G_08688	Acyltransferase	-1.389	5.35E-05	Down
SS1G_01824	Px domain-containing protein	-1.390	7.15E-06	Down
SS1G_07591	Late secretory pathway protein	-1.391	4.80E-07	Down
SS1G_01143	Alpha/beta-hydrolases protein	-1.392	1.09E-03	Down
SS1G_03690	Hypothetical protein	-1.392	1.42E-04	Down
SS1G_11475	RNA binding protein	-1.394	6.59E-05	Down
SS1G_00822	Hypothetical protein	-1.394	2.87E-07	Down
SS1G_09987	Calcium-binding protein	-1.394	2.36E-04	Down
SS1G_13479	Hypothetical protein	-1.394	5.32E-04	Down
SS1G_09570	RNA helicase	-1.395	1.45E-04	Down
SS1G_11824	Ribonucleoprotein	-1.396	1.23E-04	Down
SS1G_07525	Elongator complex protein 3	-1.397	6.85E-05	Down

SS1G_11230	Transaminase	-1.398	9.09E-07	Down
SS1G_04907	Elongation factor 3	-1.398	1.32E-04	Down
SS1G_12259	Hypothetical protein	-1.399	5.41E-04	Down
SS1G_12174	Transferase	-1.399	2.03E-04	Down
SS1G_11556	Hypothetical protein	-1.399	9.95E-04	Down
SS1G_08825	Centromere DNA-binding protein	-1.399	3.09E-05	Down
SS1G_08245	Hypothetical protein	-1.399	8.26E-03	Down
SS1G_07962	Hypothetical protein	-1.400	6.89E-06	Down
SS1G_02271	Annexin	-1.400	2.00E-05	Down
SS1G_10727	Hypothetical protein	-1.401	2.38E-04	Down
SS1G_11257	Hypothetical protein	-1.401	9.95E-05	Down
SS1G_11879	Hypothetical protein	-1.404	4.42E-05	Down
SS1G_00849	Hypothetical protein	-1.404	1.20E-03	Down
SS1G_09604	DNA-directed RNA polymerase subunit	-1.407	8.51E-04	Down
SS1G_06674	Ribosome biogenesis protein erb1	-1.409	6.80E-05	Down
SS1G_00144	Cytoplasmic tRNA 2-thiolation protein 1	-1.409	2.95E-04	Down
SS1G_12054	Inosine-5'-monophosphate dehydrogenase	-1.413	9.43E-07	Down
SS1G_10006	Dehydratase	-1.415	6.65E-05	Down
SS1G_04628	Hydrolase	-1.415	4.89E-04	Down
SS1G_10436	Hypothetical protein	-1.416	3.49E-03	Down
SS1G_09801	Hypothetical protein	-1.416	1.26E-03	Down
SS1G_10815	U3 small nucleolar RNA-associated protein 25	-1.417	5.97E-04	Down
SS1G_03772	Hypothetical protein	-1.418	4.50E-05	Down
SS1G_09266	Hypothetical protein	-1.421	1.67E-03	Down
SS1G_08518	Hypothetical protein	-1.422	4.39E-05	Down
SS1G_11028	Oxysterol-binding protein-related protein	-1.423	2.91E-04	Down
SS1G_13852	ABC transporter B family member	-1.424	1.90E-03	Down
SS1G_05804	Hypothetical protein	-1.424	3.17E-04	Down
SS1G_02890	Hypothetical protein	-1.425	9.90E-03	Down
SS1G_01888	Hypothetical protein	-1.426	6.60E-08	Down
SS1G_03517	Hypothetical protein	-1.428	5.61E-03	Down
SS1G_13509	RNA binding protein	-1.431	1.75E-05	Down
SS1G_12118	Hypothetical protein	-1.435	8.29E-03	Down
SS1G_13519	Methyltransferase	-1.436	9.74E-04	Down
SS1G_08914	tRNA pseudouridine synthase A	-1.436	2.11E-03	Down
SS1G_08606	Serine/threonine-protein kinase	-1.436	2.18E-04	Down
SS1G_11897	Nucleic acid binding	-1.440	7.33E-04	Down
SS1G_14485	P53-like transcription factor	-1.440	3.75E-07	Down
SS1G_13366	DNA-directed RNA polymerase subunit beta	-1.441	1.37E-04	Down
SS1G_06496	Aldehyde reductase i	-1.441	6.72E-03	Down
SS1G_09681	60S ribosome subunit biogenesis protein NIP7	-1.442	2.84E-04	Down
SS1G_07456	Hypothetical protein	-1.445	2.41E-03	Down
SS1G_03215	tRNA threonylcarbamoyladenosine dehydrata	-1.446	3.08E-04	Down
SS1G_04695	Nitrosoguanidine resistance protein	-1.448	2.19E-04	Down
SS1G_05101	60S ribosome subunit biogenesis protein NIP7	-1.449	4.25E-04	Down
SS1G_14136	Hypothetical protein	-1.449	4.44E-04	Down
SS1G_12151	RNA cytidine acetyltransferase	-1.450	4.04E-04	Down

SS1G_01880	DNA-directed dna polymerase	-1.452	8.33E-03	Down
SS1G_01719	RAN GTPase-activating protein 1	-1.453	1.60E-10	Down
SS1G_05012	U3 small nucleolar ribonucleoprotein protein	-1.454	6.95E-05	Down
SS1G_14381	Amino acid transporter	-1.454	9.61E-04	Down
SS1G_02004	Cysteine protease	-1.456	5.53E-04	Down
SS1G_14530	Hypothetical protein	-1.457	1.02E-03	Down
SS1G_13702	Amino acid transporter	-1.457	3.43E-06	Down
SS1G_05206	Hypothetical protein	-1.459	8.61E-05	Down
SS1G_01895	Hypothetical protein	-1.459	3.98E-05	Down
SS1G_07305	ARF3-interacting protein 1	-1.460	2.64E-04	Down
SS1G_10567	Sphingolipid c4-hydroxylase	-1.461	2.61E-03	Down
SS1G_02554	Transferase	-1.463	9.21E-05	Down
SS1G_07156	Hypothetical protein	-1.463	1.73E-03	Down
SS1G_04968	Hypothetical protein	-1.463	1.77E-04	Down
SS1G_05175	Hypothetical protein	-1.463	2.46E-03	Down
SS1G_10760	Hypothetical protein	-1.463	2.43E-03	Down
SS1G_00989	Cytochrome C	-1.464	2.61E-06	Down
SS1G_03089	G-protein coupled receptor	-1.464	2.68E-03	Down
SS1G_04850	Mannan endo-1,6-alpha-mannosidase	-1.464	1.18E-07	Down
SS1G_02468	ATP-dependent RNA helicase mak5	-1.465	1.09E-03	Down
SS1G_14302	Hypothetical protein	-1.466	3.10E-05	Down
SS1G_10591	Hypothetical protein	-1.468	8.09E-05	Down
SS1G_01700	Hypothetical protein	-1.468	1.46E-13	Down
SS1G_09971	Hypothetical protein	-1.469	1.45E-04	Down
SS1G_08553	Cysteine protease	-1.469	7.32E-06	Down
SS1G_09727	Thiamine thiazole synthase	-1.470	1.01E-03	Down
SS1G_02810	Hypothetical protein	-1.470	6.25E-05	Down
SS1G_05438	Assembly factor cbp4	-1.472	8.49E-05	Down
SS1G_02863	Hypothetical protein	-1.472	1.84E-04	Down
SS1G_08319	Heat repeat-containing protein 3	-1.472	2.20E-05	Down
SS1G_01769	Hypothetical protein	-1.474	1.15E-12	Down
SS1G_00926	Hypothetical protein	-1.475	6.20E-06	Down
SS1G_13341	Hypothetical protein	-1.478	1.20E-06	Down
SS1G_07889	Mutase	-1.482	7.44E-04	Down
SS1G_10759	Hypothetical protein	-1.483	5.76E-03	Down
SS1G_06203	Hypothetical protein	-1.485	2.16E-03	Down
SS1G_02724	Serine protease	-1.486	8.63E-05	Down
SS1G_12412	Carbohydrate transporter	-1.488	5.94E-03	Down
SS1G_03117	Pre-rRNA processing protein	-1.489	6.91E-04	Down
SS1G_05411	Exoribonuclease	-1.490	3.18E-04	Down
SS1G_04443	Hypothetical protein	-1.490	5.35E-04	Down
SS1G_11873	Damaged DNA-binding protein	-1.491	3.36E-04	Down
SS1G_10774	Hypothetical protein	-1.494	2.98E-04	Down
SS1G_13084	Oxygenase	-1.496	9.85E-06	Down
SS1G_07718	Hypothetical protein	-1.496	1.14E-04	Down
SS1G_09474	Ca2+/H+ antiporter	-1.499	2.04E-04	Down
SS1G_12233	Carbohydrate kinase	-1.501	2.21E-05	Down

SS1G_03122	Nucleic acid binding	-1.501	1.27E-03	Down
SS1G_05163	DNA photolyase	-1.502	2.68E-04	Down
SS1G_10051	Hypothetical protein	-1.504	4.17E-04	Down
SS1G_12551	Ribonucleoprotein	-1.504	1.50E-03	Down
SS1G_00995	Ribosomal protein	-1.504	6.16E-04	Down
SS1G_14484	Hypothetical protein	-1.505	3.71E-05	Down
SS1G_10332	Hypothetical protein	-1.510	2.64E-03	Down
SS1G_03464	Hypothetical protein	-1.513	1.38E-06	Down
SS1G_06628	Hypothetical protein	-1.513	7.19E-06	Down
SS1G_01348	Orotate phosphoribosyltransferase	-1.514	6.91E-06	Down
SS1G_01764	Enzyme modulator	-1.515	2.61E-03	Down
SS1G_04471	Hypothetical protein	-1.517	1.50E-04	Down
SS1G_02270	Hypothetical protein	-1.518	2.13E-03	Down
SS1G_01711	Resistance to glucose repression protein 1	-1.518	3.31E-07	Down
SS1G_02099	Ribosome biogenesis protein	-1.519	2.22E-05	Down
SS1G_06975	Hypothetical protein	-1.524	5.34E-07	Down
SS1G_03480	Hypothetical protein	-1.524	6.58E-04	Down
SS1G_09557	Lipase	-1.526	1.36E-03	Down
SS1G_00185	Deoxynucleotidyltransferase terminal-interac	-1.526	3.63E-04	Down
SS1G_07841	MYB protein	-1.527	1.38E-06	Down
SS1G_04595	Hypothetical protein	-1.527	2.12E-07	Down
SS1G_04626	Hypothetical protein	-1.527	4.56E-04	Down
SS1G_06311	RNA binding protein	-1.530	5.66E-06	Down
SS1G_14122	Glucosamine 6-phosphate N-acetyltransferas	-1.530	3.71E-05	Down
SS1G_02664	Hypothetical protein	-1.530	1.09E-04	Down
SS1G_02854	Hypothetical protein	-1.533	9.27E-05	Down
SS1G_04019	Mitochondrial carrier protein RIM2	-1.536	3.67E-04	Down
SS1G_03491	Hypothetical protein	-1.540	1.03E-04	Down
SS1G_06287	Dehydrogenase	-1.546	8.47E-04	Down
SS1G_12768	Hypothetical protein	-1.547	5.94E-05	Down
SS1G_03293	Hypothetical protein	-1.551	1.06E-03	Down
SS1G_01889	Transferase	-1.552	3.20E-08	Down
SS1G_01089	Hypothetical protein	-1.552	1.43E-04	Down
SS1G_13413	Hypothetical protein	-1.556	1.01E-03	Down
SS1G_10526	ATP-dependent RNA helicase DBP3	-1.559	1.05E-04	Down
SS1G_04472	Hypothetical protein	-1.560	7.73E-04	Down
SS1G_07712	Periodic tryptophan protein 2 homolog	-1.561	1.73E-04	Down
SS1G_10321	Hypothetical protein	-1.565	1.26E-04	Down
SS1G_05890	Hypothetical protein	-1.567	2.48E-06	Down
SS1G_05915	Glucosidase	-1.571	2.18E-04	Down
SS1G_11802	Hypothetical protein	-1.572	1.50E-07	Down
SS1G_14039	ATP-dependent RNA helicase dbp7	-1.573	1.47E-03	Down
SS1G_11752	Hypothetical protein	-1.573	1.24E-07	Down
SS1G_03305	Hypothetical protein	-1.574	3.47E-05	Down
SS1G_09744	Hypothetical protein	-1.577	1.60E-06	Down
SS1G_04721	Hypothetical protein	-1.577	2.83E-04	Down
SS1G_13013	Hypothetical protein	-1.578	2.77E-04	Down

SS1G_11245	Hypothetical protein	-1.584	1.42E-03	Down
SS1G_08323	ATP-dependent RNA helicase dbp4	-1.585	7.30E-04	Down
SS1G_04161	Methyltransferase	-1.585	8.43E-06	Down
SS1G_09791	Hypothetical protein	-1.586	4.87E-07	Down
SS1G_03440	Dehydrogenase	-1.586	5.80E-03	Down
SS1G_08822	Large subunit GTPase 1 homolog	-1.590	5.32E-06	Down
SS1G_13165	Hypothetical protein	-1.591	4.02E-03	Down
SS1G_14123	Hypothetical protein	-1.592	7.22E-06	Down
SS1G_13734	Carbohydrate transporter	-1.594	2.58E-04	Down
SS1G_12340	ATP-dependent RNA helicase ROK1	-1.594	2.91E-04	Down
SS1G_03118	Pre-rRNA processing protein	-1.594	5.35E-04	Down
SS1G_06986	tRNA-splicing endonuclease subunit SEN15	-1.597	1.33E-04	Down
SS1G_11046	Nucleic acid binding	-1.601	3.42E-05	Down
SS1G_07544	RNA helicase	-1.602	2.73E-03	Down
SS1G_06802	Hypothetical protein	-1.603	1.63E-04	Down
SS1G_04627	Hypothetical protein	-1.604	2.16E-05	Down
SS1G_13414	RNA methyltransferase	-1.605	1.16E-03	Down
SS1G_02632	Hypothetical protein	-1.608	1.81E-05	Down
SS1G_015443	Hypothetical protein	-1.610	3.78E-03	Down
SS1G_08479	tRNA-splicing endonuclease subunit Sen34	-1.616	1.17E-04	Down
SS1G_09201	Hypothetical protein	-1.618	8.90E-05	Down
SS1G_04084	Hypothetical protein	-1.618	2.06E-06	Down
SS1G_07742	Ribosome production factor 2 homolog	-1.620	1.29E-04	Down
SS1G_03773	Hypothetical protein	-1.620	3.07E-07	Down
SS1G_09064	Nuclear transport factor 2 family protein	-1.620	1.03E-03	Down
SS1G_03815	U3 small nucleolar ribonucleoprotein protein	-1.623	4.30E-04	Down
SS1G_00436	60s ribosomal subunit assembly/export prote	-1.624	5.20E-05	Down
SS1G_12070	ATP-binding cassette (ABC) transporter	-1.625	2.87E-04	Down
SS1G_10389	Hypothetical protein	-1.630	1.46E-06	Down
SS1G_09792	K+-H+ exchange-like protein	-1.633	3.87E-04	Down
SS1G_14217	Neuroguidin	-1.634	2.89E-04	Down
SS1G_00825	Hypothetical protein	-1.636	7.22E-04	Down
SS1G_10390	Hypothetical protein	-1.637	4.59E-06	Down
SS1G_01657	Linoleate diol synthase	-1.637	7.88E-06	Down
SS1G_02283	Hypothetical protein	-1.640	2.07E-03	Down
SS1G_01524	Endodeoxyribonuclease	-1.640	2.21E-04	Down
SS1G_03037	Hypothetical protein	-1.641	2.88E-05	Down
SS1G_06778	Hypothetical protein	-1.644	1.92E-04	Down
SS1G_09558	Pre-rRNA-processing protein TSR2 homolog	-1.644	8.09E-06	Down
SS1G_06425	ATP-dependent RNA helicase dbp9	-1.645	1.34E-04	Down
SS1G_01857	Hypothetical protein	-1.646	7.35E-04	Down
SS1G_04291	Mitochondrial distribution and morphology p	-1.647	1.14E-04	Down
SS1G_07235	Hypothetical protein	-1.654	3.15E-04	Down
SS1G_06193	Hypothetical protein	-1.657	1.10E-03	Down
SS1G_10992	Metacaspase-1	-1.658	5.09E-05	Down
SS1G_11274	Hypothetical protein	-1.658	5.65E-04	Down
SS1G_12276	Hypothetical protein	-1.659	8.87E-06	Down

SS1G_02973	Hypothetical protein	-1.659	8.10E-05	Down
SS1G_03294	Small RAS GTPase	-1.661	7.63E-04	Down
SS1G_08322	ATP-dependent RNA helicase dbp10	-1.662	2.41E-03	Down
SS1G_05229	Hypothetical protein	-1.667	1.07E-05	Down
SS1G_014508	Hypothetical protein	-1.670	4.70E-07	Down
SS1G_03766	RRO15-like protein	-1.672	7.82E-05	Down
SS1G_10199	Nuclear valosin-containing protein	-1.672	3.48E-04	Down
SS1G_09166	Hypothetical protein	-1.673	1.16E-04	Down
SS1G_01283	Hypothetical protein	-1.674	1.10E-06	Down
SS1G_07027	Hypothetical protein	-1.675	1.41E-07	Down
SS1G_03435	Transporter	-1.676	5.81E-04	Down
SS1G_016130	Hypothetical protein	-1.678	1.12E-04	Down
SS1G_06566	Hypothetical protein	-1.681	4.24E-06	Down
SS1G_05631	U3 small nucleolar RNA-interacting protein 2	-1.682	4.56E-04	Down
SS1G_08570	Protein bcp1	-1.682	7.44E-05	Down
SS1G_09465	Hypothetical protein	-1.682	4.20E-03	Down
SS1G_13972	Hypothetical protein	-1.682	1.24E-03	Down
SS1G_14483	Hypothetical protein	-1.682	9.50E-05	Down
SS1G_09029	Hypothetical protein	-1.683	2.76E-04	Down
SS1G_07522	Transporter	-1.684	7.51E-04	Down
SS1G_09121	Aldo-keto reductase	-1.686	1.61E-05	Down
SS1G_02359	Transmembrane protein 53	-1.688	5.82E-03	Down
SS1G_09190	Carbohydrate kinase	-1.688	2.37E-06	Down
SS1G_07355	Ph-response transcription factor pacc/rim101	-1.689	1.01E-04	Down
SS1G_10075	ATP-dependent RNA helicase	-1.691	4.81E-03	Down
SS1G_04983	U3 small nucleolar RNA-associated protein 1	-1.692	1.66E-04	Down
SS1G_01347	Nucleolar GTP-binding protein 1	-1.695	2.32E-05	Down
SS1G_02886	Glycine-rich RNA-binding protein	-1.695	6.37E-13	Down
SS1G_11478	Ankyrin-2	-1.699	2.33E-04	Down
SS1G_04940	Hypothetical protein	-1.700	5.81E-04	Down
SS1G_08623	DNA-directed RNA polymerase	-1.701	4.20E-05	Down
SS1G_05110	Mannan endo-1,6-alpha-mannosidase	-1.706	9.94E-07	Down
SS1G_07511	Hypothetical protein	-1.706	4.13E-07	Down
SS1G_08110	Hypothetical protein	-1.706	2.03E-04	Down
SS1G_09841	Hypothetical protein	-1.707	5.53E-03	Down
SS1G_13028	Hypothetical protein	-1.707	2.27E-03	Down
SS1G_09602	Protein DOM34 homolog	-1.707	1.16E-05	Down
SS1G_03774	Hypothetical protein	-1.711	1.87E-04	Down
SS1G_14344	RRP12-like protein	-1.711	1.21E-04	Down
SS1G_13166	2-dehydropantoate 2-reductase-related prote	-1.712	2.13E-05	Down
SS1G_04290	RNA-binding protein PNO1	-1.713	1.99E-05	Down
SS1G_01671	Hypothetical protein	-1.715	4.56E-04	Down
SS1G_00416	Hypothetical protein	-1.719	8.35E-03	Down
SS1G_05913	Hypothetical protein	-1.724	3.18E-04	Down
SS1G_02959	DNA-directed RNA polymerase	-1.724	4.58E-06	Down
SS1G_00363	Polyadenylate-binding protein	-1.726	6.21E-07	Down
SS1G_13459	Flotillin-like protein 1	-1.728	6.68E-05	Down

SS1G_05099	WD repeat-containing protein 46	-1.730	1.81E-05	Down
SS1G_01734	Hypothetical protein	-1.732	4.81E-03	Down
SS1G_10361	Hypothetical protein	-1.732	4.74E-03	Down
SS1G_00057	Lipase	-1.741	4.63E-06	Down
SS1G_01881	MYB-binding protein 1A	-1.741	2.32E-04	Down
SS1G_14323	Hypothetical protein	-1.748	5.21E-03	Down
SS1G_07025	Hypothetical protein	-1.748	6.20E-05	Down
SS1G_01816	Cysteine protease	-1.753	1.49E-09	Down
SS1G_01658	Hypothetical protein	-1.755	3.60E-07	Down
SS1G_09143	Hypothetical protein	-1.756	6.54E-08	Down
SS1G_07406	ProteinKRI1 homolog	-1.758	5.21E-04	Down
SS1G_05474	Hypothetical protein	-1.763	1.32E-05	Down
SS1G_06095	Hypothetical protein	-1.763	4.94E-06	Down
SS1G_03082	Ribosome biogenesis protein ytm1	-1.766	7.68E-05	Down
SS1G_06567	ATP-dependent rRNA helicase rrp3	-1.769	3.68E-05	Down
SS1G_07247	Hypothetical protein	-1.769	5.84E-04	Down
SS1G_13001	Transcriptional regulatory protein moc3	-1.772	5.20E-05	Down
SS1G_08632	Hypothetical protein	-1.773	2.16E-05	Down
SS1G_13820	Hypothetical protein	-1.774	6.93E-03	Down
SS1G_10999	U3 small nucleolar RNA-associated protein 22	-1.776	1.39E-03	Down
SS1G_02048	Transporter	-1.777	8.90E-05	Down
SS1G_01720	Sulfite efflux pump	-1.779	8.78E-03	Down
SS1G_09680	Nucleolar complex protein 3 homolog	-1.780	1.37E-04	Down
SS1G_05100	Pre-rRNA-processing protein TSR1	-1.781	1.25E-06	Down
SS1G_07969	Hypothetical protein	-1.785	3.99E-07	Down
SS1G_02988	Hypothetical protein	-1.787	1.86E-03	Down
SS1G_02902	Transcription factor	-1.788	1.60E-05	Down
SS1G_13224	Hypothetical protein	-1.795	3.70E-09	Down
SS1G_09035	Nucleolar protein 4	-1.797	2.27E-04	Down
SS1G_11303	Hypothetical protein	-1.799	2.59E-08	Down
SS1G_02533	Nucleolar GTP-binding protein 2	-1.805	2.18E-04	Down
SS1G_03321	Hypothetical protein	-1.805	2.88E-05	Down
SS1G_08823	Protein ITV1 homolog	-1.806	6.89E-06	Down
SS1G_12214	Nucleolar protein 58	-1.807	1.27E-05	Down
SS1G_02092	Hypothetical protein	-1.807	8.63E-05	Down
SS1G_02284	Transcription factor	-1.811	3.01E-04	Down
SS1G_00361	Hypothetical protein	-1.814	1.01E-04	Down
SS1G_01564	Guanine nucleotide-binding protein	-1.814	5.73E-05	Down
SS1G_01210	Phosphatase	-1.817	5.39E-06	Down
SS1G_00362	Hypothetical protein	-1.818	5.87E-05	Down
SS1G_07874	Hypothetical protein	-1.818	4.59E-07	Down
SS1G_12560	Hypothetical protein	-1.819	1.44E-06	Down
SS1G_12778	Hypothetical protein	-1.820	9.91E-03	Down
SS1G_02382	ABC transporter G family member	-1.820	4.42E-05	Down
SS1G_12417	Hypothetical protein	-1.827	3.75E-05	Down
SS1G_00845	Hypothetical protein	-1.834	1.81E-05	Down
SS1G_02235	Nucleolar protein 10	-1.834	8.09E-05	Down

SS1G_13732	Endochitinase	-1.838	7.50E-05	Down
SS1G_02848	Hypothetical protein	-1.841	1.01E-08	Down
SS1G_00678	Something about silencing protein 10	-1.845	5.08E-05	Down
SS1G_12994	U3 small nucleolar RNA-associated protein 4	-1.849	5.32E-05	Down
SS1G_02383	ABC transporter G family member	-1.851	1.48E-05	Down
SS1G_13924	mRNA splicing factor	-1.855	3.75E-05	Down
SS1G_08782	Protein mak16	-1.860	1.25E-04	Down
SS1G_12786	Hypothetical protein	-1.861	8.94E-03	Down
SS1G_00317	Nuclease	-1.862	1.56E-04	Down
SS1G_07812	Fukutin	-1.863	9.74E-04	Down
SS1G_04436	RNA binding protein	-1.863	6.06E-05	Down
SS1G_04164	Protein pxr1	-1.868	1.71E-05	Down
SS1G_08552	Hypothetical protein	-1.870	1.01E-05	Down
SS1G_02358	Transporter	-1.884	3.08E-03	Down
SS1G_07448	Hypothetical protein	-1.891	1.48E-05	Down
SS1G_04473	Hypothetical protein	-1.892	5.23E-06	Down
SS1G_08391	Tumor suppressor candidate protein	-1.893	1.76E-05	Down
SS1G_06909	Hypothetical protein	-1.894	1.18E-07	Down
SS1G_04356	Pescadillo homolog	-1.895	5.37E-06	Down
SS1G_03691	Hypothetical protein	-1.896	6.28E-03	Down
SS1G_10150	RNA helicase	-1.897	8.41E-06	Down
SS1G_02987	Hypothetical protein	-1.905	2.81E-05	Down
SS1G_12540	ATP-dependent RNA helicase drs1	-1.907	1.17E-05	Down
SS1G_05304	Aromatic peroxygenase	-1.909	1.46E-13	Down
SS1G_12476	Hypothetical protein	-1.910	2.05E-06	Down
SS1G_11991	Oxidoreductase	-1.912	6.06E-05	Down
SS1G_01694	Hypothetical protein	-1.912	3.96E-06	Down
SS1G_05865	tRNA-dihydrouridine(47) synthase [NAD(P)(+)	-1.913	1.61E-05	Down
SS1G_00071	ATP-dependent RNA helicase dbp2	-1.918	2.05E-06	Down
SS1G_01745	Decarboxylase	-1.930	5.42E-14	Down
SS1G_00351	Hypothetical protein	-1.931	2.33E-05	Down
SS1G_00639	Ribonucleoprotein	-1.934	3.15E-05	Down
SS1G_04857	Endopolygalacturonase	-1.934	4.44E-06	Down
SS1G_02682	Mannan endo-1,6-alpha-mannosidase	-1.946	1.33E-04	Down
SS1G_01835	Translation initiation factor	-1.946	2.91E-08	Down
SS1G_04762	Hypothetical protein	-1.949	2.43E-04	Down
SS1G_05854	WD repeat-containing protein 75	-1.950	1.58E-05	Down
SS1G_07286	Hypothetical protein	-1.951	2.98E-04	Down
SS1G_04139	Nucleolar protein 14	-1.951	9.71E-05	Down
SS1G_12287	Hypothetical protein	-1.952	1.51E-09	Down
SS1G_06457	Vacuolar protein sorting-associated protein	-1.955	6.90E-07	Down
SS1G_14329	WD repeat-containing protein 43	-1.956	2.71E-05	Down
SS1G_14385	Transcription factor	-1.962	7.62E-05	Down
SS1G_09887	Hypothetical protein	-1.964	6.66E-03	Down
SS1G_03009	DNA-directed RNA polymerase	-1.964	9.80E-25	Down
SS1G_05105	Hypothetical protein	-1.973	1.24E-03	Down
SS1G_08624	Hypothetical protein	-1.978	1.36E-03	Down

SS1G_08193	Phospholipase D	-1.982	8.63E-05	Down
SS1G_13549	Hypothetical protein	-1.987	5.87E-05	Down
SS1G_02351	Importin-4	-1.987	2.69E-07	Down
SS1G_11781	Amino acid transporter	-1.993	1.45E-07	Down
SS1G_14068	ESF1 homolog	-1.996	6.54E-06	Down
SS1G_13144	Hypothetical protein	-1.997	6.73E-05	Down
SS1G_11737	Hypothetical protein	-1.997	8.54E-17	Down
SS1G_03893	Hypothetical protein	-1.998	7.43E-06	Down
SS1G_01822	rRNA-processing protein	-2.011	1.04E-07	Down
SS1G_01682	tRNA adenine methyltransferase catalytic sul	-2.012	2.53E-05	Down
SS1G_11246	Methyltransferase	-2.016	1.20E-06	Down
SS1G_08142	Hypothetical protein	-2.016	6.74E-06	Down
SS1G_10391	Hypothetical protein	-2.017	8.98E-05	Down
SS1G_06776	Pumilio homolog 3	-2.022	2.90E-05	Down
SS1G_11736	Nucleolar protein 9	-2.023	5.01E-14	Down
SS1G_05969	Hypothetical protein	-2.026	9.29E-04	Down
SS1G_08188	Dehydrogenase	-2.031	1.46E-04	Down
SS1G_06620	Carbohydrate transporter	-2.037	1.34E-03	Down
SS1G_07557	Ribosomal protein	-2.041	1.44E-06	Down
SS1G_11815	Hypothetical protein	-2.046	1.83E-06	Down
SS1G_03439	Hypothetical protein	-2.047	9.93E-04	Down
SS1G_06728	Transporter	-2.052	4.55E-03	Down
SS1G_00252	DNA-directed RNA polymerase I subunit	-2.053	5.14E-07	Down
SS1G_03775	Hypothetical protein	-2.056	5.04E-07	Down
SS1G_09525	Hypothetical protein	-2.057	4.92E-05	Down
SS1G_06841	Amino acid transporter	-2.062	7.81E-08	Down
SS1G_10506	Hypothetical protein	-2.064	4.49E-06	Down
SS1G_09799	Hypothetical protein	-2.085	2.56E-04	Down
SS1G_01834	Ubiquitin carboxyl-terminal hydrolase	-2.088	1.53E-09	Down
SS1G_11751	Hypothetical protein	-2.090	1.18E-04	Down
SS1G_01733	Hypothetical protein	-2.098	5.04E-07	Down
SS1G_13470	Hypothetical protein	-2.098	1.02E-06	Down
SS1G_14069	ESF1 homolog	-2.107	7.75E-06	Down
SS1G_09888	U3 small nucleolar RNA-associated protein 18	-2.116	4.14E-06	Down
SS1G_09508	Hypothetical protein	-2.119	1.92E-03	Down
SS1G_07285	Hypothetical protein	-2.121	9.58E-06	Down
SS1G_02495	Peroxidase	-2.124	5.92E-08	Down
SS1G_11064	Hypothetical protein	-2.126	3.44E-04	Down
SS1G_01537	Hypothetical protein	-2.128	9.42E-05	Down
SS1G_05921	Hypothetical protein	-2.129	1.37E-07	Down
SS1G_03045	Hypothetical protein	-2.129	5.34E-04	Down
SS1G_11673	Hypothetical protein	-2.133	7.96E-04	Down
SS1G_08280	Hypothetical protein	-2.145	3.02E-03	Down
SS1G_04205	Protein HGH1 homolog	-2.146	6.90E-07	Down
SS1G_12776	Hypothetical protein	-2.151	1.18E-03	Down
SS1G_05985	P21-activated protein kinase-interacting prot	-2.158	7.12E-06	Down
SS1G_05094	Dehydrogenase	-2.164	5.83E-05	Down

SS1G_08220	Hypothetical protein	-2.177	4.64E-06	Down
SS1G_01759	Sugar transporter	-2.184	7.82E-05	Down
SS1G_03941	Aspartic protease	-2.187	7.15E-11	Down
SS1G_07446	Hypothetical protein	-2.194	5.28E-08	Down
SS1G_12561	Hypothetical protein	-2.202	8.50E-09	Down
SS1G_10998	20s rRNA accumulation protein 4	-2.210	1.74E-06	Down
SS1G_07876	Hypothetical protein	-2.213	4.65E-05	Down
SS1G_12791	Hypothetical protein	-2.222	2.28E-05	Down
SS1G_08187	Hypothetical protein	-2.234	4.75E-05	Down
SS1G_08952	Hypothetical protein	-2.235	1.22E-05	Down
SS1G_01264	Chromaffin granule amine transporter	-2.242	2.48E-09	Down
SS1G_03942	Hypothetical protein	-2.244	2.21E-10	Down
SS1G_03455	Non-receptor serine/threonine protein kinase	-2.245	3.49E-06	Down
SS1G_04923	Hypothetical protein	-2.267	1.52E-17	Down
SS1G_07873	RNA-binding protein	-2.272	1.31E-06	Down
SS1G_05067	Hypothetical protein	-2.293	5.08E-05	Down
SS1G_12790	Methyltransferase	-2.296	4.44E-06	Down
SS1G_01676	*C. elegans* chromodomain protein	-2.299	7.83E-08	Down
SS1G_13477	Transmembrane protein	-2.303	5.03E-10	Down
SS1G_02117	Carbohydrate transporter	-2.313	5.46E-05	Down
SS1G_02541	Hypothetical protein	-2.326	4.98E-08	Down
SS1G_10155	Polyamine transporter 1	-2.359	1.30E-06	Down
SS1G_06527	Hypothetical protein	-2.366	1.25E-03	Down
SS1G_11780	Hypothetical protein	-2.368	1.61E-09	Down
SS1G_00864	Amino acid transporter	-2.379	1.18E-07	Down
SS1G_00872	Hypothetical protein	-2.387	9.73E-14	Down
SS1G_05211	Hypothetical protein	-2.399	5.66E-06	Down
SS1G_11030	Gemini, isoform C	-2.403	1.60E-10	Down
SS1G_07021	Hypothetical protein	-2.403	9.39E-06	Down
SS1G_07447	Hypothetical protein	-2.419	4.95E-10	Down
SS1G_12506	Hypothetical protein	-2.422	2.62E-04	Down
SS1G_07445	Hypothetical protein	-2.441	4.87E-07	Down
SS1G_07412	3',5'-cyclic-nucleotide phosphodiesterase	-2.442	1.92E-06	Down
SS1G_05227	Hypothetical protein	-2.444	2.22E-05	Down
SS1G_07022	Hypothetical protein	-2.475	2.21E-06	Down
SS1G_03709	Hypothetical protein	-2.482	1.26E-04	Down
SS1G_00769	Hypothetical protein	-2.532	3.98E-05	Down
SS1G_05237	Phosphatase	-2.545	1.27E-05	Down
SS1G_07463	Lipase	-2.593	1.84E-06	Down
SS1G_07462	Hypothetical protein	-2.610	5.99E-08	Down
SS1G_10125	Carbohydrate transporter	-2.610	2.98E-10	Down
SS1G_01755	Hypothetical protein	-2.614	4.80E-06	Down
SS1G_08282	Glucosidase	-2.627	5.37E-06	Down
SS1G_00768	Hypothetical protein	-2.679	1.89E-07	Down
SS1G_01576	Oxidase	-2.694	6.42E-10	Down
SS1G_07667	Clock-controlled protein 6	-2.721	1.26E-15	Down
SS1G_12513	LysM domain-containing protein	-2.739	1.02E-10	Down

SS1G_01032	Hypothetical protein	-2.820	5.68E-05	Down
SS1G_04683	Hypothetical protein	-2.835	1.01E-10	Down
SS1G_07359	Hypothetical protein	-2.948	5.86E-10	Down
SS1G_11789	Acyltransferase	-3.092	1.62E-07	Down
SS1G_01754	Hypothetical protein	-3.161	9.88E-13	Down
SS1G_01651	Transporter	-3.177	6.43E-11	Down
SS1G_05917	Hypothetical protein	-3.190	3.12E-18	Down
SS1G_01382	Serine protease	-3.252	5.64E-09	Down
SS1G_016850	Hypothetical protein	-3.304	7.54E-15	Down
SS1G_02499	Hypothetical protein	-3.447	3.47E-21	Down
SS1G_04053	Lyase	-3.600	1.43E-13	Down
SS1G_07295	Hypothetical protein	-3.640	1.06E-10	Down
SS1G_08838	Hypothetical protein	-3.788	1.75E-19	Down
SS1G_06453	Hypothetical protein	-3.824	1.59E-13	Down
SS1G_03747	Hypothetical protein	-3.837	9.92E-15	Down
SS1G_06412	Hypothetical protein	-3.866	5.94E-12	Down
SS1G_13051	Cellobiose dehydrogenase	-3.916	2.66E-11	Down

Table S3. Numbers of aligning small RNA sequence reads from virus-free and hypovirus-transfected *Sclerotinia sclerotiorum*

	Library								Total	
	Virus-free_1		Virus-free_2		Virus-infected_1		Virus-infected_2			
	Count	%	Count	%	Count	%	Count	%	Count	%
Filtered reads	23,834,355		19,972,296		24,093,727		22,463,731		90,364,109	
S. sclerotiorum genome	15,174,888	63.67%	15,322,059	76.72%	11,797,995	48.97%	14,483,400	64.47%	56,778,344	63.46%
Predicted coding sequences	5,038,538	21.14%	4,939,512	24.73%	6,292,899	26.12%	4,973,533	22.14%	21,244,483	23.53%
Ribosomal RNA	2,735,533	11.50%	4,182,191	20.90%	3,025,071	12.60%	1,480,630	6.60%	11,423,425	12.90%
Predicted tRNAs	5,475,657	22.97%	4,746,585	23.77%	3,507,677	14.56%	9,799,798	43.62%	23,529,717	26.04%
Retrotransposons	1,385,373	5.80%	1,833,908	9.20%	961,857	4.00%	1,060,959	4.70%	5,242,097	5.93%
Mitochondrial genome	3,343,171	14.03%	1,662,714	8.33%	3,835,055	15.92%	2,771,460	12.34%	11,612,400	12.85%
Sclerotinia sclerotiorum hypovirus 2L	NA	NA	NA	NA	2,677,788	11.11%	1,555,299	6.92%	4,233,087	9.02%

Table S4. Candidate microRNA sequence predicted from for *Sclerotinia sclerotiorum* small RNA sequence data by five miRNA prediction programs

Name	Sequence	mirdeep 2	MiRDeep *	miRDP	mireap	MiRPlant	Times predicted	Total Reads
candidate_miRNA_0001	AAUCCUUCUCUCUGCUCUUCGGC	X	X	X		X	4	156
candidate_miRNA_0002	CAAGGUCGCUGUGCAGUCAAACC	X	X	X	X		4	40
candidate_miRNA_0006	AUCUAUCUUCUCUUCUCAAUUG		X	X		X	3	29
candidate_miRNA_0007	GGCGUCGUGACGGAGUGGUUAAC		X	X		X	3	121,853
candidate_miRNA_0008	UAAAGGCUGCAGAUAGGUGAGG	X		X		X	3	24
candidate_miRNA_0009	UAAGGUUUCGUCUGGUUGGUAA		X	X		X	3	109
candidate_miRNA_0010	UAAUGGUAUAUGACUUCUGCCA		X	X		X	3	46
candidate_miRNA_0011	UACCUAGUAGACAUUCGUGUAAC	X		X		X	3	31
candidate_miRNA_0012	UAUGACGCUAAUGUGGGACAUA	X	X	X			3	24
candidate_miRNA_0013	UCAAGACUCUGCACAUGGCACA	X		X		X	3	30
candidate_miRNA_0015	UGAUGGAUAGACUGGAUAGCC		X	X		X	3	124
candidate_miRNA_0017	UGGCGAUUGGAUUGGAGGCAGG	X	X			X	3	21
candidate_miRNA_0018	UGUCGAGAUGCUGGAGCUGAAGC	X	X			X	3	26
candidate_miRNA_0019	UUCACGAGUGCAUUUACAGACAG	X	X	X			3	44
candidate_miRNA_0020	UUCAUUCAUUCAUAGGAUUGCA		X	X		X	3	145
candidate_miRNA_0021	UUCCAAGUGGAGACUGAGCUAC		X	X		X	3	34
candidate_miRNA_0022	UUCCGAAAGUCGAUCGACUCUU	X		X	X		3	1,095
candidate_miRNA_0023	UUGUCGCUGGUCGUCAUGGCGC	X	X	X			3	61
candidate_miRNA_0024	UUUCCCUGAGGUAUCCUGGCUA	X	X			X	3	6,962
candidate_miRNA_0027	AGAAUAGUUUCAUAGUUUAGGAG		X			X	2	290
candidate_miRNA_0028	AGAUUCUGACUGUUAUGACCAG			X		X	2	26
candidate_miRNA_0029	AGUCAUUCUGAAUUCCGAUCUG			X		X	2	34
candidate_miRNA_0031	AUCCGCACUCUUGAACCGAGUGG		X			X	2	276
candidate_miRNA_0032	CAAAGCUAUGGACCCGAGUACA	X				X	2	126
candidate_miRNA_0033	CACACUGUUUGGACGGAGAUAG		X	X			2	21
candidate_miRNA_0034	CAGGAACCAUUCACUUUAACAG		X			X	2	273
candidate_miRNA_0035	CAGUAUUCUUGAGUGUGACAG		X			X	2	206
candidate_miRNA_0037	CGGAGGUGUGAGGCGUCACAG		X			X	2	157
candidate_miRNA_0038	GUAAGUUAUGCGGACUGUGCUU	X		X			2	161
candidate_miRNA_0040	UAAUGAGAAAUAUCGUCUGUAGA		X	X			2	31
candidate_miRNA_0047	UACGGACUGAAUACGGAGUGGCC		X			X	2	29
candidate_miRNA_0050	UAGACCUUCGUGUUCUGGCUC		X			X	2	97
candidate_miRNA_0051	UAGACGGCAAACCUCGUAACAAA			X		X	2	30
candidate_miRNA_0052	UAGAGGUUAGAUGUCAUGUACU	X		X			2	115
candidate_miRNA_0053	UAGCGCGGCAGUGGCAACAUGC	X		X			2	58
candidate_miRNA_0055	UAGGAUAAUUGUCCUGGGACAA		X			X	2	53
candidate_miRNA_0056	UAGGAUACCCAUUAGACUUUCAG		X			X	2	32
candidate_miRNA_0058	UAUACGGCUCGAGAGGACGGAUG			X		X	2	124
candidate_miRNA_0061	UAUGAGAUUUUGAUCAGGGUUG		X	X			2	80

Table S4 Continued

candidate_miRNA_0062	UCAAGUAGAAUAUAGGUAAAC		X			X	2	149
candidate_miRNA_0063	UCAAGUAGAAUGUAGAUAAAC		X			X	2	146
candidate_miRNA_0064	UCAAUUACUUAGAAUCUAAAAC		X			X	2	41
candidate_miRNA_0066	UCAUCCAGAUCGUAAGUAAAGC	X				X	2	54
candidate_miRNA_0067	UCAUGUGGAAGUGCUCUAGGC		X			X	2	3,473
candidate_miRNA_0069	UCCACAUGAUCUAGACGGCAAAC		X			X	2	3,955
candidate_miRNA_0070	UCCCUCGUUGGAGCCACAAAAG			X		X	2	71
candidate_miRNA_0073	UCGAGAUGUUGUCAAUCGUUGC			X		X	2	83
candidate_miRNA_0074	UCGAGGUAGUUUAUAACUAGGCG			X		X	2	76,317
candidate_miRNA_0075	UCGAGGUGGUUUAUAACUAGACG		X			X	2	30,174
candidate_miRNA_0077	UCUAGGAGCUUUGAGGGCUC		X	X			2	56
candidate_miRNA_0079	UCUCCUAGGAUAAUUUAACUCC		X	X			2	30
candidate_miRNA_0080	UCUCCUGGGAUAAUAUAACUCC		X	X			2	30
candidate_miRNA_0081	UCUCGAGAUUAGAACUUAAUGC		X	X			2	39
candidate_miRNA_0082	UCUCUUGGAUCUACUGGAUCUA		X	X			2	23
candidate_miRNA_0083	UGAACUGAUCAAUAGCUACAUC	X		X			2	33
candidate_miRNA_0084	UGACACUUUCAAGAAUUGGAUG			X		X	2	2,485
candidate_miRNA_0085	UGACCGUAUCGAAUAUUUUGAGC		X	X			2	20
candidate_miRNA_0086	UGACUAGUGGGACUUUUCACU	X		X			2	197
candidate_miRNA_0087	UGACUUGUUCUUUCUUUGAAUC	X		X			2	1,222
candidate_miRNA_0089	UGAGAGAGCUUUGUAUGUACAUC	X		X			2	101
candidate_miRNA_0091	UGAUGAAUCGAUGGGAAAGGAUA			X		X	2	31
candidate_miRNA_0092	UGAUUAUAUAGACCUUCGUGC		X			X	2	693
candidate_miRNA_0093	UGAUUGAUCUUGAUCUUGAUC			X		X	2	64
candidate_miRNA_0095	UGCCGUCUAGAUCAUGUGGAAG	X				X	2	15,712
candidate_miRNA_0096	UGCGGGUUCGUUUGGAUGUAUG		X	X			2	37
candidate_miRNA_0097	UGCUCCAGUUGUUCUGACUAGG	X	X				2	104
candidate_miRNA_0098	UGGAUUGUGCUUUUGUAAAAUC	X		X			2	25
candidate_miRNA_0099	UGGCAAGGAUUAGUUUCUGUCC	X	X				2	41
candidate_miRNA_0104	UGGGGGAAGGUCGCCUGAAGUCU	X		X			2	126
candidate_miRNA_0105	UGGGUUAGUGCGAGAUGAUGAAC		X	X			2	23
candidate_miRNA_0106	UGUACGCAUGGCUUCUUGAACA		X			X	2	26
candidate_miRNA_0107	UGUAGAGGAGAGGUUGAAUAUG		X	X			2	212
candidate_miRNA_0109	UGUGUACUUGAGGAUUAUGCCC		X			X	2	4,284
candidate_miRNA_0110	UUAAACCGGAUAGAGAAAGCUCU			X	X		2	42
candidate_miRNA_0112	UUAGUAUACGAUUUAGGUGACC		X			X	2	67
candidate_miRNA_0113	UUAGUCUCGUAGUUAAGGAGC		X			X	2	728
candidate_miRNA_0115	UUGAGUAUGAAGAUUGAGCAUG		X	X			2	24
candidate_miRNA_0116	UUGAUUAUAUAGACCUUUGUGCC		X	X			2	282
candidate_miRNA_0122	UUGUGGUUUAGUGGUAUAAUGU		X			X	2	30
candidate_miRNA_0123	UUUGAGAGCUGAUAAGUUGGAUA	X	X				2	46
candidate_miRNA_0129	UAGCCUAGGAAUUCUGUUUCUG			X			1	32
candidate_miRNA_0132	AAGACUAGGUUUGAAGGGAUAU			X			1	43

Table S4 Continued

candidate_miRNA_0136	AAGUCUUCCACCUCGCUCGACC		X				1	32
candidate_miRNA_0137	AAUCCGGAUUCGAUAGAAAUGG					X	1	28
candidate_miRNA_0139	ACAAGUAGAAUGUAGGUAAACU			X			1	32
candidate_miRNA_0141	ACAUGGAAGUGCUCUAGGCU			X			1	843
candidate_miRNA_0142	ACAUGGAAGUGCUCUAGGCUCU				X		1	124
candidate_miRNA_0143	ACCACAAAAUAGCACGAUCGAUG			X			1	42
candidate_miRNA_0150	AGACCGAGGGGAUCGAAGGGAC		X				1	121
candidate_miRNA_0152	AGAGGAUUCGGGAAUGAGAAUU	X					1	53
candidate_miRNA_0154	AGAGGGAUCUUGGAAGAACACU			X			1	25
candidate_miRNA_0157	AGGACAUGCCAGAAUGCUUUGG					X	1	23
candidate_miRNA_0160	AGUUCGAUCGUCUAUGGAGCCAUC				X		1	31
candidate_miRNA_0162	AGUUGAUUGUGCAUCUUGAGC			X			1	43
candidate_miRNA_0163	AGUUGGUUAGAGCGUUGGUCU				X		1	21
candidate_miRNA_0197	UAUAUAGUCCUUCAUGCUUCCA			X			1	52
candidate_miRNA_0206	AUGGCGUGCGAGAUUAAGGAUG			X			1	37
candidate_miRNA_0212	AUGUCUCAAUGAACUGAUUGCCU			X			1	185
candidate_miRNA_0227	AUUGUUAUUACUUAAGUCCCUG					X	1	44
candidate_miRNA_0241	CACCUGGAUCGAGUCGCUUUACG		X				1	66
candidate_miRNA_0246	CAGACGAAACCUCAGGCAGCC					X	1	561
candidate_miRNA_0247	CAGACGAAACCUCAGGCAGCCG					X	1	2,990
candidate_miRNA_0253	CAUAACCGGGUCCAUGUACUG		X				1	21
candidate_miRNA_0254	CAUCAAAUAUUUGGAACAAACC					X	1	5,732
candidate_miRNA_0256	CAUCGGAAGUUUCGGCUGCAGA			X			1	31
candidate_miRNA_0258	CCAUUAUUGUCAUUACUUAAG					X	1	30
candidate_miRNA_0259	CCCUUUGUAUAGAGCUUCGUA			X			1	26
candidate_miRNA_0261	CCGAGACGAUGAGCCGAACC		X				1	2,865
candidate_miRNA_0264	CGAAGGGAUCGAAGGGAUCA		X				1	23
candidate_miRNA_0265	CGAGAAUUGCUGAAGAACUAUG					X	1	29
candidate_miRNA_0267	CGAGGCUGACAUGAUGGAUACC		X				1	79
candidate_miRNA_0271	CGGAGGUGUGAGGCGUCACAC					X	1	180
candidate_miRNA_0272	CGGAGGUGUGAGGCGUCACACA					X	1	2,542
candidate_miRNA_0273	CGGAGGUGUGAGGCGUCACAGG		X				1	1,139
candidate_miRNA_0275	CGGGAUGAAUCAAUGAAUACAUG			X			1	162
candidate_miRNA_0276	CGGGAUUAGAACAAGGGACAUUU			X			1	66
candidate_miRNA_0285	CUAUAUCAUCUUUUGAGGAUC		X				1	47
candidate_miRNA_0302	CUGCCUAGUCGGCUGUAUUUCA					X	1	24
candidate_miRNA_0303	CUGGAGGCAGCGGCUACCUAAGG			X			1	24
candidate_miRNA_0318	CUUUAUGAAUGAUGAUUAUUACU			X			1	30
candidate_miRNA_0324	GAAAUCGAUAAAUCCUAUGGCA			X			1	24
candidate_miRNA_0328	GAGGCUGUAGGUUCGAGCCCUAC		X				1	7,752
candidate_miRNA_0329	GAGGUUGGGUUGGGUUGGGC			X			1	45
candidate_miRNA_0331	GCACUUCCACGUGAUCUAGACA					X	1	29
candidate_miRNA_0337	GGAGGUUCGUGUUCGAUUCAC		X				1	2,179

Table S4 Continued

candidate_miRNA_0339	GGGGCUGUAACUCAGUGGUAGAG					X	1	5,256
candidate_miRNA_0342	GGUUCGAUUCCAGUAGUGGGC		X				1	78
candidate_miRNA_0346	GUAAGAUUAUUUUGGACCCU					X	1	54
candidate_miRNA_0347	GUAAGGCCCUGUAAAAUUGUAC					X	1	21
candidate_miRNA_0348	GUAAGGCCCUGUAAAAUUGUACU				X		1	625
candidate_miRNA_0349	GUAAGUUAUGCGGACUGUGCU		X				1	30
candidate_miRNA_0358	GUACGUUUCUGGACCCCGCC		X				1	38
candidate_miRNA_0360	GUAGGAAUCCCGUAGUUAUUUC	X					1	53
candidate_miRNA_0370	GUCAAAGUUGAAGCAUGAGAACU			X			1	51
candidate_miRNA_0381	GUGAGUCGGAUAUCAGCAAGCA				X		1	171
candidate_miRNA_0382	GUGAGUCGGAUAUCAGCAAGCAG					X	1	163
candidate_miRNA_0383	GUGAGUGAUGCGAUCCGCUUG					X	1	65
candidate_miRNA_0386	GUGGCACUUUCGAAGCUUUGGC		X				1	60
candidate_miRNA_0389	GUGUAACGAUACUGUACUGGAGG		X				1	38
candidate_miRNA_0411	UAAACUAUGAAACUAUUCUGAC					X	1	1,168
candidate_miRNA_0414	UAAAGGUUUCUCUGGAUCAAACC			X			1	28
candidate_miRNA_0420	UAAAUGAUUGACCUUGAGCACG			X			1	215
candidate_miRNA_0422	UAAAUUAACGUCUACACUGAG		X				1	52
candidate_miRNA_0424	UAACCAUGGCAUAGAGCUCACA		X				1	195
candidate_miRNA_0426	UAACGUUGCCGUCAUAUCUUCC					X	1	213
candidate_miRNA_0427	UAACUAAGCGUCAAGCAGGGAC					X	1	73
candidate_miRNA_0428	UAACUAGGCGUCAAGCAGGAACU			X			1	137
candidate_miRNA_0433	UAAGAUGAUGAUUGACUAAAGU			X			1	22
candidate_miRNA_0436	UAAGCCUCACUGCACUGUGCAUG		X				1	52
candidate_miRNA_0437	UAAGCGUGACAAAUACGGCUCC		X				1	39
candidate_miRNA_0443	UAAGGCAGACUCUUCCAUAAAC		X				1	33
candidate_miRNA_0451	UAAGUCAGAUUCUGGUACUAA					X	1	72
candidate_miRNA_0452	UAAGUCGAGUUCUAUGCCAACCC			X			1	23
candidate_miRNA_0456	UAAUACAAGUAAAUCGAACUGC					X	1	33
candidate_miRNA_0459	UAAUAGUCGGCUACUUGGAAGG					X	1	45
candidate_miRNA_0462	UAAUCACAGGAAAACUCUCUAC					X	1	224
candidate_miRNA_0465	UAAUCGUAGGGUACUCGUGGCUG	X					1	25
candidate_miRNA_0466	UAAUCUUGGACGGAUAUUGGAAG			X			1	20
candidate_miRNA_0471	UAAUGGCAAACAGGACUUAAAGC		X				1	29
candidate_miRNA_0475	UAAUGUUAAAAGGAAUGACC			X			1	116
candidate_miRNA_0479	UAAUUUUUGUGGUUCUGACGAA			X			1	28
candidate_miRNA_0481	UACAAUUCGUAACUAAUAGUAGA					X	1	7,144
candidate_miRNA_0488	UACAUGAUUGCCUUCGCAGG		X				1	914
candidate_miRNA_0489	UACAUGGAAUAAAAGUUCUG		X				1	44
candidate_miRNA_0490	UACAUUUUCCCCGGACUCCAAC			X			1	20
candidate_miRNA_0501	UACCCCGCAGUCUGAAUCUAUG					X	1	56
candidate_miRNA_0502	UACCCCUUUGGUGUUGUCUGAAG		X				1	42
candidate_miRNA_0503	UACCGAGUCGACUUAGUACUGA			X			1	20

Table S4 Continued

candidate_miRNA_0517	UACCUCGAGAGCCUAGAGCAC					X	1	584
candidate_miRNA_0519	UACGAAGGUCUAUAUAAUCAAG					X	1	799
candidate_miRNA_0520	UACGAAGGUCUAUAUAAUCAUG		X				1	36
candidate_miRNA_0524	UACGAGACUAAAUGAAUGUUUA					X	1	11,210
candidate_miRNA_0525	UACGAGAGUAUCUGUAAGGCGG					X	1	116
candidate_miRNA_0527	UACGAGGAGAUUGUCUAGUACU			X			1	162
candidate_miRNA_0528	UACGAGGAGAUUGUCUAGUACUU				X		1	51
candidate_miRNA_0531	UACGGCUUCGACUUCGAUCAAC			X			1	25
candidate_miRNA_0535	UACUAAAUGAUUGAUCUUGAGC	X					1	110,269
candidate_miRNA_0536	UACUAGAUCAAGUAGAAUGUAGA			X			1	30
candidate_miRNA_0541	UACUGAUACUCAGGACCAUUACC		X				1	93
candidate_miRNA_0542	UACUGUACUGGAGGCGGCGGC		X				1	14,104
candidate_miRNA_0544	UACUUAGAAGUCUUGCGCGCUA				X		1	33
candidate_miRNA_0548	UACUUGGUCGUCUUGGAUACAC			X			1	48
candidate_miRNA_0549	UACUUUAAUUGUUGUACUAGAG			X			1	20
candidate_miRNA_0552	UAGAAUCUGAAACUUGAAGCUU			X			1	24
candidate_miRNA_0555	UAGACCUUCGUGUUCUGGCUCU			X			1	394
candidate_miRNA_0556	UAGACGGCAAACCUCGUAACAAG		X				1	41
candidate_miRNA_0557	UAGACGGCAAACCUCGUAACACA		X				1	24
candidate_miRNA_0560	UAGAGAAGAUUGUCACAAAGGAG			X			1	22
candidate_miRNA_0565	UAGAUCACGUAGAAGUGCUC		X				1	148
candidate_miRNA_0567	UAGAUCAUGUGGAAGUGCUC					X	1	20,852
candidate_miRNA_0569	UAGAUUAUAUAGACCUUCGUGC		X				1	33
candidate_miRNA_0571	UAGCACGGAUGUCAAAUUACAA					X	1	22
candidate_miRNA_0572	UAGCACGGAUGUCAAAUUACAAU			X			1	217
candidate_miRNA_0578	UAGCGGAGUGGUUAGAUAGAACG		X				1	988
candidate_miRNA_0580	UAGGAAGGAAUCUCUGGAUUCU	X					1	27
candidate_miRNA_0584	UAGGACUAGUGCCCUGUGCAUG					X	1	59
candidate_miRNA_0585	UAGGACUAGUGCCUUGUGCACG		X				1	48
candidate_miRNA_0586	UAGGACUCAGGAUGGGCUAGGA		X				1	101
candidate_miRNA_0589	UAGGAUAAUUGUUCUGGGACAAU			X			1	28
candidate_miRNA_0592	UAGGCACGAAGGACUAUAUAA		X				1	52
candidate_miRNA_0594	UAGGCAUGUCGAAGGAUUUUUC	X					1	27
candidate_miRNA_0599	UAGGGCAGACACCUCCGUAGG					X	1	370
candidate_miRNA_0601	UAGGGUGGACAUCUGAAGGAUAG		X				1	23
candidate_miRNA_0603	UAGGUAGAAAAGAAAAGGAUCU			X			1	21
candidate_miRNA_0606	UAGGUCACGUGGAAGUGCUCUA				X		1	309
candidate_miRNA_0607	UAGGUGAUCACUGGUGAUCAUG		X				1	148
candidate_miRNA_0608	UAGGUGAUCAUUGGUGAUCAUG		X				1	340
candidate_miRNA_0611	UAGUAACGCAUAGCUUGAACACC		X				1	22
candidate_miRNA_0612	UAGUACCAGAAUCUGACUUAGA					X	1	404
candidate_miRNA_0617	UAGUAUAGCUGAGAAUCUGAGA		X				1	44
candidate_miRNA_0621	UAGUGGGAAGCUGAUAGGGCAA			X			1	123

candidate_miRNA_0622	UAGUUACGAAGAUCUAUAUAGC		X				1	540
candidate_miRNA_0624	UAGUUCGAUAGUCCUGUGGUGA			X			1	20
candidate_miRNA_0627	UAGUUGUAGGUUAAGGCUUUA		X				1	53
candidate_miRNA_0628	UAGUUUAUAACUAGGCGUCAAGC					X	1	2,613
candidate_miRNA_0634	UAUACUAAAUGAUUGAUCUUGAA			X			1	36
candidate_miRNA_0635	UAUACUUUCUGGCAGCACGAG					X	1	38
candidate_miRNA_0639	UAUAUAAUAGUGUGACGGAAACC			X			1	25
candidate_miRNA_0640	UAUAUCGCUCACAUCAUGCUG			X			1	63
candidate_miRNA_0644	UAUCACUGGUAUUCACUUGAAAG		X				1	326
candidate_miRNA_0646	UAUCAUUGGUAUUCACUUGGAAG			X			1	47
candidate_miRNA_0652	UAUGACACUUUCAAGAAUUGGAUG	X					1	2,655
candidate_miRNA_0664	UAUGGCUAGUCAGGACCGCUGC		X				1	290
candidate_miRNA_0668	UAUGUACUCUGUAUGUAUGUACC		X				1	48
candidate_miRNA_0671	UAUGUAUGUAUGGAUGGAUG					X	1	31
candidate_miRNA_0673	UAUGUUGGAGAUGUUUGGAAGA			X			1	37
candidate_miRNA_0674	UAUUAAAAUGGUCUCACAACU			X			1	26
candidate_miRNA_0679	UAUUGGAGGAACUUUUUGAGCU			X			1	78
candidate_miRNA_0683	UCAAACAUUAGAAGAUUGAGGC		X				1	23
candidate_miRNA_0684	UCAAACUCUUGCACUGGUAUCC		X				1	259
candidate_miRNA_0688	UCAACAUCAGAUCCAAGACGCUU				X		1	26
candidate_miRNA_0695	UCAAGCAGGGACUUAAGUAAUAAC				X		1	42
candidate_miRNA_0696	UCAAGCAUUGUUCGUUCAGUAGC		X				1	23
candidate_miRNA_0698	UCAAGUAGAAUAUAGAUAAAC					X	1	252
candidate_miRNA_0699	UCAAGUAGAAUAUAGAUAAACU			X			1	25,941
candidate_miRNA_0700	UCAAGUAGAAUGUAAAUAAACU			X			1	160
candidate_miRNA_0703	UCAAGUAGAAUGUAGGUAGACU			X			1	97
candidate_miRNA_0704	UCAAGUAGAAUGUGGGUAAACU			X			1	172
candidate_miRNA_0705	UCAAGUAGGAUGUAGGUAAAC					X	1	2,186
candidate_miRNA_0706	UCAAGUAGGGACUUAAAGUAA		X				1	1,523
candidate_miRNA_0707	UCAAUCACUUAGAAUCUAAAAC		X				1	2,433
candidate_miRNA_0708	UCAAUUAUUUAGUAUACUCUACU			X			1	138
candidate_miRNA_0714	UCACCAGACAUAGAUAUGAGACU	X					1	90
candidate_miRNA_0715	UCACCAGACAUAGAUAUGAGACUG				X		1	23
candidate_miRNA_0717	UCACCAGUAAGCUAUGAUACCU	X					1	59
candidate_miRNA_0723	UCACGUGGAAGUGCUCCAGGC		X				1	45
candidate_miRNA_0724	UCACGUGGAAGUGCUCUAGAC		X				1	50
candidate_miRNA_0727	UCACUGAUCUGAUCUAUCUGAAG			X			1	27
candidate_miRNA_0729	UCACUGGUAUUUAUUUGGAAG		X				1	3,861
candidate_miRNA_0730	UCAGAAGAACUCAUAGAUAUUGA					X	1	115
candidate_miRNA_0731	UCAGAUCCGGACAAGUACUAGA					X	1	56
candidate_miRNA_0732	UCAGAUGUUAAGGUGGAAUAUC			X			1	24
candidate_miRNA_0735	UCAGGUAGAAUGUAGGUAAACU			X			1	71
candidate_miRNA_0739	UCAUCAUUGUCAUUACUUUAAG					X	1	711

Table S4 Continued

candidate_miRNA_0741	UCAUCGGGUCGCCAAUAUCACC		X				1	37
candidate_miRNA_0743	UCAUGCCUAUGGCUAGUCAGGACC	X					1	7,979
candidate_miRNA_0744	UCAUGGAACUCAUUUUUGUACU			X			1	24
candidate_miRNA_0756	UCCACUAGAUCACUGGCUCUUG		X				1	24,827
candidate_miRNA_0757	UCCAGUACAGUAUCGUUACAC					X	1	69
candidate_miRNA_0772	UCCUACUUGAUCUAGUAACGAC					X	1	65
candidate_miRNA_0775	UCCUCGUCCACUAGAUCACU		X				1	1,741
candidate_miRNA_0777	UCCUGGAAAGCGAAUUGUCCCU			X			1	25
candidate_miRNA_0778	UCCUGGAGCCCUGAGGAGCCU			X			1	39
candidate_miRNA_0779	UCCUGGAGGCUCCAUCACGUGA		X				1	21
candidate_miRNA_0780	UCGAAAAGUACAUUACAGGACU			X			1	24
candidate_miRNA_0784	UCGAAGUCGUACCUACAAGG					X	1	55
candidate_miRNA_0785	UCGAAGUGGUUUAUAACUAGGCG		X				1	195
candidate_miRNA_0786	UCGAAUCGAAUAUAAUAAUGACC		X				1	288
candidate_miRNA_0789	UCGAGAACUUUAAAGUAUAUAU		X				1	326
candidate_miRNA_0798	UCGAGGAUGAUUUGAAGAAUUCC					X	1	184
candidate_miRNA_0802	UCGAGGUGGUUUACAACUAGGCG		X				1	561
candidate_miRNA_0804	UCGAGGUGGUUUAUAACUAGGUG					X	1	1,806
candidate_miRNA_0805	UCGAGUAGAUACCUAGCAAAAGC		X				1	28
candidate_miRNA_0807	UCGAUGGGCUCCGGGUUCGAU					X	1	1,168
candidate_miRNA_0812	UCGCAUUCGACUGAUAGUCGUG			X			1	21
candidate_miRNA_0817	UCGGAAAGUGUGACUGAAGGACU			X			1	27
candidate_miRNA_0823	UCGGAGCUGUCGCGAUGCAGACC		X				1	26
candidate_miRNA_0825	UCGGAUACUCGCUUGAGGCGG		X				1	25
candidate_miRNA_0826	UCGGAUCAGAAACCAUAUGCGA			X			1	23
candidate_miRNA_0829	UCGGCCGGAUUGUGACUUAUGCC					X	1	257
candidate_miRNA_0835	UCGGGGAAGAUGAUAGAACGAC		X				1	28
candidate_miRNA_0836	UCGGUAAAGUAAGAUCAUUGAA					X	1	146
candidate_miRNA_0838	UCGGUCAAUUGUGAUUUUGAGG			X			1	33
candidate_miRNA_0841	UCGUAACUGAUAGUAGAGUAUA					X	1	3,010
candidate_miRNA_0843	UCGUACUUGGAUCUUCAGGAGG		X				1	24
candidate_miRNA_0844	UCGUAGGUCCAUAAACUCUCAGG		X				1	67
candidate_miRNA_0845	UCGUCAAAAUCCAUAUCUCGAGU				X		1	55
candidate_miRNA_0849	UCUACGGAAGUGCGAAGCUCUCC					X	1	245
candidate_miRNA_0852	UCUCAGAUCGUAUUGUGUUAUC			X			1	86
candidate_miRNA_0854	UCUCCGGCUUUAUGAAUGAUCAAC	X					1	54
candidate_miRNA_0856	UCUGAAAAUCCCCGAGAUUCGCC		X				1	30
candidate_miRNA_0862	UCUGGUUGGUAGUUGUAGGUUA		X				1	5,651
candidate_miRNA_0863	UCUUAGAAUUUUCGACCGGUGC					X	1	29
candidate_miRNA_0864	UCUUUGAUGGUCUAGUGGUCA					X	1	169
candidate_miRNA_0865	UCUUUUGUAUGAAUGAGAAUACC			X			1	43
candidate_miRNA_0866	UGAAAAGCUUUUGCAUCUCGGA	X					1	674
candidate_miRNA_0868	UGAAGACUAGUGAUAAAGCC					X	1	3,132

candidate_miRNA_0869	UGAAGAGCACCUGUAGAUGAGG					X	1	1,839
candidate_miRNA_0870	UGAAGGACUAUAUAAUCAAGAUG					X	1	1,086
candidate_miRNA_0875	UGAAGUAGUGUACUGUGGCAUU			X			1	53
candidate_miRNA_0879	UGAAUCCACGUCGGCUAUGGCAC		X				1	324
candidate_miRNA_0880	UGAAUGAAGCUUGAGCACGGAUG			X			1	35
candidate_miRNA_0888	UGACCAGAACCAUGACCACGACC		X				1	156
candidate_miRNA_0889	UGACCUAGGAGCCUUGAGCAUG		X				1	36
candidate_miRNA_0895	UGACUAACUUAUGGACCGCGAGA			X			1	56
candidate_miRNA_0898	UGACUGACUGACUGACUGACU			X			1	112
candidate_miRNA_0899	UGAGAAAUGGUCAAAGAAAAUAG					X	1	187
candidate_miRNA_0901	UGAGAAUUGCCUUUGAUGAUUG			X			1	29
candidate_miRNA_0908	UGAAGACUAGUGAUAAAGCCU			X			1	9,923
candidate_miRNA_0910	UGAGAUGUAUAGUACGAAGGUC					X	1	20
candidate_miRNA_0914	UGAGCUGGAAGCAACUGACAUGU			X			1	76
candidate_miRNA_0917	UGAGGACAAUGGACCAAGAUAUC			X			1	85
candidate_miRNA_0920	UGAGGAUAUAAUGAAUGACUUCU			X			1	136
candidate_miRNA_0929	UGAGGUUGAUGAUCGACAAGUU			X			1	23
candidate_miRNA_0931	UGAGUCGAUUUUCUGGGGAACU			X			1	53
candidate_miRNA_0935	UGAUAACUAAGUUGAGCACCA				X		1	28
candidate_miRNA_0938	UGAUACGGUACUUUUGGGAUGC			X			1	47
candidate_miRNA_0941	UGAUAGGGCAGAACUGAGGCUA					X	1	1,592
candidate_miRNA_0942	UGAUAGGGUAGAGACGGGAACU			X			1	197
candidate_miRNA_0943	UGAUAUGAUAUGAUAUGAUAUG		X				1	26
candidate_miRNA_0948	UGAUGCGCUGCGCUGUGAGAUC			X			1	23
candidate_miRNA_0949	UGAUGGAUAGACUGGAUAGCCU			X			1	738
candidate_miRNA_0953	UGAUGUUACGUGACGGUUGUUA			X			1	51
candidate_miRNA_0954	UGAUGUUUUAAUGUUUGAUACCC				X		1	30
candidate_miRNA_0956	UGAUUCCGGAGAUAUUGGUUCG					X	1	95,454
candidate_miRNA_0957	UGAUUCGUGACACUCUGGGACCC			X			1	82
candidate_miRNA_0959	UGAUUGAUCUUGAGCACGAAG	X					1	455
candidate_miRNA_0961	UGAUUGAUCUUGAGCAUGAAGC					X	1	6,391
candidate_miRNA_0962	UGAUUGGUAGUUGUGGGUUAAG		X				1	559
candidate_miRNA_0963	UGAUUGUAUUGUAUGAUGUAUG			X			1	64
candidate_miRNA_0965	UGAUUUGAGAACGAAUACCCU			X			1	21
candidate_miRNA_0968	UGCACACGGACAAUGAUACUCG					X	1	81
candidate_miRNA_0970	UGCACGCGUCCGUACUGUACUG		X				1	37
candidate_miRNA_0973	UGCAGCACUGUAGUAUCUACCC			X			1	30
candidate_miRNA_0974	UGCAGUUGGUAAUAGGAUCUGC					X	1	21
candidate_miRNA_0976	UGCAUCUCUCACAAGCGCGGACC		X				1	179
candidate_miRNA_0979	UGCCAUUCUCGGAUCAUCGGUA		X				1	2,726
candidate_miRNA_0983	UGCCCUGAAAGACUCUCUCGAG			X			1	23
candidate_miRNA_0985	UGCCUAAGGUUCGUCGGUUGG					X	1	450
candidate_miRNA_0986	UGCCUAAGGUUUCGUCUGGUUGA			X			1	514

candidate_miRNA_0995	UGCGGAACAAUGAAUAAUUAGU			X			1	21
candidate_miRNA_0999	UGCGGUGAUGACUCAAUUCGA					X	1	198
candidate_miRNA_1000	UGCGUAGCACGUGCACAAGGCAC					X	1	80
candidate_miRNA_1003	UGCUCCACGGUAGGCUCAGUUCU			X			1	31
candidate_miRNA_1005	UGCUCUAGGCUCUCGAAGUAGG		X				1	1,132
candidate_miRNA_1006	UGCUCUAGGCUCUCGAGGUAG		X				1	41
candidate_miRNA_1007	UGCUGGAUCACUCAGGAUCACU			X			1	182
candidate_miRNA_1025	UGGCUGUCCAGGACCGCGCAGC		X				1	742
candidate_miRNA_1026	UGGCUGUUAGACGAGAAAUGAAA			X			1	23
candidate_miRNA_1028	UGGCUUUGAUCAAGUGAAGGUC			X			1	28
candidate_miRNA_1031	UGGGCCAUUAUAUAUUACUGACC		X				1	40
candidate_miRNA_1033	UGGGGAACUCGGACACUUUUUG		X				1	75
candidate_miRNA_1034	UGGGGAUAGAUGGACGGAGUGC		X				1	32
candidate_miRNA_1035	UGGGGAUAGCUACUCUAUAUAC					X	1	24
candidate_miRNA_1042	UGGUAGUUGUGGUUAAGGCUUU				X		1	23
candidate_miRNA_1043	UGGUAUAAUGACUACUUGGUUG	X					1	73
candidate_miRNA_1048	UGUAAGGAAUAGAGCAGUAAGCU			X			1	29
candidate_miRNA_1050	UGUACCCUAUUGUAGACCGAUA		X				1	697
candidate_miRNA_1055	UGUAUUUAAAAAGUCAGGGUA		X				1	75
candidate_miRNA_1056	UGUCAGCAACUGUUGGGACGGCU			X			1	30
candidate_miRNA_1059	UGUCCUUCUCUCACAUGGCAUG		X				1	27
candidate_miRNA_1063	UGUGACUGCAAUUUCGGCUCC			X			1	42
candidate_miRNA_1072	UGUGGAAGUGCUCUAGGCUC					X	1	6,586
candidate_miRNA_1073	UGUGGAAUUCGGAUUUGGUAUG			X			1	20
candidate_miRNA_1078	UGUGUACUUGAGGAUUAUGCCCA				X		1	110
candidate_miRNA_1080	UGUGUAGGCAUAUUAAUCUCC		X				1	491
candidate_miRNA_1081	UGUGUAGGCAUAUUAAUCUCCU			X			1	1,548
candidate_miRNA_1084	UGUGUGGUCGAGUUUGUCAACA		X				1	23
candidate_miRNA_1085	UGUUACGUGACGGUUGUUACCC					X	1	26
candidate_miRNA_1086	UGUUCUGAUUAGAAGUCGCGGCC		X				1	131
candidate_miRNA_1088	UGUUGGCACACUCUUCCAGAGC		X				1	33
candidate_miRNA_1091	UGUUUUUCUGAAUUGCCUUGG		X				1	142
candidate_miRNA_1096	UUAACUAGGAAUCGUCGUUCC		X				1	184
candidate_miRNA_1097	UUAACUUAGAAUCUACAGGUA			X			1	2,011
candidate_miRNA_1098	UUAAGGAUUUGGUAGCAUCACC			X			1	22
candidate_miRNA_1101	UUAAUCUAGAACCUUGAGCAUG			X			1	81
candidate_miRNA_1103	UUAAUUGUUGGUGUGAUCUGU		X				1	64
candidate_miRNA_1104	UUAAUUGUUGGUGUGAUCUGUU			X			1	318
candidate_miRNA_1107	UUACGAGAACUGAUCAUACGAGA		X				1	26
candidate_miRNA_1108	UUACGAGAUACGAUUUGACCG			X			1	146
candidate_miRNA_1110	UUACGAGGUUUGCUGUCUAGA		X				1	64
candidate_miRNA_1116	UUAGAGCAUACAUGUCAUCAUG		X				1	112
candidate_miRNA_1117	UUAGAUACUCGUAAUUGAAUGA			X			1	23

Table S4 Continued

candidate_miRNA_1119	UUAGCAUGAAGGUCUAUAUAACC		X				1	74
candidate_miRNA_1127	UUAUAACUAGACGUCAAGCAGA			X			1	596
candidate_miRNA_1129	UUAUAAUAUAGUUCGUAAGUCU			X			1	93
candidate_miRNA_1132	UUAUAGCAGACUUGGAUUUAAU	X					1	20
candidate_miRNA_1134	UUAUAUAGACCUUCGUACUAUA			X			1	22
candidate_miRNA_1140	UUAUGUAGGACUUGGUAGGUAUA					X	1	247
candidate_miRNA_1144	UUAUUAAAAGAUUGGGUGGAAG					X	1	21
candidate_miRNA_1147	UUCAAACCGAAAUCUGGCGCCU				X		1	63
candidate_miRNA_1151	UUCAAUCUGGGUUCGAUUCCC		X				1	501
candidate_miRNA_1155	UUCACUUUACUCUUGAUCCUUG		X				1	99
candidate_miRNA_1157	UUCAGAGACUCAGAGUAUACUG				X		1	73
candidate_miRNA_1161	UUCAUAGGUGUAGCUGGCCAUG			X			1	45
candidate_miRNA_1163	UUCAUCGUAGGCAGUCUGGUGC					X	1	62
candidate_miRNA_1166	UUCCAAUGGAUCACAUGAACUG			X			1	29
candidate_miRNA_1167	UUCCAAUUGUUCCUUCUGAAA					X	1	101
candidate_miRNA_1170	UUCCAGGCAGGACAUUUGAACC					X	1	26
candidate_miRNA_1172	UUCCCGAUUCCUCUGUUCCUG		X				1	41
candidate_miRNA_1173	UUCCCGAUUCCUCUGUUCCUGU	X					1	64
candidate_miRNA_1176	UUCCGGAAGUCGAUCGACUCU		X				1	24
candidate_miRNA_1177	UUCCGGAAGUCGAUCGACUCUU	X					1	112
candidate_miRNA_1180	UUCCUCAAUAUUCACUCUGGUC		X				1	27
candidate_miRNA_1186	UUCGAUCGUCAUUUUUCUGCUU			X			1	58
candidate_miRNA_1190	UUCGGAGGAGAAAUUUUGAUGU			X			1	138
candidate_miRNA_1192	UUCGGCCGGAUUGUGACUUAUGC				X		1	30
candidate_miRNA_1196	UUCGGUCUCGCUCUUUGCUCU			X			1	34
candidate_miRNA_1204	UUCUAAGUGAUUGAAUUGAC		X				1	177
candidate_miRNA_1206	UUCUAUUUGAUCUAGUAACGAC					X	1	93
candidate_miRNA_1211	UUCUGUUAAGCAACUCUGCACC		X				1	80
candidate_miRNA_1213	UUCUUCGUGUUACUACCUAUC		X				1	2,403
candidate_miRNA_1214	UUGAAAGGAAUCAUCGGAUUGA			X			1	22
candidate_miRNA_1221	UUGAGAACUCGUCAGCUGAUC		X				1	178
candidate_miRNA_1229	UUGAGUCAAUUCUGUCGUCCACA		X				1	20
candidate_miRNA_1231	UUGAUUAUAUAGACCUUCGUA		X				1	106
candidate_miRNA_1232	UUGAUUAUAUAGACCUUCGUAC			X			1	37
candidate_miRNA_1233	UUGAUUAUAUAGACCUUCGUGU		X				1	226
candidate_miRNA_1234	UUGAUUAUAUAGACCUUUGUG		X				1	57
candidate_miRNA_1235	UUGAUUAUAUAGUCCUUCAUG					X	1	1,805
candidate_miRNA_1236	UUGAUUAUAUAGUCCUUCGUGC		X				1	355
candidate_miRNA_1240	UUGCAAGGAAUGAACCUAUCCU			X			1	58
candidate_miRNA_1246	UUGCAUGAGAUUGAUUCCAGAC					X	1	64
candidate_miRNA_1247	UUGCAUGAGAUUGAUUCCAGACU	X					1	89
candidate_miRNA_1254	UUGCGCGACGGUUAAAUCUUAAG					X	1	45
candidate_miRNA_1255	UUGCGCGACGGUUAAAUCUUAAGU				X		1	30

candidate_miRNA_1265	UUGGACUAGAUAUUGAUAUCAC					X	1	26
candidate_miRNA_1268	UUGGAUAGCCUGGAUAGACUGG					X	1	1,283
candidate_miRNA_1271	UUGGCAGAGAAUUCUUGAAAGG			X			1	25
candidate_miRNA_1272	UUGGCAGCUGAAGACUUGACACC					X	1	27
candidate_miRNA_1283	UUGGGCUGGUAGGCCUUUUGGGGU				X		1	42
candidate_miRNA_1287	UUGGGGCGGAGGGACAGAAUUUC			X			1	21
candidate_miRNA_1291	UUGGGUUGUGGGCAGUGGGCA		X				1	70
candidate_miRNA_1293	UUGGUAGUAGAUUCAGUUGCAUG		X				1	87
candidate_miRNA_1298	UUGGUUAUAUAGAUAUUCAUGCU			X			1	22
candidate_miRNA_1299	UUGGUUGGAUGACAGCAAUGCAG		X				1	63
candidate_miRNA_1301	UUGGUUUCCUCGUCUCGGACGC					X	1	20
candidate_miRNA_1302	UUGUAACGAUACUGUACUGGAGG		X				1	1,306
candidate_miRNA_1313	UUGUAUCAUAGAAAGUUUGACCU			X			1	30
candidate_miRNA_1314	UUGUAUUUAACUCAGAAUCUACA		X				1	57
candidate_miRNA_1317	UUGUCACACUCAAGAAUACUGG					X	1	61
candidate_miRNA_1318	UUGUCAGAAUAUCUAGGACUUCC			X			1	53
candidate_miRNA_1321	UUGUCGCUGGUCGUCAUGGCGCC				X		1	33
candidate_miRNA_1323	UUGUCUGAUUGUAUUGCCUUGC					X	1	35
candidate_miRNA_1339	UUUCAUUUCUUAGAGCCUGUCU			X			1	28
candidate_miRNA_1340	UUUCAUUUCUUAGAGCCUGUCUA				X		1	23
candidate_miRNA_1343	UUUCCGCCGCGUUCGAGUACUG		X				1	103
candidate_miRNA_1345	UUUCCUACUUCAGAUAUUCUGC					X	1	43
candidate_miRNA_1347	UUUCCUAUUCGGUCUAGCUGUG					X	1	22
candidate_miRNA_1352	UUUCGGGUUGAGCCAUACAUCC					X	1	44
candidate_miRNA_1355	UUUCUCUCAGGUCUUGAGCAUG			X			1	23
candidate_miRNA_1357	UUUCUGGGAACUAUCAAGAGCU			X			1	34
candidate_miRNA_1359	UUUCUUGACUGUAUUUCCCCGG					X	1	73
candidate_miRNA_1368	UUUGGACAAAUGACGCUCAGA		X				1	505
candidate_miRNA_1372	UUUGGCGGAGUGGUUAACGC					X	1	9,412
candidate_miRNA_1374	UUUGUAGAGCUUCGUGCUCAAGA		X				1	45
candidate_miRNA_1376	UUUUGAGGACCUUUGGGACCCU	X					1	655
candidate_miRNA_1377	UUUUGGACUAGAUAUUGAUAUCA				X		1	43
candidate_miRNA_1380	UUUUUCUUCUUCCAAGGGCUU			X			1	31

Table S5. Targets predicted for *Sclerotinia sclerotiorum* small RNAs by degradome sequencing

Locus	Sequence	Length	Small RNA Reads	Log2 Fold Change smRNA	Direction Change	Predicted Target	Degradome Reads	Degradome *P* value	Degradome Category	Log2 Fold Change Target	Annotation
candidate_miRNA_0959	UGAUUGAUCUUGAGCACGAAG	21	85,819	NS	NS	SS1G_09852	83	2.80E-02	0	NS	Hypothetical protein
candidate_miRNA_0075	UCGAGGUGGUUUAUAACUAGACG	23	30,174	1.48	down	SS1G_13086	773	6.76E-04	0	NS	Hypothetical protein
candidate_miRNA_0908	UGAAGACUAGUGAUAAAGCCU	21	9,923	NS	NS	SS1G_04502	86	1.07E-02	0	NS	Ammonium transporter-like protein
candidate_miRNA_1235	UUGAUUAUAUAGUCCUUCAUG	21	1,805	1.14	down	SS1G_13405	107	6.75E-04	0	NS	Hypothetical protein
candidate_miRNA_1231	UUGAUUAUAUAGACCUUCGUA	21	106	NS	NS	SS1G_13405	107	6.76E-04	0	NS	Hypothetical protein
candidate_miRNA_1268	UUGGAUAGCCUGGAUAGACUGG	22	1,283	1.84	down	SS1G_01281	37	1.21E-02	0	NS	Hypothetical protein
candidate_miRNA_1376	UUUUGAGGACCUUUGGGACCCU	22	655	NS	NS	SS1G_10378	188	3.84E-02	0	NS	Hypothetical protein
candidate_miRNA_0037	CGGAGGUGUGAGGCGUCACAG	21	157	NS	NS	SS1G_12523	123	4.72E-03	0	NS	Acid--D-amino-acid ligases (peptide synthases).
candidate_miRNA_0051	UAGACGGCAAACCUCGUAACAAA	23	97	1.26	down	SS1G_13438	654	6.76E-04	0	NS	Hypothetical protein
candidate_miRNA_0854	UCUCCGGCUUUAUGAAUGAUCAAC	24	54	1.88	down	SS1G_00360	187	6.06E-03	0	NS	Hypothetical protein
candidate_miRNA_0197	UAUAUAGUCCUUCAUGCUUCCA	22	52	1.05	down	SS1G_10028	29	2.40E-02	0	NS	Hypothetical protein
candidate_miRNA_0370	GUCAAAGUUGAAGCAUGAGAACU	23	51	NS	NS	SS1G_11403	65	1.01E-02	0	NS	Hypothetical protein
candidate_miRNA_0129	UAGCCUAGGAAUUCUGUUUCUG	22	43	2.06	up	SS1G_08758	134	3.39E-02	0	NS	Putative cytokinin riboside 5'-monophosphate phosphoribohydrolase
candidate_miRNA_0556	UAGACGGCAAACCUCGUAACAAG	23	41	NS	NS	SS1G_03775	53	1.35E-03	0	NS	Hypothetical protein
candidate_miRNA_0835	UCGGGGAAGAUGAUAGAACGAC	22	28	NS	NS	SS1G_12103	94	6.76E-04	0	NS	Retrovirus-related polymerase
candidate_miRNA_0659	UAUGGACAGUAUGUGUACUUCC	22	28	1.38	down	SS1G_12056	34	6.31E-04	1	NS	Putative extracellular membrane protein
candidate_miRNA_1287	UUGGGGCGGAGGGACAGAAUUUC	23	21	5.47	down	SS1G_00122	46	8.08E-03	0	NS	Hypothetical protein
candidate_miRNA_0466	UAAUCUUGGACGGAUAUUGGAAG	23	20	1.86	down	SS1G_01841	54	6.74E-03	0	NS	Hypothetical protein
cluster_08340	UAGGCGUCAAGCAGGGACUUAAAA	24	24,422	1.77	down	SS1G_03032	314	6.75E-04	0	NS	Hypothetical protein
cluster_21137	UAACACUCUGAUAGGAGUCGU	21	10,886	1.70	down	SS1G_00334	736	6.75E-04	0	NS	Probable Argonaute-2 potein
cluster_10116	UGGGGGUCUGUGUUAAAUUGAUC	23	5,870	1.68	down	SS1G_12812	102	1.41E-02	0	2.59	Pachytene checkpoint protein 2
cluster_26575	CGGAGGUGUGAGGCGUCACACA	22	2,761	1.91	down	SS1G_12523	123	6.73E-03	0	NS	Acid--D-amino-acid ligases (peptide synthases).
cluster_06232	UUAUUUUAUUUUGUCUGAAU	20	808	NS	NS	SS1G_02664	107	9.41E-03	0	NS	Hypothetical protein
cluster_14869	GAUGAUUUCGUAGCGUAUUA	20	387	2.15	down	SS1G_10754	348	4.81E-02	0	NS	Ribose-phosphate pyrophosphokinase.
cluster_07715	UUGAUUAUAUAGACCUUUGUAUU	23	199	2.69	down	SS1G_13405	107	6.75E-04	0	NS	Hypothetical protein

Table S5 Continued

cluster_25295	UUUGAUAUUGGCCUUUGGACUG	22	179	NS	NS	SS1G_10820	127	2.34E-02	0	NS	Hypothetical protein
cluster_08393	UGCCGAAUGUAGCUCUUCUGGG	22	165	1.08	up	SS1G_01499	1139	2.14E-02	0	NS	Cytochrome P450 monooxygenase apf8
cluster_12191	UAUCAUAAUUUACUGUUGACCU	22	106	4.16	up	SS1G_04937	24	1.27E-02	0	NS	Hypothetical protein
cluster_18802	UCACUGUGACGCCUCACACCU	21	106	NS	NS	SS1G_05027	2176	9.41E-03	0	2.59	Hypothetical protein
cluster_21866	UAGACUGGUCUACCUGGGCUAUC	23	89	1.53	down	SS1G_01849	103	6.06E-03	0	NS	Lipase 2-like protein
cluster_08945	UGAUGCAUACGGUACGUGCUGA	22	77	1.16	down	SS1G_09840	165	3.37E-03	0	NS	Pyroglutamyl-peptidase I.
cluster_18095	AGAUCUGAUGUACUCUGAUC	20	74	1.81	down	SS1G_04137	67	3.91E-02	0	3.15	Hypothetical protein
cluster_15735	UGUACAGAAUGGUUGGUUCGCU	22	61	NS	NS	SS1G_03983	173	1.34E-02	0	NS	Bifunctional polynucleotide phosphatase/kinase
cluster_21716	UUGAGGUGGUUUAUAACUAAGC	22	47	NS	NS	SS1G_13086	773	6.75E-04	0	NS	Hypothetical protein
cluster_07598	UAAGUAGGAUUUUGAAUAACAUC	23	46	1.04	down	SS1G_06728	57	2.02E-03	0	NS	Efflux pump roqT
cluster_13250	GAGGUUGGGUUGGGUUGGGC	20	45	NS	NS	SS1G_04054	83	4.29E-02	0	NS	Histone acetyltransferase.
cluster_25922	AAAUCCCCACACUCUCAGGACU	22	44	2.03	down	SS1G_04240	266	1.41E-02	0	NS	Hypothetical protein
cluster_17538	UAGGUAUUUAGAAUGUAGAUCC	22	39	NS	NS	SS1G_00310	340	1.81E-02	0	NS	Xaa-Pro aminopeptidase.
cluster_26063	UGUAUUCUCUCGUCAGGAUAUG	22	34	1.21	down	SS1G_13309	78	6.06E-03	0	NS	Hypothetical protein
cluster_10053	UACCGCGUAGAAUUCUAUGACU	22	31	1.20	down	SS1G_09144	71	1.41E-02	0	NS	Hypothetical protein
cluster_02494	UCCCCGUCGCAUGAUGAAUUCA	22	28	1.22	up	SS1G_03220	78	8.07E-03	0	1.97	Hypothetical protein
cluster_01312	UAUUUUUAAGGAGUAUAAGUCU	22	26	NS	NS	SS1G_02682	543	3.32E-02	0	NS	Mannan endo-1,6-beta-mannosidase.
